# Cardiac energetics in health and disease: a meta-analysis

**DOI:** 10.1093/ehjimp/qyag110

**Published:** 2026-06-17

**Authors:** Jonathan J H Bray, John A Henry, Archit Singhal, Takanayi Mureyi, Liam S Couch, Mahmood Ahmad, Ladislav Valkovič, Stefan Neubauer, Andrew J M Lewis, O J Rider

**Affiliations:** Oxford Heart Centre, Oxford University Hospitals, NHS Trust, Oxford, UK; Institute of Health Informatics, University College London, London, UK; Oxford Centre for Clinical Magnetic Resonance Research, Division of Cardiovascular Medicine, Radcliffe Department of Medicine, University of Oxford, John Radcliffe Hospital, Oxford OX3 9DU, United Kingdom; Oxford Centre for Clinical Magnetic Resonance Research, Division of Cardiovascular Medicine, Radcliffe Department of Medicine, University of Oxford, John Radcliffe Hospital, Oxford OX3 9DU, United Kingdom; Oxford Heart Centre, Oxford University Hospitals, NHS Trust, Oxford, UK; Oxford Heart Centre, Oxford University Hospitals, NHS Trust, Oxford, UK; Department of Cardiology, Royal Free London NHS Foundation Trust, London, UK; Oxford Centre for Clinical Magnetic Resonance Research, Division of Cardiovascular Medicine, Radcliffe Department of Medicine, University of Oxford, John Radcliffe Hospital, Oxford OX3 9DU, United Kingdom; Department of Imaging Methods, Institute of Measurement Science, Slovak Academy of Sciences, Bratislava, Slovakia; Oxford Centre for Clinical Magnetic Resonance Research, Division of Cardiovascular Medicine, Radcliffe Department of Medicine, University of Oxford, John Radcliffe Hospital, Oxford OX3 9DU, United Kingdom; Oxford Centre for Clinical Magnetic Resonance Research, Division of Cardiovascular Medicine, Radcliffe Department of Medicine, University of Oxford, John Radcliffe Hospital, Oxford OX3 9DU, United Kingdom; Oxford Centre for Clinical Magnetic Resonance Research, Division of Cardiovascular Medicine, Radcliffe Department of Medicine, University of Oxford, John Radcliffe Hospital, Oxford OX3 9DU, United Kingdom

**Keywords:** energetics, PCr/ATP, phosphorus spectroscopy, MRS, cardiac energy metabolism, magnetic resonance imaging

## Abstract

**Aims:**

The cardiac phosphocreatine-to-ATP ratio (PCr/ATP) is the principal non-invasive biomarker of myocardial energetic status and is measured by phosphorus magnetic resonance spectroscopy (^31^P-MRS). However, studies of cardiac energetics are typically from single centres with relatively small numbers of patients, leading to unknown generalizability.

**Methods and results:**

Here, we use systematic review and meta-analysis of all available studies reporting cardiac PCr/ATP ratios to show pooled reference values across populations (databases searched: Embase, Medline, CENTRAL). We collected data on differing methodological factors to enable adjustment. We included 176 studies comprising 7843 individual participant magnetic resonance examinations between 1987 and 2025. The pooled PCr/ATP ratio was 1.96 for healthy volunteers (*n* = 2988), 1.54 for heart failure with reduced ejection fraction (*n* = 575; a 21% reduction), and 1.60 for heart failure with preserved ejection fraction (*n* = 146; an 18% reduction) with further reductions seen across other disease states including valvular disease and diabetes. Methodological factors, including field strength, sequence type, and voxel location significantly, influenced measurements, though adjustment had minimal effect on pooled estimates. Body mass index and natriuretic peptides negatively correlated with PCr/ATP, whilst left ventricular systolic function showed positive correlation.

**Conclusion:**

This comprehensive meta-analysis provides reference PCr/ATP values across cardiac conditions, implicating energetic impairment as a common pathway spanning myocardial diseases of diverse aetiology, and suggests that methodological heterogeneity does not fully account for the observed between-group differences.

**Registration:**

PROSPERO (CRD420251081946).

## Graphical Abstract

**Graphical Abstract:**
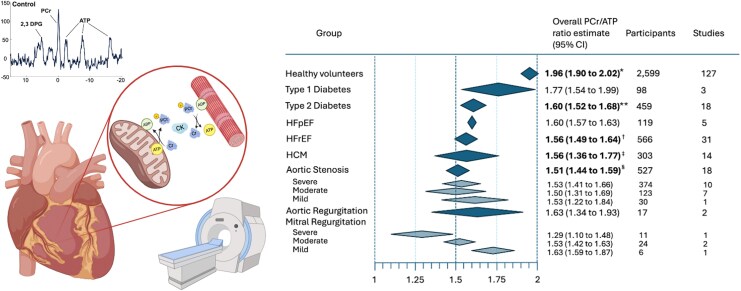

## Introduction

The heart is the most energetically demanding organ, cycling 20–30 times its weight in adenosine tri-phosphate (ATP) per day.^1^ An intricate, three-stage metabolic landscape has developed in order to sustain this ATP demand. This comprises substrate selection, oxidative phosphorylation, and finally transfer of high energy phosphates to the myofibrils for utilization as ATP. This is of critical importance for both myocardial systolic and diastolic function. Derangements in these metabolic pathways converge on a common endpoint: impaired ATP delivery to the myofibrils, a recognized hallmark of heart failure irrespective of aetiology.^1^

Non-invasive assessment of the bioenergetic capacity of the heart has been enabled over the last three decades using phosphorus cardiac magnetic resonance spectroscopy (^31^P-MRS), to quantify the phosphocreatine to ATP (PCr/ATP) ratio. Upon production of ATP at the mitochondria, the creatine kinase system transfers a phosphate group from ATP to creatine, forming phosphocreatine (PCr). PCr subsequently transfers its phosphate to adenosine di-phosphate (ADP) at the myofibril, rapidly replenishing ATP at sites of high utilization. This functions not only as a spatial transfer system but also as an energy reserve, as the reaction catalysed by cytosolic creatine kinase favours ATP regeneration from PCr and ADP, ensuring rapid local ATP supply during periods of high demand. Given this, changes in PCr tend to be a sensitive marker of bioenergetic derangements within the myocardium, with falls in PCr preceding falls in ATP.

Decreases in PCr/ATP as measured by ^31^P-MRS have been reported in both heart failure with reduced ejection fraction (HFrEF) and heart failure with preserved ejection fraction (HFpEF) of varying aetiologies,^2–4^ precursors to heart failure, such as obesity,^5^ hypertension,^6^ and diabetes^7^ and has been shown to correlate with both morbidity and mortality in those living with heart failure.^2,8^ As such, PCr/ATP ratio is considered to be a prognostic proxy for the energetic state of the myocardium. Therefore, PCr/ATP ratio is a relatively proximal marker of disease, with low intra-study variability, and is increasingly being assessed as a potential non-invasive biomarker of response to pharmacological therapy in early-phase trials.^9,10^ So far PCr/ATP is mostly confined to research and there is a lack of consensus on what is considered an expected value for a given condition and whether data using different equipment or methodologies is comparable.

Many studies have reported on PCr/ATP in both healthy human hearts and those with myocardial or systemic disease. However, there is significant methodological heterogeneity related to MR scanner platforms, sequences, coil technologies, and post-processing methods. It remains unknown how, and to what extent, these factors affect the measured PCr/ATP ratio, and indeed what constitutes normal in a healthy myocardium. This systematic review and meta-analysis seeks to consolidate and provide a compendium of all studies reporting PCr/ATP, providing values for both healthy and diseased myocardium, as well as reporting differences across imaging methodologies.

## Methods

### Search strategy

**Figure 1:**
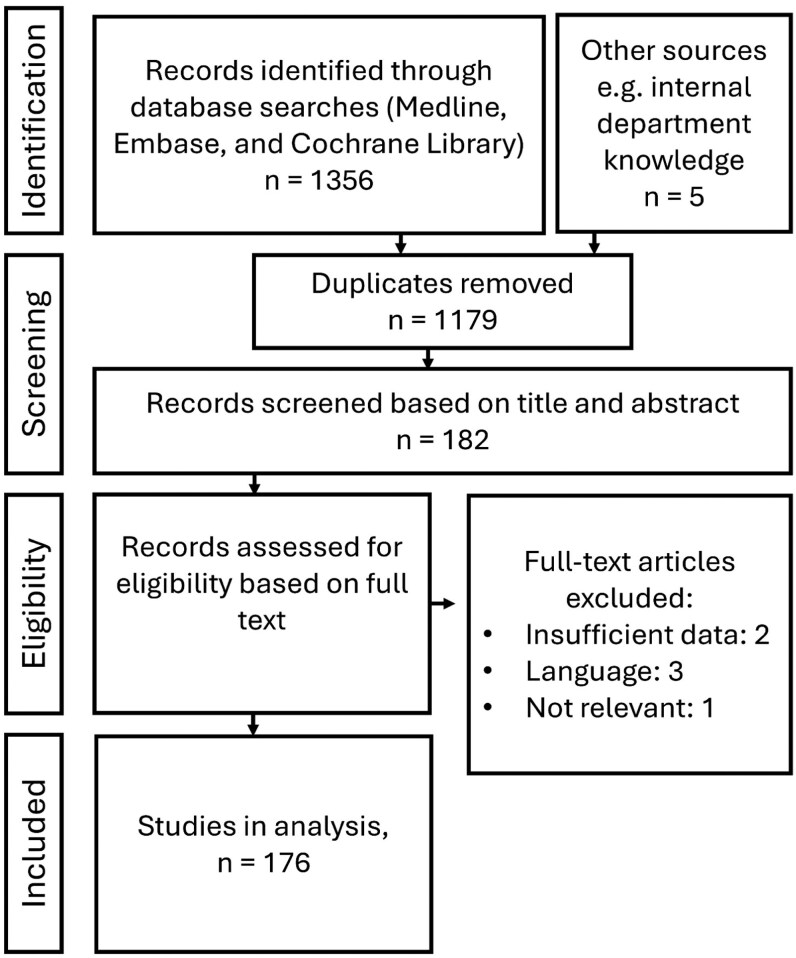
PRISMA flow sheet.

MEDLINE, Embase, and the Cochrane Central Register of Controlled Trials (Central) were systematically searched for relevant articles from inception to 1 August 2025. Our pre-specified protocol is published on PROSPERO (CRD420251081946). The findings are reported in accordance with the Preferred Reporting Items for Systematic Reviews and Meta-Analyses statement (*Figure 1*). The systematic review and meta-analysis did not involve accessing or otherwise processing patient-identifiable information and hence did not require ethical approval.

Our inclusion criteria sought report of any study that obtained a PCr/ATP ratio using ^31^P MRS. Our search strategy can be found in supplementary data online, table  *S1*. We excluded case reports and studies not reported in English due to the risk of misunderstanding methodology. We did not include in the meta-analysis studies with missing PCr/ATP ratio average or a statistic of spread. At least two reviewers (J.J.H.B., J.A.H., A.S., and T.M.) independently screened each title retrieved for relevance and then assessed full texts for eligibility. Disagreements were resolved by involvement of at least one third reviewer (J.J.H.B. and J.A.H.). Duplicates were excluded, and multiple reports from the same study were collated.

### Data extraction

Data were extracted on a pre-designed excel spreadsheet. This included where available, key participant factors, such as age, sex, natriuretic peptide level, left ventricular ejection fraction (LVEF) and body mass index (BMI), and key technical parameters, such as field strength, repetition time (TR), sequence, and voxel localization. Year of publication was used as a proxy for unmeasurable technological changes. If data were available in a figure, quantitative data were extracted using plotdigitizer.com. Where mean or standard error was not available, standard methodology as described in the Cochrane handbook was used to estimate these values. Imputation was not used.

### Data analysis

Overall average estimates and 95% confidence intervals (CI) were obtained from published data using random-effects meta-analysis using Stata (18.5, StataCorp LLC, College Station, TX, USA). The meta set command was used, utilizing the default REML method to estimate Tau^2^.^11^ Tau^2^ was used to quantify heterogeneity as compared with I^2^, Tau^2^ is more stable in large meta-analyses. Small-study bias was assessed for using funnel plots. To assess the effect of moderators on PCr/ATP ratio, we performed random-effects meta-regression using the REML method. Descriptive data are given as weighted averages.

Anticipating that methodological differences would influence the PCr/ATP ratio between studies, we performed multiple meta-regression (random-effects, REML) incorporating all covariates shown to significantly affect the overall PCr/ATP ratio estimates. We used dummy variables for categorical variables such as sequence and position, whereas field strength and year of publication was treated as continuous variables. As default we left out the first dummy variable to avoid collinearity. We ensured to use >8 studies per included covariate, treating each dummy variable as an additional covariate. If there were fewer studies than significant covariate variables, we excluded the least strongly correlated variables.

Having established that some methodological practices gave significantly different results, including sequence and field strength, we sought to adjust for these using multiple-meta regression and the margins command. Using this, we predicted the adjusted PCr/ATP ratio for each study using this model, and subsequently used in meta-analysis to give an estimate of PCr/ATP ratio that accounts for covariates. Cohen’s κ (IBM SPSS 29) was used to assess inter-reviewer agreement.

Small study bias was assessed for visual review of funnel plots and the use of Egger’s regression where the plot appeared suspicious (see Supplementary data online, *Figure S6A*–*H*).

### Quality assessment

Given the number of included studies, we adapted a validated method using large language models to act as a second reviewer to help facilitate assessment of risk of bias.^12^ Risk of bias was assessed using a validated tool for a population average.^13^ Studies were considered to have a small study size if a relevant study arm had ≤ 10 participants. Risk of bias assessment was performed by one clinician reviewer (J.J.H.B.), and GPT5 was used as the second reviewer. Disagreements were resolved by involvement of a third reviewer (J.A.H.).

### Sensitivity analysis

We performed sensitivity analysis including only low risk of bias studies and using fixed-effects meta-analysis.

## Results

**Figure 2:**
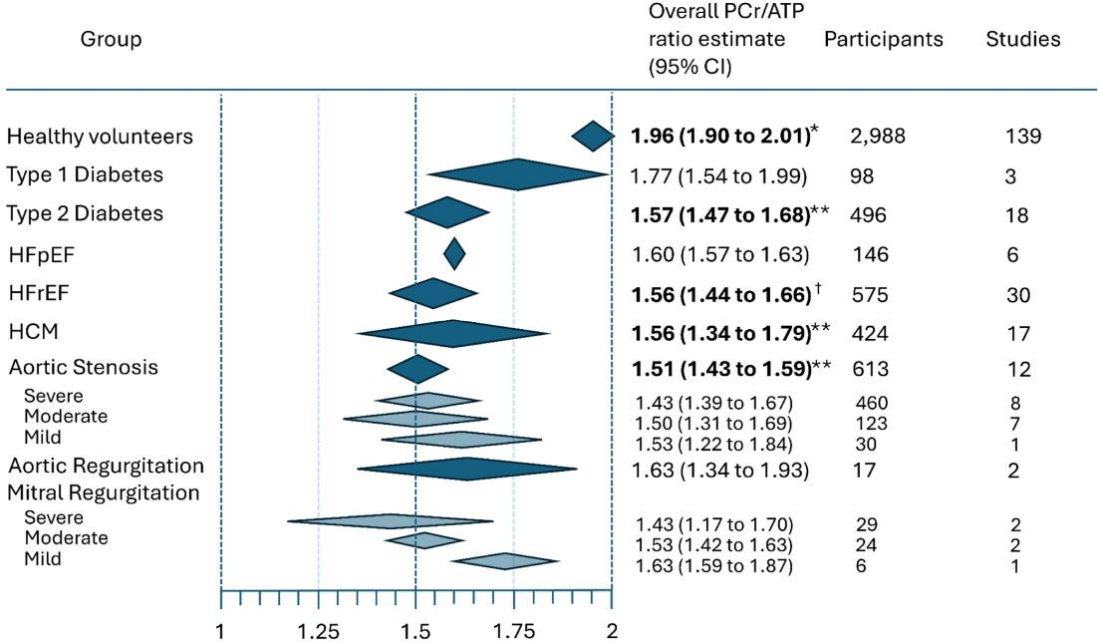
Aggregate forest plot showing overall estimates of PCr/ATP ratio for each group compared. Estimates in bold are adjusted. Extent of adjustment is limited statistically by number of studies: *Field strength, sequence, and year of publication; **Field strength and year of publication, †Sequence and year of publication. No overall for MR reported as evidence of difference between severity categories.

This review includes 7843 individual participant CMRs from 176 studies ranging in publication date from 1987 to 2025. We include 143 full texts and 33 abstracts. A summary of overall PCr/ATP ratio estimates can be found in *Figure 2*, including adjustment for methodological factors (see section below). A table of all included studies and the study populations can be found in Supplementary data online, *Table S2*. Moreover, for disease states not described below, a compendium of all reported cardiac energetic studies for cardiac PCr/ATP in cardiovascular disease can be found in Supplementary data online, *Table S3*, and cardiac PCr/ATP in systemic disease and physiological states can be found in Supplementary data online, *Table S4*.

### Variables associated with PCr/ATP ratio

**Figure 3:**
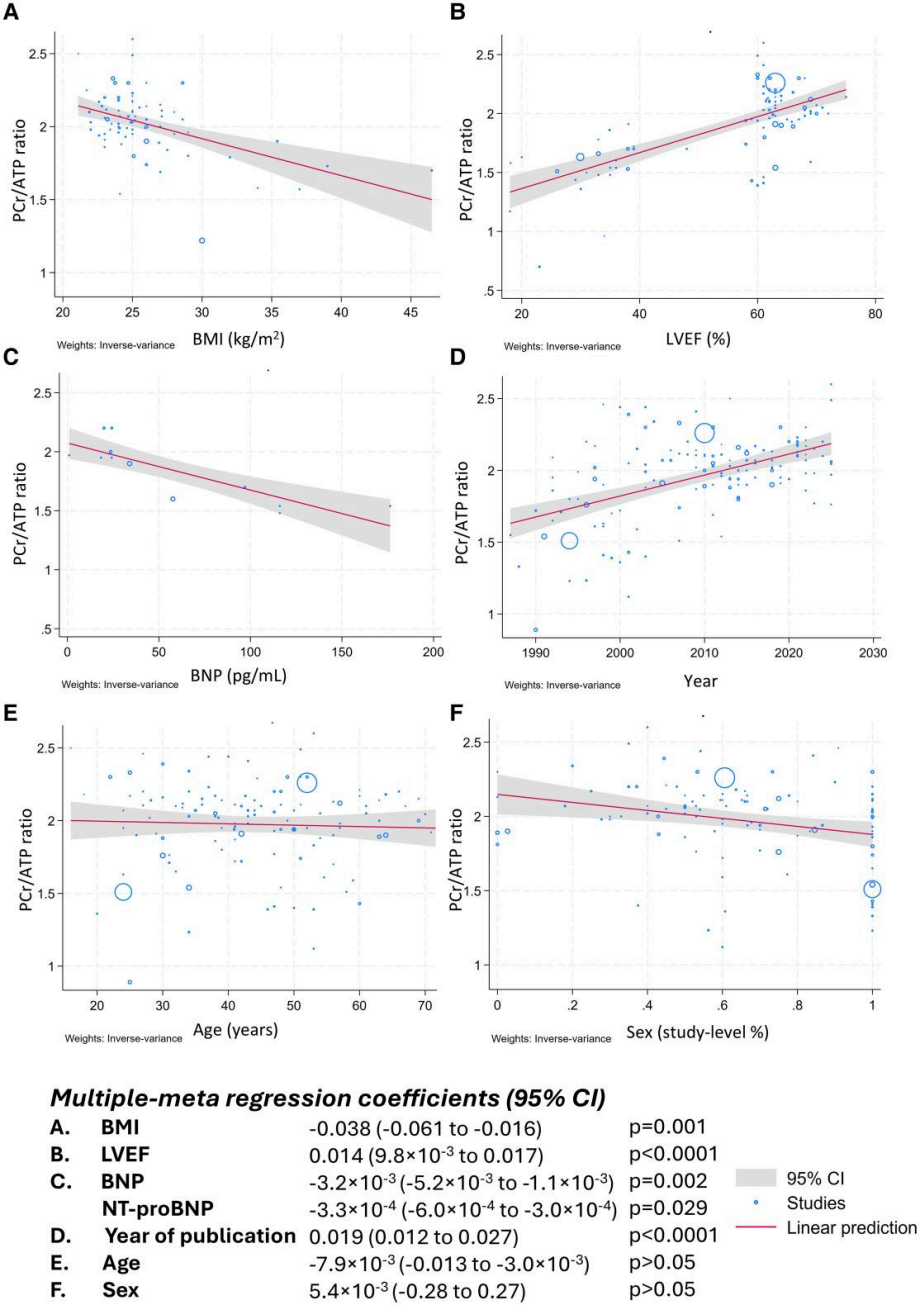
Meta-regression of factors associated and not associated with PCr/ATP ratio. Stated coefficients account for field strength, sequence, and year of publication and derive from multiple meta-regression models.

We used multiple meta-regression accounting for methodological factors influencing PCr/ATP ratio to explore whether study-level characteristics were associated with PCr/ATP ratio (*Figure 2*). In healthy volunteers and obese subjects, BMI was negatively correlated with PCr/ATP [coefficient −0.038 (95% CI −0.061 to −0.016), *Figure 3A*]. In healthy volunteers and HFrEF, LVEF was positively correlated with PCr/ATP ratio [coefficient 0.014 (9.8 × 10^−3^ to 0.017), *Figure 3B*] and both BNP and NT-proBNP were negatively correlated with PCr/ATP ratio [BNP: coefficient −3.2 × 10^−3^ (−5.2 × 10^−3^ to −1.1 × 10^−3^), *Figure 3C*; NT-proBNP: coefficient −3.3 × 10^−4^ (−6.0 × 10^−4^ to −3.0 × 10^−4^)]. Without adjustment NT-proBNP was not significantly correlated. In healthy volunteers, year of publication was significantly positively correlated with PCr/ATP ratio [coefficient 0.019 (95% CI 0.012–0.027), *Figure 3D*]. However, in healthy volunteers, sex and age were not significantly correlated with PCr/ATP ratio (*Figure 3E & 3F*).

### Healthy volunteers

A total of 139 studies reported PCr/ATP ratios in a total of 2988 healthy volunteers, mostly for the purpose of control subjects in comparison with disease states or an intervention. Participants had an average age of 46 years and were predominantly male (67%); 27 studies only included males. In random meta-analysis, the overall estimate PCr/ATP ratio at rest for healthy volunteers was 1.96 (95% CI 1.91–2.01, Supplementary data online, *Figure S1*). After adjustment for field strength, position, and sequence, the overall estimate remained unchanged at 1.96 (95% CI 1.90–2.01, *Figure 2*). Adjustment successfully reduced heterogeneity from moderate to very low levels (Tau^2^ 0.08–0.02).

### Heart failure

**Figure 4:**
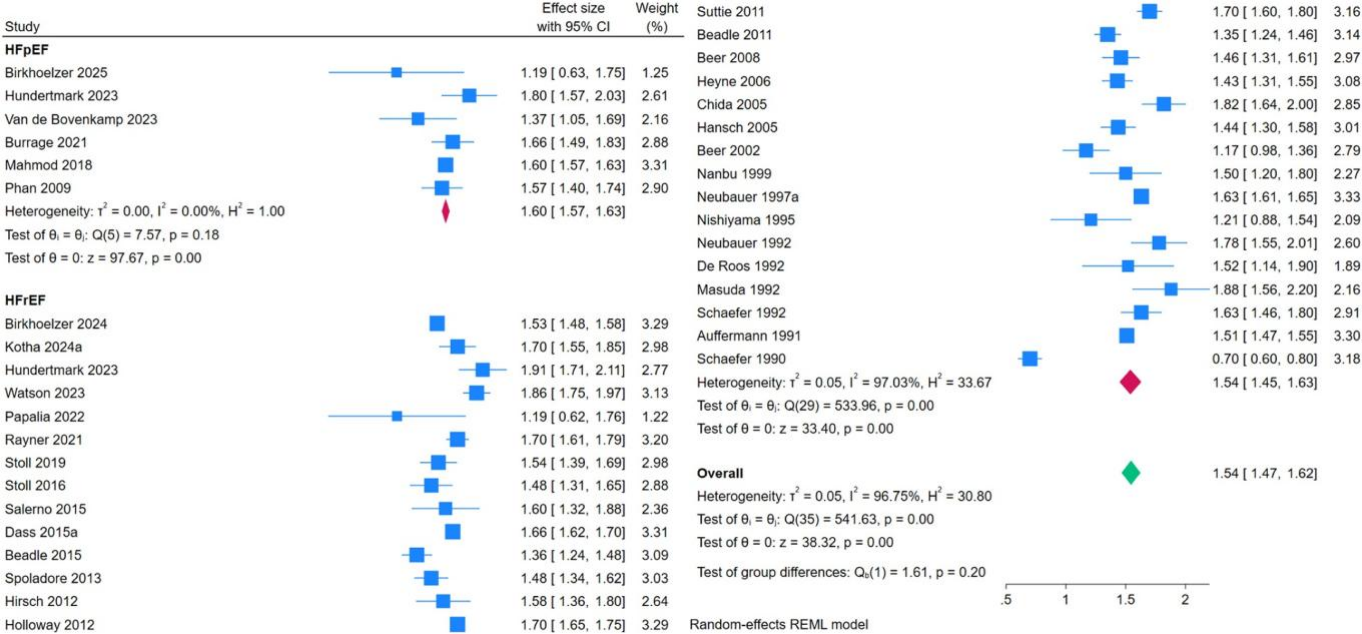
Forest plot of PCr/ATP ratio for heart failure (HFrEF and HFpEF).

Thirty and six studies reported PCr/ATP ratios in individuals with HFrEF (575 participants) and HFpEF (146 participants), respectively (*Figure 2*). Participants with HFrEF were 71% male and had a mean age of 57 years, while HFpEF participants were 46% male with a mean age of 70 years. Both groups had similar PCr/ATP ratio overall estimates (HFrEF: 1.54, 1.45–1.63; HFpEF: 1.60, 1.57–1.63), and there was no significant difference between these two groups (*Figure 4*). Adjustment for sequence and year of publication slightly increased the PCr/ATP ratio for HFrEF to 1.56 (95% CI 1.44–1.66, *Figure 2*). Adjustment successfully reduced heterogeneity from low to very low levels (Tau^2^ 0.05–0.01).

### Valve disease

**Figure 5:**
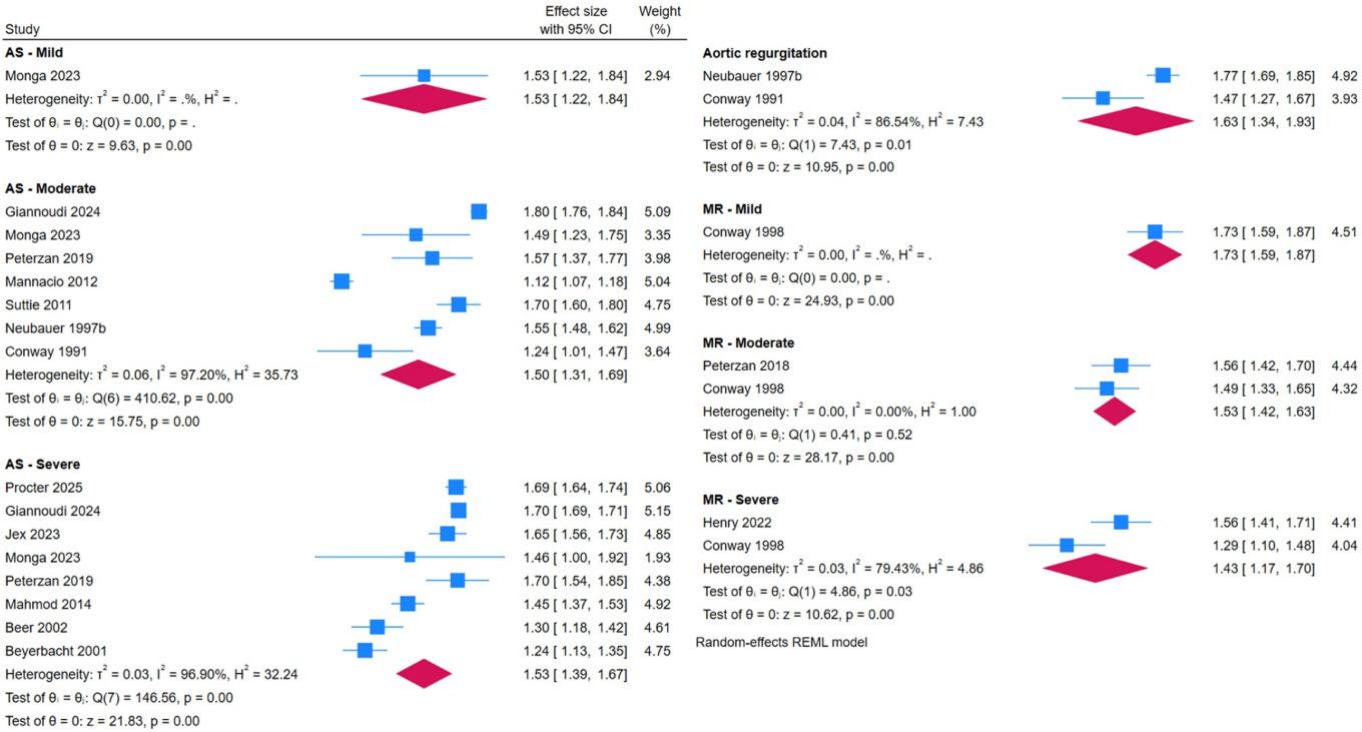
Forest plot of PCr/ATP ratio for valve disease.

Aortic stenosis (AS) was the commonest valve disease investigated for cardiac energetics (12 studies, 613 participants, *Figure 2*). Mean age of participants was 73 years with 65% male. With one, seven, and eight studies reporting mild, moderate, and severe AS, respectively. These three groups yielded similar PCr/ATP overall estimates of: mild—1.53 (95% CI 1.22–1.84); moderate—1.50 (95% CI 1.31–1.69); and severe—1.53 (95% CI 1.39–1.67) (*Figure 5*). There was no evidence of difference between AS severities, and the overall estimate for AS collectively was 1.52 (1.42–1.62; Q_b_ 0.07, *P* > 0.05) and 1.51 (95% CI 1.43–1.59) with adjustment for field strength and year of publication (*Figure 2*). Adjustment successfully reduced heterogeneity from low to very low levels (Tau^2^ 0.04–0.02).

Two studies, 17 participants, reported PCr/ATP ratios in participants with aortic regurgitation. These studies yielded an overall estimate of 1.63 (95% CI 1.34–1.93; *Figures 2* and *5*).

Mitral regurgitation (MR) was reported by a small number of studies (three studies), 59 participants, yielding overall estimates of mild—1.63 (95% CI 1.59–1.87); moderate—1.53 (95% CI 1.42–1.63); and severe—1.43 (95% CI 1.17–1.70; *Figures 2* and *5*). There was a significant difference between these groups with a trend for lower PCr/ATP ratios with more severe disease.

### Diabetes

**Figure 6:**
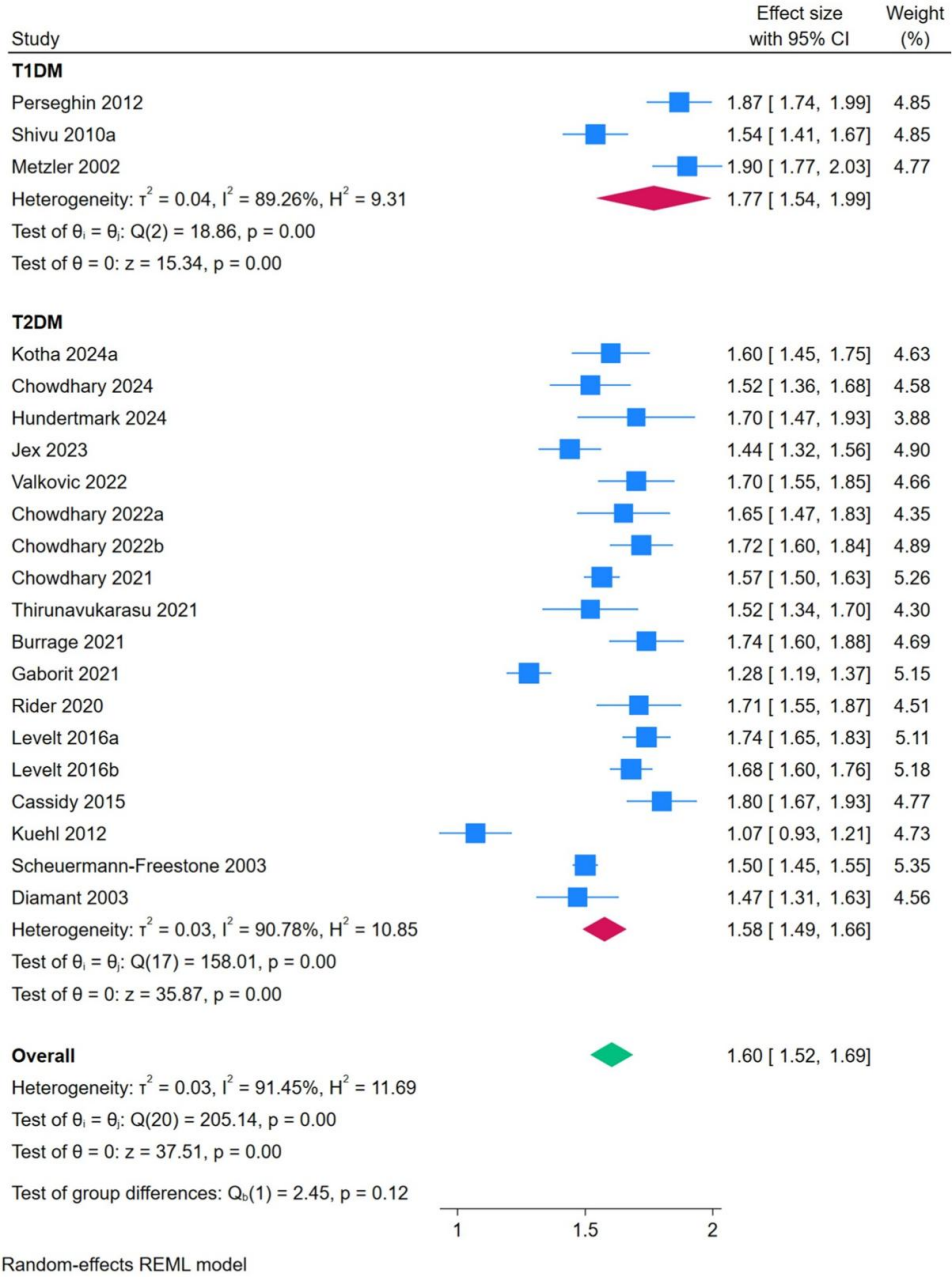
Forest plot of PCr/ATP ratio for diabetes .

PCr/ATP ratios were reported in 18 studies for T2DM (496 participants) and three for type 1 diabetes mellitus (T1DM) (98 participants). Type 2 diabetes mellitus (T2DM) participants were 62% male with an average age of 60 years, and T1DM participants were 67% male with an average age of 37 years. The overall PCr/ATP ratio estimates for each group were T2DM—1.58 (1.49–1.66) and T1DM—1.77 (1.54–1.99; *Figure 6*). There was no significant difference between both groups. Adjustment for field strength and year of publication reduced PCr/ATP ratio slightly to 1.57 (1.47–1.69) and successfully reduced heterogeneity from low to very low levels (Tau^2^ 0.03–0.00).

### Hypertrophic cardiomyopathy

**Figure 7:**
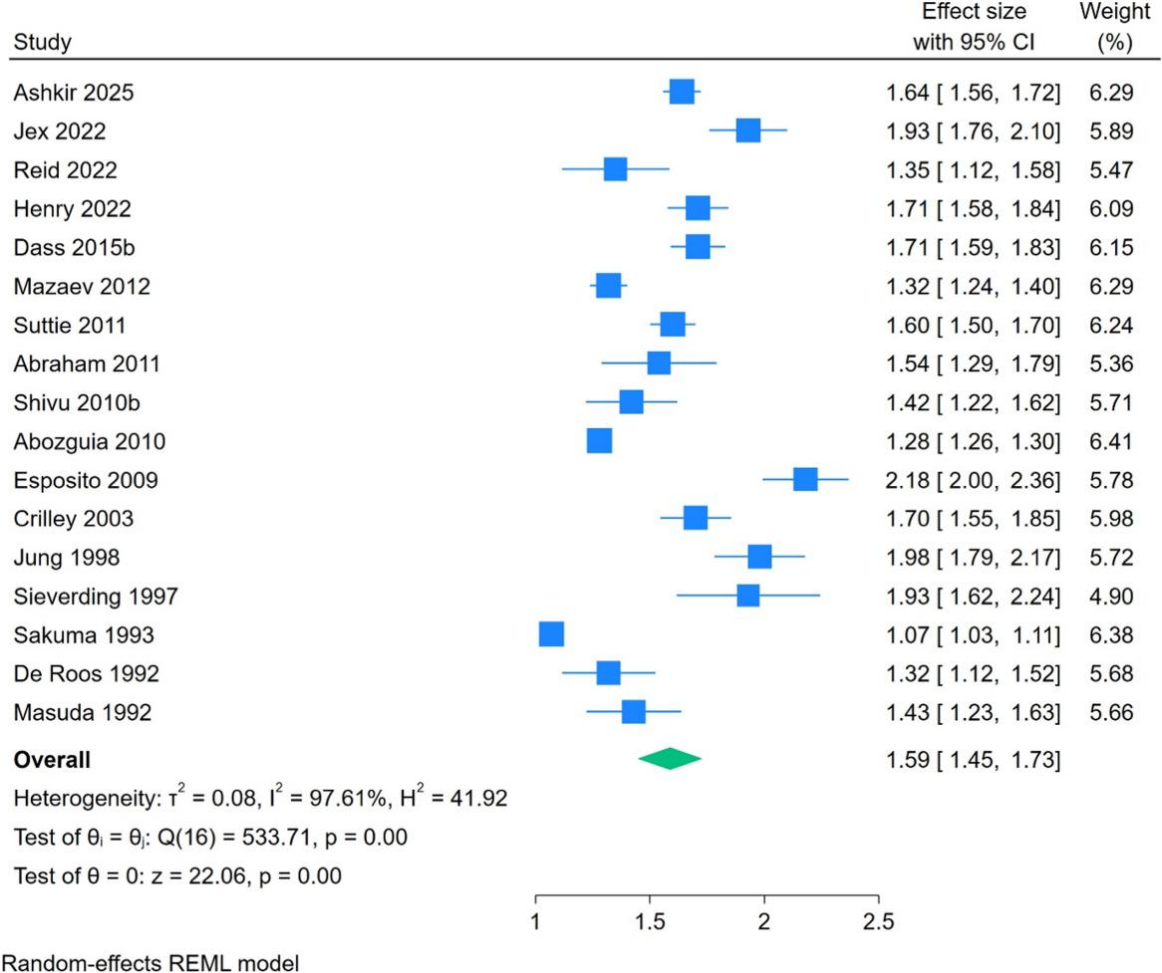
Forest plot of PCr/ATP ratio in HCM.

Seventeen studies with 424 participants (aged 47.9 years, 71.5% male) reported PCr/ATP ratio in people with hypertrophic cardiomyopathy (HCM), and analysis yielded an overall PCr/ATP estimate of 1.59 (1.45–1.73, *Figure 7*) and 1.56 (1.34–1.79, *Figure 2*) when adjusted for field strength and year of publication. Adjustment was not able to substantially reduce heterogeneity (Tau2 0.08–0.05) suggesting that there is residual heterogeneity in this estimate. There was evidence of small study bias whereby smaller studies tended to report greater PCr/ATP ratios (Egger’s regression 3.54, SE 1.61, *P* = 0.03, Supplementary data online, *Figure S6H*).

### Methodological factors influencing PCr/ATP ratio in healthy volunteers

Ninety-four studies (1951 participants) with data for healthy volunteers also reported the CMR sequence used. There was a significant difference between chemical shift imaging (CSI), image selected *in vivo* spectroscopy (ISIS) and depth resolved surface coil (DRESS) sequences, whereby CSI was greater than ISIS which was greater than DRESS (see Supplementary data online, *Figure S2*). Field strength was reported by 132 studies with healthy volunteer data (2896 participants), and there was a significant positive correlation between PCr/ATP ratio and field strength (see Supplementary data online, *Figure S3A*). However, in subgroup meta-analysis of 1.5T, 2T, 3T, and 7T, 1.5T was significantly lower than ≥ 2T (*P* = 0.005), and there was no difference between 2T vs. 3T vs. 7T (*P* > 0.05). Forty studies (744 participants) reported repetition time (TR), and there was no significant correlation between PCr/ATP ratio and TR (see Supplementary data online, *Figure S3B*), suggesting that studies satisfactorily corrected for saturation. Seventy-five studies with healthy volunteers (1527 participants) reported voxel location, and there was a significant difference between myocardial tissues with septum giving a higher PCr/ATP ratio than anterior ventricle and apex (see Supplementary data online, *Figure S4*). Ninety-two studies with healthy volunteers (2029 participants) reported participant position (supine or prone), but there was no evidence that this affected the pooled PCr/ATP ratio (see Supplementary data online, *Figure S5*).

### Risk of bias

Seventy-eight (44%) studies were considered low risk for the purposes of obtaining a pooled population average (see Supplementary data online, *Table S5*). Seventy-six (43%) studies were considered moderate risk, predominantly for small sample sizes, limited methodological information, and unclear risk of generalizability. Twenty (11%) studies were considered high risk predominantly for being small, feasibility studies. Sixty studies were found to potentially be at risk of small study bias.

We found that using GPT5 LLM had a good inter-reviewer reliability of Cohen’s κ 0.808 (± SE 0.041), similar to previously reported.^12^

### Sensitivity analysis

Supplementary data online, *Table S6* shows our sensitivity analysis. We found significant residual heterogeneity in the assessment of the pooled average PCr/ATP ratio for individuals with HCM. As such, the fixed-effects analysis for HCM was significantly lower than the random-effects analysis. Otherwise, removal of studies at medium or high risk of bias for a population average did not affect our overall estimates. Fixed-effects analysis is probably not appropriate for these large, relatively heterogenous populations. Limiting analysis to full texts also did not affect overall estimates other than reducing precision of estimates.

## Discussion

This study systematically compiles published cardiac energetic data and provides pooled average estimates for healthy and disease states, whilst also providing a compendium of published data. We show that pooled PCr/ATP ratio averages vary with methodological factors including field strength, sequence, and voxel location, but not position (supine or prone). Moreover, we show that at the study level BMI and natriuretic peptides (BNP and NT-proBNP) are negatively correlated with PCr/ATP and year of publication and LVEF are positively correlated with PCr/ATP ratio, whereas age and sex are not correlated.

### Energetic derangements as a final common pathway

Our findings support the concept that energetic impairment represents a final common pathway across cardiovascular disease. Reductions in PCr/ATP were observed consistently in HFrEF, HFpEF, valvular heart disease, diabetes, and hypertrophic cardiomyopathy, suggesting that derangements in high-energy phosphate handling are a unifying hallmark of myocardial dysfunction. Importantly, these impairments correlated with markers of disease severity, including elevated natriuretic peptides and lower left ventricular ejection fraction, and prior work has demonstrated that reduced PCr/ATP is an independent predictor of mortality.^8^ Future studies should further explore the relationship with PCr/ATP and clinical outcomes in the era of improved diagnostics and treatments.

### Myocardial energetics as a therapeutic target

The observation that energetic impairment is present across diverse cardiac and systemic conditions highlights myocardial metabolism as an attractive therapeutic target. Pharmacological interventions aimed at augmenting myocardial energy supply have shown promise in early-phase studies. For instance, the metabolic modulator perhexiline improved myocardial energetics and symptoms in HFrEF,^14^ while provision of ketone bodies has been shown to boost PCr/ATP in individuals with type 2 diabetes.^15^ More recently, ninerefaxstat, a novel metabolic agent, demonstrated improvements in cardiac energetics in both T2DM^16^ and HFpEF^17^ populations. Together, these data suggest that therapeutic restoration of myocardial energy reserve could represent a novel disease-modifying strategy across the spectrum of cardiometabolic disease.

### Cardiometabolic disease and weight loss

Cardiometabolic conditions appear to exert particularly adverse effects on myocardial energetics. BMI correlated negatively with PCr/ATP in our analysis, and diabetes was consistently associated with reduced energetic reserve. Importantly, studies have shown that weight loss in obesity improves PCr/ATP,^5,18^ underscoring the potential reversibility of energetic derangements. Interestingly, liraglutide improved myocardial energetics despite minimal weight loss (<2 kg),^19^ suggesting that certain pharmacological interventions may exert direct myocardial metabolic benefits independent of weight reduction. Whether contemporary GLP-1 receptor agonists such as semaglutide, or dual/triple incretin agonists, such as tirzepatide or retatrutide, or the developing oral GLP-1 therapies, further augment myocardial energetics beyond weight loss remains unknown and warrants investigation. Moreover, whether intentional weight loss improves PCr/ATP in other cardiac conditions, such as valvular disease, heart failure, or hypertrophic cardiomyopathy with concomitant obesity also remains unexplored.

### Methodological factors

We observed that year of study publication was positively correlated with PCr/ATP ratio in healthy volunteers. This likely reflects advances in acquisition and post-processing techniques, particularly the improved application of blood-pool correction, which is known to lower measured PCr/ATP. By contrast, age and sex were not significantly associated with PCr/ATP in our pooled analyses, though individual patient-level studies may be required to fully elucidate their contribution. The use of study averages in meta-analysis may mask subtle demographic effects, emphasizing the need for future pooled patient-level analyses. Moreover, women were under-represented in the included healthy volunteer studies, comprising only around one-third of participants, which may have limited power to detect sex-related differences.

Pooled PCr/ATP ratio estimates are not changed substantially by adjustment, suggesting that at scale, methodological variations make little difference to average PCr/ATP ratio. However, adjustment appears to be important when assessing for correlation. Importantly, the minimal impact of adjustment for multiple methodological factors on overall estimates supports the continued evaluation of PCr/ATP in multicentre settings, but standardization of acquisition and analysis methods will remain important before broad cross-centre comparability can be assumed.

### Limitations

Non-probability sampling is likely to be more widespread than we identified in our risk of bias assessment. However, this is unlikely to change our overall estimates. The meta-regression analyses used study-level rather than individual participant-level data and are therefore subject to ecological bias, limiting inference about individual-level biological relationships. It was generally not possible to adjust for voxel location given the large number of subgroups required for accurate description and the risk of collinearity when including <8 studies per meta-regressed variable. Moreover, it is possible that there are other unmeasurable factors that cannot be used in meta-regression. However, in most models, we use the maximum number of associated variables and achieve reductions in heterogeneity as measured by Tau^2^ throughout. There was residual heterogeneity following adjustment in the estimate of average PCr/ATP ratio for HCM, our sensitivity analysis found a significant reduction in the fixed-effects estimate for PCr/ATP for HCM, and there was evidence of small study bias and so this analysis should be treated with some caution.

Several subgroup comparisons should be interpreted cautiously because the available evidence was limited, particularly for HFpEF, T1DM, aortic regurgitation, and MR. In these settings, an absence of statistically significant difference may reflect uncertainty related to the small numbers of studies and participants, rather than true equivalence.

## Conclusion

This comprehensive systematic review and meta-analysis provides pooled PCr/ATP estimates across health and disease, synthesizing nearly four decades of cardiac energetics research. Our findings demonstrate that impaired cardiac energetics, reflected by reduced PCr/ATP ratios, represents a common metabolic pathway across diverse cardiac pathologies, with similar energetic impairment observed in HFrEF and HFpEF. Whilst methodological factors including field strength, sequence type, and voxel location influence individual measurements, their minimal impact on pooled estimates supports the validity of cross-study comparisons. These pooled estimates provide benchmarks for interpreting individual patient data, designing adequately powered clinical trials, and evaluating therapeutic interventions targeting cardiac metabolism.

## Supplementary Material

qyag110_Supplementary_Data

## Data Availability

This meta-analysis used data extracted from previously published studies, which are cited in the reference list. All data generated through the analysis, including pooled estimates, summary tables, and forest plots, are included in the article and its supplementary materials. The dataset of extracted study-level data can be made available from the corresponding author upon reasonable request.

## Lead author biography

Jonathan J.H. Bray is an NIHR Academic Clinical Fellow in Cardiology at University Hospitals Bristol, Cardiology Registrar at The Great Western Hospital and an Honorary Clinical Research Fellow at University College London. His research interests include meta-analysis, cardiovascular epidemiology, and trials.

John A. Henry is an NIHR Academic Clinical Fellow in Cardiology at Oxford University Hospitals NHS Foundation Trust and a Clinical Research Fellow at the Oxford Centre for Clinical Magnetic Resonance Research (OCMR). He is also an Honorary Research Fellow at Jersey General Hospital. His research interests include multimodality cardiovascular imaging with a particular focus on obesity, cardiometabolic disease, and heart failure.

## References

[1] Neubauer S . The failing heart — an engine out of fuel. N Engl J Med 2007;356:1140–51. 17360992 10.1056/NEJMra063052

[2] Neubauer S, Krahe T, Schindler R, Horn M, Hillenbrand H, Entzeroth C et al 31P magnetic resonance spectroscopy in dilated cardiomyopathy and coronary artery disease. Altered cardiac high-energy phosphate metabolism in heart failure. Circulation 1992;86:1810–8. 1451253 10.1161/01.cir.86.6.1810

[3] Burrage MK, Hundertmark M, Valkovič L, Watson WD, Rayner J, Sabharwal N et al Energetic basis for exercise-induced pulmonary congestion in heart failure with preserved ejection fraction. Circulation 2021;144:1664–78. 34743560 10.1161/CIRCULATIONAHA.121.054858 PMC8601674

[4] Henry JA, Levelt E, Rayner JJ, Hundertmark MJ, Peterzan MA, Green PG et al Investigating myocardial energetic deficit across the spectrum of cardiac disease. Eur Heart J 2022;43:ehac544.244, 10.1093/eurheartj/ehac544.244

[5] Rayner JJ, Peterzan MA, Watson WD, Clarke WT, Neubauer S, Rodgers CT et al Myocardial energetics in obesity. Circulation 2020;141:1152–63. 32138541 10.1161/CIRCULATIONAHA.119.042770 PMC7144750

[6] Lamb HJ, Beyerbacht HP, van der Laarse A, Stoel BC, Doornbos J, van der Wall EE et al Diastolic dysfunction in hypertensive heart disease is associated with altered myocardial metabolism. Circulation 1999;99:2261–7. 10226091 10.1161/01.cir.99.17.2261

[7] Levelt E, Mahmod M, Piechnik SK, Ariga R, Francis JM, Rodgers CT et al Relationship between left ventricular structural and metabolic remodeling in type 2 diabetes. Diabetes 2016;65:44–52. 26438611 10.2337/db15-0627 PMC4890658

[8] Neubauer S, Horn M, Cramer M, Harre K, Newell JB, Peters W et al Myocardial phosphocreatine-to-ATP ratio is a predictor of mortality in patients with dilated cardiomyopathy. Circulation 1997;96:2190–6. 9337189 10.1161/01.cir.96.7.2190

[9] Abozguia K, Elliott P, McKenna W, Phan TT, Nallur-Shivu G, Ahmed I et al Metabolic modulator perhexiline corrects energy deficiency and improves exercise capacity in symptomatic hypertrophic cardiomyopathy. Circulation 2010;122:1562–9. 20921440 10.1161/CIRCULATIONAHA.109.934059

[10] van de Bovenkamp AA, Geurkink KTJ, Oosterveer FTP, de Man FS, Kok WEM, Bronzwaer PNA et al Trimetazidine in heart failure with preserved ejection fraction: a randomized controlled cross-over trial. ESC Heart Fail 2023;10:2998–3010. 37530098 10.1002/ehf2.14418 PMC10567667

[11] Cooper H, Hedges LV, & Valentine JC. The Handbook of Research Synthesis and Meta-Analysis. 2nd edition. USA: Russell Sage Foundation; 2009.

[12] Lai H, Ge L, Sun M, Pan B, Huang J, Hou L et al Assessing the risk of bias in randomized clinical trials with large language models. JAMA Netw Open 2024;7:e2412687–2412687. 38776081 10.1001/jamanetworkopen.2024.12687 PMC11112444

[13] Hoy D, Brooks P, Woolf A, Blyth F, March L, Bain C et al Assessing risk of bias in prevalence studies: modification of an existing tool and evidence of interrater agreement. J Clin Epidemiol 2012;65:934–9. 22742910 10.1016/j.jclinepi.2011.11.014

[14] Beadle RM, Williams LK, Kuehl M, Bowater S, Abozguia K, Leyva F et al Improvement in cardiac energetics by perhexiline in heart failure due to dilated cardiomyopathy. JACC Heart Fail 2015;3:202–11. 25650370 10.1016/j.jchf.2014.09.009

[15] Kotha S, Procter H, Giannoudi M, Plein S, Xue H, Valkovic L et al Effects of exogenous ketone supplementation on cardiac energetics, steatosis, function, and perfusion in type 2 diabetes, heart failure, and healthy participants- a single center, open labelled clinical study. Heart 2024;110:A145EP—A147.

[16] Hundertmark MJ, Birkhoelzer SM, Portwood C, Siu AG, Matthews V, Lewis AJ et al IMPROVE-DiCE, a 2-part, open-label, phase 2a trial evaluating the safety and effectiveness of ninerafaxstat in patients with cardiometabolic syndromes. Circulation 2026;153:550—563. 41410037 10.1161/CIRCULATIONAHA.125.074041 PMC12928817

[17] Birkhoelzer S, Portwood C, Pelado J, Mills R, Yavari A, Patel J et al Effects of ninerafaxstat on myocardial energetics, exercise capacity, and cardiac function in heart failure with preserved ejection fraction, type 2 diabetes and obesity- a phase 2A clinical trial. JACC 2025;85:1164–.

[18] Rider OJ, Francis JM, Tyler D, Byrne J, Clarke K, Neubauer S. Effects of weight loss on myocardial energetics and diastolic function in obesity. Int J Cardiovasc Imaging 2013;29:1043–50. 23269470 10.1007/s10554-012-0174-6

[19] Chowdhary A, Thirunavukarasu S, Joseph T, Jex N, Kotha S, Giannoudi M et al Liraglutide improves myocardial perfusion and energetics and exercise tolerance in patients with type 2 diabetes. J Am Coll Cardiol 2024;84:540–57. 39084829 10.1016/j.jacc.2024.04.064 PMC11296502

