# Supplementary material for: Cardiac energetics in health and disease: a meta-analysis
Source: Eur Heart J Imaging Methods Pract. 2026 Jun 17;4(3):qyag110. doi: 10.1093/ehjimp/qyag110 (PMC13344855; doi:10.1093/ehjimp/qyag110)
Supplement: qyag110_Supplementary_Data [file qyag110_supplementary_data.docx]

**Supplemental Material**

**Supplementary Table 1** – Search strategy

Ovid MEDLINE(R) ALL <1946 to August 01, 2025>

1 PCr:ATP.mp. 13

2 (phosphocreatine adj5 ATP).mp. [mp=title, book title, abstract, original title, name of substance word, subject heading word, floating sub-heading word, keyword heading word, organism supplementary concept word, protocol supplementary concept word, rare disease supplementary concept word, unique identifier, synonyms, population supplementary concept word, anatomy supplementary concept word] 2045

3 (phosphocreatine adj5 adenosine triphosphate).mp. [mp=title, book title, abstract, original title, name of substance word, subject heading word, floating sub-heading word, keyword heading word, organism supplementary concept word, protocol supplementary concept word, rare disease supplementary concept word, unique identifier, synonyms, population supplementary concept word, anatomy supplementary concept word] 2536

4 (phosphocreatine adj5 adenosine-5'-triphosphate).mp. [mp=title, book title, abstract, original title, name of substance word, subject heading word, floating sub-heading word, keyword heading word, organism supplementary concept word, protocol supplementary concept word, rare disease supplementary concept word, unique identifier, synonyms, population supplementary concept word, anatomy supplementary concept word] 22

5 (phosphocreatine adj5 triphosphate adenosine).mp. [mp=title, book title, abstract, original title, name of substance word, subject heading word, floating sub-heading word, keyword heading word, organism supplementary concept word, protocol supplementary concept word, rare disease supplementary concept word, unique identifier, synonyms, population supplementary concept word, anatomy supplementary concept word] 4

6 (PCr adj5 ATP).mp. [mp=title, book title, abstract, original title, name of substance word, subject heading word, floating sub-heading word, keyword heading word, organism supplementary concept word, protocol supplementary concept word, rare disease supplementary concept word, unique identifier, synonyms, population supplementary concept word, anatomy supplementary concept word] 1722

7 (Creatine phosphate adj5 ATP).mp. [mp=title, book title, abstract, original title, name of substance word, subject heading word, floating sub-heading word, keyword heading word, organism supplementary concept word, protocol supplementary concept word, rare disease supplementary concept word, unique identifier, synonyms, population supplementary concept word, anatomy supplementary concept word] 982

8 phosphocreatine?ATP.mp. [mp=title, book title, abstract, original title, name of substance word, subject heading word, floating sub-heading word, keyword heading word, organism supplementary concept word, protocol supplementary concept word, rare disease supplementary concept word, unique identifier, synonyms, population supplementary concept word, anatomy supplementary concept word] 7

9 phosphocreatine???ATP.mp. [mp=title, book title, abstract, original title, name of substance word, subject heading word, floating sub-heading word, keyword heading word, organism supplementary concept word, protocol supplementary concept word, rare disease supplementary concept word, unique identifier, synonyms, population supplementary concept word, anatomy supplementary concept word] 7

10 PCr?ATP.mp. [mp=title, book title, abstract, original title, name of substance word, subject heading word, floating sub-heading word, keyword heading word, organism supplementary concept word, protocol supplementary concept word, rare disease supplementary concept word, unique identifier, synonyms, population supplementary concept word, anatomy supplementary concept word] 13

11 exp heart/ 577558

12 cardiac.mp. 967379

13 myocard*.mp. 668396

14 exp cardiac muscle/ 213347

15 1 or 2 or 3 or 4 or 5 or 6 or 7 or 8 or 9 or 10 5484

16 11 or 12 or 13 or 14 1533361

17 15 and 16 2186

18 limit 17 to humans 456

Embase <1974 to 2025 August 01>

1 PCr:ATP.mp. 1

2 (phosphocreatine adj5 ATP).mp. [mp=title, abstract, heading word, drug trade name, original title, device manufacturer, drug manufacturer, device trade name, keyword heading word, floating subheading word, candidate term word] 2379

3 (phosphocreatine adj5 adenosine triphosphate).mp. [mp=title, abstract, heading word, drug trade name, original title, device manufacturer, drug manufacturer, device trade name, keyword heading word, floating subheading word, candidate term word] 699

4 (phosphocreatine adj5 adenosine-5'-triphosphate).mp. [mp=title, abstract, heading word, drug trade name, original title, device manufacturer, drug manufacturer, device trade name, keyword heading word, floating subheading word, candidate term word] 24

5 (phosphocreatine adj5 triphosphate adenosine).mp. [mp=title, abstract, heading word, drug trade name, original title, device manufacturer, drug manufacturer, device trade name, keyword heading word, floating subheading word, candidate term word] 6

6 (PCr adj5 ATP).mp. [mp=title, abstract, heading word, drug trade name, original title, device manufacturer, drug manufacturer, device trade name, keyword heading word, floating subheading word, candidate term word] 2270

7 (Creatine phosphate adj5 ATP).mp. [mp=title, abstract, heading word, drug trade name, original title, device manufacturer, drug manufacturer, device trade name, keyword heading word, floating subheading word, candidate term word] 1054

8 phosphocreatine?ATP.mp. [mp=title, abstract, heading word, drug trade name, original title, device manufacturer, drug manufacturer, device trade name, keyword heading word, floating subheading word, candidate term word] 0

9 phosphocreatine???ATP.mp. [mp=title, abstract, heading word, drug trade name, original title, device manufacturer, drug manufacturer, device trade name, keyword heading word, floating subheading word, candidate term word] 0

10 PCr?ATP.mp. [mp=title, abstract, heading word, drug trade name, original title, device manufacturer, drug manufacturer, device trade name, keyword heading word, floating subheading word, candidate term word] 1

11 exp phosphorus nuclear magnetic resonance/ 11303

12 exp heart/ 937369

13 cardiac.mp. [mp=title, abstract, heading word, drug trade name, original title, device manufacturer, drug manufacturer, device trade name, keyword heading word, floating subheading word, candidate term word] 1392768

14 myocard*.mp. [mp=title, abstract, heading word, drug trade name, original title, device manufacturer, drug manufacturer, device trade name, keyword heading word, floating subheading word, candidate term word] 742288

15 exp cardiac imaging/ 18640

16 exp cardiac muscle/ 248650

17 1 or 2 or 3 or 4 or 5 or 6 or 7 or 8 or 9 or 10 or 11 15276

18 12 or 13 or 14 or 15 or 16 2178718

19 17 and 18 2574

20 limit 19 to human 833

Search Name: PCrATP meta.

Date Run: 01/08/2025 07:51:14

Comment:

ID Search Hits

#1 PCr*ATP 53

#2 phosphocreatine?ATP 3

#3 phosphocreatine???ATP 3

#4 phosphocreatine NEXT/5 ATP 40

#5 phosphocreatine NEXT/5 adenosine triphosphate 30

#6 phosphocreatine NEXT/5 triphosphate adenosine 27

#7 PCr NEXT/5 ATP 80

#8 Creatine phosphate NEXT/5 ATP 7

#9 MeSH descriptor: [Nuclear Magnetic Resonance, Biomolecular] explode all trees 24

#10 #1 OR #2 OR #3 OR #4 OR #5 OR #6 OR #7 OR #8 OR #9 141

#11 MeSH descriptor: [Heart] explode all trees 9543

#12 Cardiac 91089

#13 myocard* 57099

#14 MeSH descriptor: [Cardiac Imaging Techniques] explode all trees 11447

#15 MeSH descriptor: [Myocardium] explode all trees 1703

#16 #11 OR #12 OR #13 OR #14 OR #15 130684

#17 #10 AND #16 67

**Supplementary Table 2** - All included studies

| **Study #** | **Study** | **Populations included** | **Study #** | **Study** | **Populations included** |
| --- | --- | --- | --- | --- | --- |
| 1 | Procter 2025^1^ | HVs, AS | 89 | Perseghin 2012^2^ | HVs, T1DM |
| 2 | Ashkir 2025^3^ | HVs, HCM | 90 | Mannacio 2012^4^ | AS |
| 3 | Birkhoelzer 2025^5^ | HFpEF | 91 | Kuehl 2012^6^ | HVs, T2DM |
| 4 | de Wit-Verheggen 2025^7^ | Pre-diabetes, overweight | 92 | Patel 2011^8^ | HTN, CKD |
| 5 | Giannoudi 2025^9^ | HVs | 93 | Holloway 2011a^10^ | HVs, Altitude |
| 6 | Karkouri 2025^11^ | HVs | 94 | Holloway 2011b^12^ | HVs |
| 7 | Soo 2025b^13^ | HVs, RA | 95 | Suttie 2011^14^ | HVs, AS, HFrEF, HCM |
| 8 | Soo 2025a^15^ | HVs, SS | 96 | Beadle 2011^16^ | HVs, HFrEF |
| 9 | Burchert 2025^17^ | HVs, pre-term adults | 97 | Abraham 2011^18^ | HVs, HCM |
| 10 | Thirunavukarasu 2025^19^ | Pregnancy, gestatational DM, pre-eclampsia | 98 | Fragasso 2011^20^ | HVs |
| 11 | Birkhoelzer 2024^21^ | HFrEF | 99 | Jones 2010^22^ | HVs, PBC |
| 12 | Giannoudi 2024^23^ | HVs, AS | 100 | Shivu 2010a^24^ | HVs, T1DM |
| 13 | Kotha 2024a^25^ | HVs, T2DM, HFrEF | 101 | Shivua 2010b^26^ | HVs, HCM |
| 14 | Kotha 2024b^27^ | IHD, T2DM | 102 | Abozguia 2010^28^ | HVs, HCM |
| 15 | Kotha 2024c^29^ | PR | 103 | Holloway 2010^30^ | HVs |
| 16 | Ng 2024^31^ | Dialysis | 104 | Hollingsworth 2010^32^ | CFS |
| 17 | Chowdhary 2024^33^ | T2DM | 105 | Hudsmith 2009^34^ | HVs |
| 18 | Hundertmark 2024^35^ | T2DM | 106 | Burkhard 2009^36^ | Hypertension |
| 19 | Chamley 2024^37^ | HVs, prior Covid | 107 | Phan 2009^38^ | HVs, HFpEF |
| 20 | Hundertmark 2023^39^ | HFrEF, HFpEF | 108 | Esposito 2009^40^ | HVs, HCM |
| 21 | Jex 2023^41^ | HVs, T2DM, AS | 109 | Bottomley 2009^42^ | HVs, IHD |
| 22 | Goldenberg 2023^43^ | HFimpEF | 110 | Tyler 2009^44^ | HVs |
| 23 | Watson 2023^45^ | HVs, HFrEF | 111 | Beer 2008^46^ | HVs, HFrEF |
| 24 | Monga 2023^47^ | HVs, AS | 112 | Perseghin 2008^48^ | HVs, NAFLD |
| 25 | Van de Bovenkamp 2023^49^ | HFpEF | 113 | Wolf 2008^50^ | HVs, Friedrich’s ataxia |
| 26 | Gamble 2023^51^ | HVs, post-anthracyclines | 114 | Klug 2007^52^ | HVs |
| 27 | Papalia 2022^53^ | HVs, HFrEF | 115 | Perseghin 2007^54^ | HVs, obesity |
| 28 | Gorecka 2022^55^ | HVs, Long Covid | 116 | Beer 2007^56^ | HVs, IHD |
| 29 | Jex 2022^57^ | HVs, HCM, T2DM | 117 | Van Der Meer 2007^58^ | HVs |
| 30 | Reid 2022^59^ | HCM | 118 | Heyne 2006^60^ | HVs, HFrEF, Hypertension |
| 31 | Cameron 2022^61^ | HVs | 119 | Caus 2006^62^ | HVs, heart transplant |
| 32 | Valkovic 2022^63^ | HVs, T2DM | 120 | Perseghin 2005^64^ | HVs, kidney & SPK transplants |
| 33 | Chowdhary 2022a^65^ | T2DM | 121 | Najjar 2005^66^ | IHD |
| 34 | Chowdhary 2022b^67^ | HVs, Athletes, T2DM | 122 | Chida 2005^68^ | HFrEF |
| 35 | Henry 2022^69^ | MR | 123 | Hansch 2005^70^ | HVs, HFrEF |
| 36 | Wibowo 2021^71^ | HVs | 124 | Schocke 2004^72^ | HVs, haemochromatosis |
| 37 | Chowdhary 2021^73^ | HVs, T2DM, overweight | 125 | Beer 2004^74^ | IHD |
| 38 | Thirunavukarasu 2021^75^ | HVs, T2DM | 126 | Lodi 2004^76^ | HVs, mitochondrial disease |
| 39 | Burrage 2021^77^ | HVs, T2DM, HFpEF, Amyloid | 127 | Schocke 2003a^78^ | HVs, familial hypercholesterolaemia |
| 40 | Gaborit 2021^79^ | T2DM | 128 | Schocke 2003b^80^ | HVs |
| 41 | Watson 2021^81^ | HVs | 129 | Scheuermann-Freestone 2003^82^ | HVs, T2DM |
| 42 | Rayner 2021^83^ | HVs, HFrEF | 130 | Ogimoto 2003^84^ | HVs, dialysis |
| 43 | Apps 2021^85^ | HVs | 131 | Diamant 2003^86^ | HVs, T2DM |
| 44 | Lewis 2021^87^ | HVs, obesity | 132 | Crilley 2003^88^ | HVs, HCM |
| 45 | Rayner 2020^89^ | HVs, obesity | 133 | Spindler 2003^90^ | MS |
| 46 | Rider 2020^91^ | HVs, T2DM | 134 | Beer 2002a^92^ | HVs, hypertension, AS, HFrEF |
| 47 | Peterzan 2020^93^ | AS, HFrEF, HVs | 135 | Beer 2002b^94^ | Myotonic dystrophy |
| 48 | Ellis 2019^95^ | HVs | 136 | Metzler 2002^96^ | HVs, T1DM |
| 49 | De Cobelli 2019^97^ | HVs, GH deficiency | 137 | Pohmann 2001^98^ | HVs |
| 50 | Stoll 2019^99^ | HVs, HFrEF | 138 | Beyerbacht 2001^100^ | HVs, AS |
| 51 | Nathania 2018^101^ | HVs | 139 | Lodi 2001^102^ | HVs, Friedrich’s ataxia |
| 52 | Mahmod 2018^103^ | HVs, HFpEF | 140 | Moka 2001^104^ | HVs, IHD |
| 53 | Scally 2018^105^ | HVs, Takotsubo | 141 | Beer 2000^106^ | HVs, IHD |
| 54 | Peterzan 2018^107^ | HVs, MR | 142 | Crilley 2000^108^ | BMD |
| 55 | Rayner 2018^109^ | HVs, obesity | 143 | Eidenschink 2000^110^ | HVs, post-anthracyclines |
| 56 | Valkovic 2017^111^ | HVs | 144 | Buchthal 2000^112^ | HVs, heart transplant |
| 57 | Stoll 2016^113^ | HVs, HFrEF | 145 | Pluim 1999^114^ | HVs, athletes |
| 58 | Bowater 2016^115^ | HVs, Eisenmenger syndrome | 146 | Lamb 1999^116^ | HVs, hypertension |
| 59 | Levelt 2016a^117^ | HVs, T2DM | 147 | Nanbu 1999^118^ | HVs, HFrEF |
| 60 | Levelt 2016b^119^ | HVs, T2DM | 148 | Jung 1998^120^ | HVs, HCM |
| 61 | Wijesurendra 2016^121^ | AF | 149 | Tagami 1998^122^ | HVs, dialysis |
| 62 | Salerno 2015^123^ | HFrEF | 150 | Pluim 1998^124^ | HVs, athletes |
| 63 | Dass 2015a^125^ | HVs, HFrEF | 151 | Loffler 1998^126^ | HVs |
| 64 | Dass 2015b^127^ | HVs, HCM | 152 | Conway 1998^128^ | HVs, MR |
| 65 | Ntusi 2015^129^ | HVs, RA, SLE | 153 | Neubauer 1997a^130^ | HVs, HFrEF |
| 66 | Beadle 2015^131^ | HFrEF | 154 | Neubauer 1997b^132^ | HVs, AS, AR |
| 67 | Madathil 2015^133^ | HVs, subclinical hypothyroidism | 155 | Kalil 1997^134^ | HVs, IHD |
| 68 | Geier 2014^135^ | HVs | 156 | Sieverding 1997^136^ | HVs, HCM |
| 69 | Dass 2014^137^ | HVs, Hypertension | 157 | Hochachka 1996^138^ | HVs, Sherpas |
| 70 | Esterhammer 2014^139^ | HVs | 158 | Pluim 1996^140^ | HVs, athletes |
| 71 | Cassidy 2014^141^ | HVs, T2DM, NAFLD | 159 | Lamb 1996^142^ | HVs |
| 72 | Jakovljevic 2014^143^ | HVs | 160 | Hetherington 1995^144^ | HVs |
| 73 | Rodgers 2014^145^ | HVs | 161 | Nishiyama 1995^146^ | HVs, HFrEF |
| 74 | Mahmod 2014^147^ | HVs, AS | 162 | Yabe 1994^148^ | HVs, IHD |
| 75 | Bashir 2014^149^ | HVs | 163 | Kuno 1994^150^ | HVs, athletes |
| 76 | Lewis 2014^151^ | GFPT1 & DPAGT1 mutations | 164 | Doornbos 1994^152^ | SS |
| 77 | Hollingsworth 2013^153^ | HVs, LGMD2L | 165 | Van Dobbenburgh 1994^154^ | HVs |
| 78 | Bates 2013^155^ | HVs, mitochondrial disease | 166 | Sakuma 1993^156^ | HVs, HCM |
| 79 | Leme 2013^157^ | HVs, Chagas heart disease | 167 | Neubauer 1992^158^ | HVs, HFrEF, IHD |
| 80 | Banks 2013a^159^ | HVs | 168 | De Roos 1992^160^ | HVs, HCM, HFrEF |
| 81 | Banks 2013b^161^ | HVs | 169 | Masuda 1992^162^ | HVs, HFrEF |
| 82 | Rider 2013^163^ | HVs, obesity | 170 | Schaefer 1992^164^ | HVs, HFrEF |
| 83 | Spoladore 2013^165^ | HFrEF | 171 | Conway 1991^166^ | AS, AR |
| 84 | Hirsch 2012^167^ | HFrEF | 172 | Auffermann 1991^168^ | HVs, HFrEF |
| 85 | Rider 2012^169^ | HVs, obesity | 173 | Schaefer 1990^170^ | HVs, HFrEF |
| 86 | Mazaev 2012^171^ | HVs, HCM | 174 | Weiss 1990^172^ | HVs, IHD |
| 87 | Holloway 2012^173^ | HFrEF | 175 | Schaefer 1988^174^ | HVs |
| 88 | Malatesta-Muncher 2012^175^ | HVs, dialysis, kidney transplant | 176 | Rajagopalan 1987^176^ | HVs |

**Supplementary Table 3** – Cardiac PCr/ATP in cardiovascular disease

| Disease | Study | PCr/ATP (SD) | N | Field Strength | Position | Sequence | Voxel |
| --- | --- | --- | --- | --- | --- | --- | --- |
| Hypertension | |  |  |  |  |  |  |
|  | Dass et al, 2014 | 1.63 (0.07) | 17 | 3T | Prone | - | - |
|  | Patel et al, 2011 | 1.78 (0.35) | 30 | 1.5T | - | CSI | - |
|  | Burkhard et al, 2009 | 1.34 (0.28) | 20 | 1.5T | - | CSI | Apex |
|  | Heyne et al, 2006 | 1.65 (0.25) | 25 | 1.5T | Prone | CSI | Anteroseptum |
|  | Beer et al, 2002 | 1.51 (0.43) | 10 | 1.5T | Prone | CSI |  |
|  | Lamb et al, 1999 | 1.2 (0.18) | 11 | 1.5T | Supine | ISIS | Septum |
| Ischaemic Heart Disease | |  |  |  |  |  |  |
| Ischaemia |  |  |  |  |  |  |  |
|  | Kotha et al, 2024^a^ | 1.5 (0.42) | 30 | 3T | - | - | - |
|  | Kotha et al, 2024^b^ | 1.8 (0.56) | 30 | 3T | - | - | - |
|  | Najjar et al, 2005 | 1.39 (0.32) | 12 | 1.5T | Prone | - | - |
|  | Neubauer et al, 1992 | 1.77 (0.13) | 4 | 1.5T | Prone | ISIS | Anterior wall |
|  | Weiss et al, 1990 | 1.45 (0.31) | 16 | 1.5T | Prone | ISIS | Anterior wall |
| Infarction |  |  |  |  |  |  |  |
|  | Bottomley et al, 2009 | 1.74 (0.27) | 15 | 1.5T | Prone | CSI | Anteroseptum |
|  | Beer et al, 2007^c^ | 1.17 (0.25) | 15 | 1.5T | Prone | CSI | Anterior wall |
|  | Beer et al, 2007^d^ | 0.81 (0.60) | 15 | 1.5T | Prone | CSI | Anterior wall |
|  | Beer et al, 2004 | 1.03 (0.39) | 8 | 1.5T | Prone | CSI | Inferior wall |
|  | Moka et al, 2001 | 1.74 (0.23) | 15 | 1.5T | Supine | ISIS | Anterior wall |
|  | Beer et al, 2000^c^ | 1.47 (0.38) | 10 | 1.5T | Prone | CSI | Anterior wall |
|  | Kalil-Filho et al, 1997 | 1.51 (0.17) | 29 | 1.5T | Supine | ISIS | Anterior wall |
|  | Yabe et al, 1994^c^ | 1.56 (0.19) | 15 | 1.5T | Supine | DRESS | Anterior wall |
|  | Yabe et al, 1994^d^ | 1.18 (0.19) | 12 | 1.5T | Supine | DRESS | Anterior wall |
|  | Neubauer et al, 1992 | 1.86 (0.45) | 6 | 1.5T | Prone | ISIS | Anterior wall |
| HFimpEF |  |  |  |  |  |  |  |
|  | Goldenberg et al, 2023 | 1.76 | 15 | 3T | - | - | - |
| Amyloid |  |  |  |  |  |  |  |
|  | Burrage et al, 2021 | 1.33 (0.27) | 9 | 3T | Prone | CSI | Basal septum |
| Atrial Fibrillation | |  |  |  |  |  |  |
|  | Wijesurendra et al, 2016 | 1.81 (0.35) | 53 | 3T | Prone | CSI | Mid-septum |
| Takotsubo |  |  |  |  |  |  |  |
|  | Scally et al, 2018 | 1.3 (0.1) | 37 | 3T | - | - | Septum |
| Eisenmenger syndrome | |  |  |  |  |  |  |
|  | Bowater et al, 2016 | 1.55 (0.1) | 10 | 3T | Supine | ISIS | Apical septum |
| Chagas Heart Disease | |  |  |  |  |  |  |
|  | Leme et al, 2013 | 1.23 (0.37) | 28 | 1.5T | Supine | ISIS | Anteroapex |
| Pulmonary Regurgitation | |  |  |  |  |  |  |
|  | Kotha et al, 2024^e^ | 2.2 | 3 | 3T | - | - | Septum |
|  | Kotha et al, 2024^f^ | 1.7 | 4 | 3T | - | - | Septum |
| Cardiac Transplant | |  |  |  |  |  |  |
|  | Caus et al, 2006^g^ | 1.51 (0.5) | 8 | 1.5T | Supine | CSI | Anteroseptum |
|  | Caus et al, 2006^h^ | 1.98 (0.53) | 18 | 1.5T | Supine | CSI | Anteroseptum |
|  | Buchthal et al, 2000 | 1.27 (0.27) | 13 | 1.5T | Supine | CSI | - |

^a^ Type 2 diabetes, ^b^ No type 2 diabetes, ^c^ Viable myocardium, ^d^ Non-viable myocardium, ^e^ Mild-moderate pulmonary regurgitation, ^f^ Severe pulmonary regurgitation, ^g^ Cardiac allograft vasculopathy, ^h^ No cardiac allograft vasculopathy

**Supplementary Table 4** – Cardiac PCr/ATP in systemic disease and physiological states

| Disease Group | Population | Study | PCr/ATP (SD) | N | Field Strength | Position | Sequence | Voxel |
| --- | --- | --- | --- | --- | --- | --- | --- | --- |
| Cancer |  |  |  |  |  |  |  |  |
|  | Post-Anthracyclines for Breast Cancer | Gamble et al, 2023 | 1.1 (0.5) | 25 | 3T | Supine | ISIS | Septum |
|  | Post-Anthracyclines for Childhood Cancer | Eidenschink et al, 2000 | 1.09 (0.43) | 62 | 1.5T | - | ISIS | Anterior wall |
| Rheumatology |  |  |  |  |  |  |  |  |
|  | Rheumatoid arthritis | Soo et al, 2025 | 1.8 (0.4) | 27 | 3T | - | CSI | - |
|  |  | Ntusi et al, 2015 | 1.5 (0.3) | 16 | 1.5T | - | - | - |
|  | Systemic Sclerosis | Soo et al, 2025 | 1.93 (0.5) | 38 | 3T | - | CSI | - |
|  |  | Doornbos et al, 1994 | 1.65 (0.26) | 5 | 1.5T | Supine | ISIS | Apex |
|  | Systemic Lupus Erythematosus | Ntusi et al, 2015 | 1.4 (0.3) | 13 | 1.5T | - | - | - |
| Endocrine |  |  |  |  |  |  |  |  |
|  | Pre-diabetes | de Wit-Verheggen et al, 2025 | 1.03 (0.08) | 8 | 3T | Prone | ISIS | Anterior wall |
|  | Growth Hormone Deficiency | Cobelli et al, 2029 | 2.1 (0.8) | 15 | 1.5T | - | ISIS | - |
|  | Subclinical Hypothyroidism | Madathil et al, 2015 | 1.80 (0.26) | 21 | 3T | Prone | CSI | - |
|  | Familial Hypercholesterolaemia | Schocke et al, 2003 | 1.78 (0.34) | 13 | 1.5T | - | CSI | - |
| Renal |  |  |  |  |  |  |  |  |
|  | CKD5 pre-dialysis | Patel et al, 2012 | 1.3 (0.5) | 53 | 1.5T | - | CSI | Apex |
|  | Dialysis | Ng et al, 2024 | 1.5 (0.25) | 11 | 3T | Supine | DRESS | - |
|  |  | Malatesta-Muncher et al, 2012 | 0.99 (0.48) | 10 | 3T | Supine | ISIS | Anteroseptum |
|  |  | Ogimoto et al, 2003 | 1.03 (0.15) | 14 | - | - | ISIS | Anteroseptum |
|  |  | Tagami et al, 1998 | 1.15 (0.25) | 14 | 1.5T | Prone | DRESS | Anterospetum |
|  | Kidney Transplant | Malatesta-Muncher et al, 2012 | 1.34 (0.78) | 10 | 3T | Supine | ISIS | Anteroseptum |
|  |  | Perseghin et al, 2005 | 1.66 (0.07) | 16 | 1.5T | - | ISIS | - |
|  | Simultaneous Pancreas-Kidney (SPK) Transplant | Perseghin et al, 2005 | 1.68 (0.11) | 9 | 1.5T | - | ISIS | - |
| Neurology |  |  |  |  |  |  |  |  |
|  | Friedreich's Ataxia | Wolf et al, 2007 | 2.01 (0.37) | 10 | 1.5T | Supine | CSI | Anteroseptum |
|  |  | Lodi et al, 2001 | 1.46 (0.53) | 18 | 2T | Prone | CSI | Septum, apex and posterior wall summed |
|  | Myotonic Dystrophy | Beer et al, 2002 | 1.5 (0.4) | 3 | 1.5T | Prone | CSI | - |
|  | Becker Muscular Dystrophy | Crilley et al, 2000 | 1.55 (0.37) | 13 | 2T | Prone | CSI | - |
|  |  | Crilley et al, 2000^a^ | 1.37 (0.25) | 10 | 2T | Prone | CSI | - |
|  | Limb Girdle Muscular Dystrophy 2L | Hollingsworth et al, 2014 | 1.5 (0.24) | 10 | 3T | Prone | CSI | - |
|  | GFPT1 mutations | Lewis et al, 2014 | 2.24 | 2 | 3T | Prone | CSI | Mid-septum |
|  | DPAGT1 mutations | Lewis et al, 2014 | 1.1 | 2 | 3T | Prone | CSI | Mid-septum |
|  | Multiple Sclerosis | Spindler et al, 2003 | 1.43 (0.23) | 14 | 1.5T | Supine | CSI | Septum |
|  | Chronic Fatigue Syndrome | Hollingsworth et al, 2010 | 1.57 (0.22) | 12 | - | - | - | - |
| Infectious Disease | |  |  |  |  |  |  |  |
|  | Prior COVID | Chamley et al, 2021 | 2.24 (0.3) | 88 | 3T | Prone | CSI | Basal septum |
|  | Long COVID | Gorecka et al, 2022 | 2.24 (0.4) | 19 | 3T | Supine | CSI | Mid-septum |
| Hepatology |  |  |  |  |  |  |  |  |
|  | Haemochromatosis | Schocke et al, 2005 | 1.6 (0.41) | 24 | 1.5T | Supine | CSI | - |
|  | NAFLD | Cassidy et al, 2015 | 1.8 (0.3) | 19 | 3T | Prone | CSI | - |
|  |  | Perseghin et al, 2008 | 1.84 (0.34) | 21 | 3T | Supine | ISIS | - |
|  | Primary Biliary Cirrhosis | Jones et al, 2010 | 1.64 (0.06) | 15 | 3T | Prone | CSI | - |
| Mitochondrial disease | |  |  |  |  |  |  |  |
|  | A3243G mtDNA mutation | Bates et al, 2013 | 1.45 (0.42) | 10 | 3T | Prone | CSI | - |
|  |  | Lodi et al, 2004 | 1.55 (0.58) | 8 | 2T | Prone | CSI | - |
| Athletes |  |  |  |  |  |  |  |  |
|  | Athletes | Henry et al, 2022 | 2.23 (0.28) | 17 | 3T | Supine | CSI | Basal septum |
|  | Veteran athletes | Chowdhary et al, 2021 | 2.07 (0.38) | 12 | - | - | - | - |
|  | Cyclists | Pluim et al, 1999 | 1.44 (0.18) | 12 | 1.5T | Supine | ISIS | Anterior wall |
|  |  | Pluim et al, 1998 | 1.41 (0.2) | 21 | 1.5T | Supine | ISIS | Septum |
|  |  | Pluim et al, 1996 | 2.2 (0.34) | 13 | 1.5T | Supine | ISIS | Anterior wall |
|  | Long distance runners | Kuno et al, 1994 | 1.51 (0.02) | 6 | 1.5T | Supine | DRESS | Apex |
| Pregnancy |  |  |  |  |  |  |  |  |
|  | Healthy pregnancy | Thirunavukarasu et al, 2025 | 2.2 (0.47) | 38 | 3T | Supine | CSI | Mid-septum |
|  | Gestational diabetes | Thirunavukarasu et al, 2025 | 1.9 (0.53) | 30 | 3T | Supine | CSI | Mid-septum |
|  | Pre-eclampsia | Thirunavukarasu et al, 2025 | 1.9 (0.34) | 20 | 3T | Supine | CSI | Mid-septum |
|  | Pre-term adults | Burchert et al, 2025 | 1.8 (0.4) | 38 | 3T | - | - | - |
| Altitude |  |  |  |  |  |  |  |  |
|  | Everest base camp | Holloway et al, 2011 | 1.68 (0.08) | 14 | 3T | Prone | CSI | Mid-septum |
|  | Sherpas | Hochachka et al, 1996 | 0.96 (0.22) | 6 | 4T | - | CSI | - |

^a^BMD female carriers

**Supplementary Table 5** – Risk of bias

| **Study #** | **Study** | **Risk of Bias** | **Notes** | **Study #** | **Study** | **Risk of Bias** | **Notes** |
| --- | --- | --- | --- | --- | --- | --- | --- |
| 1 | Procter 2025^1^ | Low |  | 89 | Perseghin 2012^2^ | Low |  |
| 2 | Ashkir 2025^3^ | Moderate | Unclear population frame | 90 | Mannacio 2012^4^ | Low |  |
| 3 | Birkhoelzer 2025^5^ | Low |  | 91 | Kuehl 2012^6^ | Low |  |
| 4 | de Wit-Verheggen 2025^7^ | Moderate | Small sample size | 92 | Patel 2011^8^ | Moderate | Limited methodological details |
| 5 | Giannoudi 2025^9^ | Low |  | 93 | Holloway 2011a^10^ | Moderate | Generalisability is unclear |
| 6 | Karkouri 2025^11^ | High | Feasibility study | 94 | Holloway 2011b^12^ | Moderate | Generalisability is unclear |
| 7 | Soo 2025a^15^ | Low |  | 95 | Suttie 2011^14^ | Low |  |
| 8 | Soo 2025b^13^ | Low |  | 96 | Beadle 2011^16^ | Low |  |
| 9 | Thirunavukarasu 2025^19^ | Low |  | 97 | Abraham 2011^18^ | Moderate | Small sample size |
| 10 | Burchert 2024^17^ | Moderate | Limited methodological information | 98 | Fragasso 2011^20^ | Moderate | Generalisability is unclear |
| 11 | Birkhoelzer 2024^21^ | Low |  | 99 | Jones 2010^22^ | Moderate | Small sample size |
| 12 | Giannoudi 2024^23^ | Moderate | Little detail on group characteristics | 100 | Shivu 2010a^24^ | Moderate | Small sample size |
| 13 | Kotha 2024a^25^ | Moderate | Little detail on group characteristics | 101 | Shivu 2010b^26^ | Low |  |
| 14 | Kotha 2024b^27^ | Moderate | Little detail on group characteristics | 102 | Abozguia 2010^28^ | Low |  |
| 15 | Kotha 2024c^29^ | Moderate | Small sample size | 103 | Holloway 2010^30^ | Moderate | Generalisability is unclear |
| 16 | Ng 2024^31^ | Low |  | 104 | Hollingsworth 2010^32^ | Low |  |
| 17 | Chowdhary 2024^33^ | Low |  | 105 | Hudsmith 2009^34^ | Low |  |
| 18 | Hundertmark 2024^35^ | Low |  | 106 | Burkhard 2009^36^ | High | Limited methodological details, Small sample size |
| 19 | Chamley 2024^37^ | Moderate | Limited methodological details | 107 | Phan 2009^38^ | Low |  |
| 20 | Hundertmark 2023^39^ | Low |  | 108 | Esposito 2009^40^ | Low |  |
| 21 | Jex 2023^41^ | Low |  | 109 | Bottomley 2009^42^ | Low |  |
| 22 | Goldenberg 2023^43^ | Moderate | Limited methodlogical information | 110 | Tyler 2009^44^ | Low |  |
| 23 | Watson 2023^45^ | Moderate | Small sample size | 111 | Beer 2008^46^ | Low |  |
| 24 | Monga 2023^47^ | Low |  | 112 | Perseghin 2008^48^ | Low |  |
| 25 | Van de Bovenkamp 2023^49^ | Moderate | Generalisability is unclear | 113 | Wolf 2008^50^ | Moderate | Small sample size |
| 26 | Gamble 2023^51^ | Moderate | Recruitment methodology lacking | 114 | Klug 2007^52^ | Low |  |
| 27 | Papalia 2022^53^ | Low |  | 115 | Perseghin 2007^54^ | Low |  |
| 28 | Gorecka 2022^55^ | Moderate | Small sample size | 116 | Beer 2007^56^ | Low |  |
| 29 | Jex 2022^57^ | Moderate | Recruitment methodology lacking | 117 | Van Der Meer 2007^58^ | Low |  |
| 30 | Reid 2022^59^ | Moderate | Limited methodological details | 118 | Heyne 2006^60^ | Moderate | Limited information on sampling |
| 31 | Cameron 2022^61^ | Low |  | 119 | Caus 2006^62^ | High | Unclear reporting of average PCr/ATP, Small sample size |
| 32 | Valkovic 2022^63^ | Low |  | 120 | Perseghin 2005^64^ | Moderate | Small sample size |
| 33 | Chowdhary 2022a^65^ | Moderate | Recruitment methodology lacking | 121 | Najjar 2005^66^ | Moderate | Unclear reporting of average PCr/ATP |
| 34 | Chowdhary 2022b^67^ | Moderate | Limited methodological details | 122 | Chida 2005^68^ | High | Technical and feasibility focussed |
| 35 | Henry 2022^69^ | Moderate | Limited methodological details | 123 | Hansch 2005^70^ | Moderate | Small sample size |
| 36 | Wibowo 2021^71^ | Moderate | Little detail on group characteristics | 124 | Schocke 2004^72^ | Low |  |
| 37 | Chowdhary 2021^73^ | Moderate | Possible selection bias | 125 | Beer 2004^74^ | Moderate | Small sample size |
| 38 | Thirunavukarasu 2021^75^ | Moderate | Small sample size | 126 | Lodi 2004^76^ | Moderate | Small sample size |
| 39 | Burrage 2021^77^ | Moderate | Small sample size | 127 | Schocke 2003a^78^ | Low |  |
| 40 | Gaborit 2021^79^ | Low |  | 128 | Schocke 2003b^80^ | Low |  |
| 41 | Watson 2021^81^ | High | Generalisability is unclear, Small sample size | 129 | Scheuermann-Freestone 2003^82^ | Low |  |
| 42 | Rayner 2021^83^ | Low |  | 130 | Ogimoto 2003^84^ | High | Limited outcome reporting detail, Small sample size |
| 43 | Apps 2021^85^ | Low |  | 131 | Diamant 2003^86^ | Low |  |
| 44 | Lewis 2021^87^ | Low |  | 132 | Crilley 2003^88^ | Low |  |
| 45 | Rayner 2020^89^ | Low |  | 133 | Spindler 2003^90^ | Moderate | Generalisability is unclear |
| 46 | Rider 2020^91^ | Low |  | 134 | Beer 2002a^92^ | Moderate | Generalisability is unclear |
| 47 | Peterzan 2020^93^ | Low |  | 135 | Beer 2002b^94^ | Low |  |
| 48 | Ellis 2019^95^ | Moderate | Small sample size | 136 | Metzler 2002^96^ | Low |  |
| 49 | De Cobelli 2019^97^ | Moderate | Generalisability is unclear | 137 | Pohmann 2001^98^ | High | Technical and feasibility focussed |
| 50 | Stoll 2019^99^ | Moderate | Small sample size | 138 | Beyerbacht 2001^100^ | Moderate | Generalisability is unclear |
| 51 | Nathania 2018^101^ | Low |  | 139 | Lodi 2001^102^ | High | Generalisability is unclear, Small sample size |
| 52 | Mahmod 2018^103^ | Low |  | 140 | Moka 2001^104^ | High | Limited recruitment details, Small sample size |
| 53 | Scally 2018^105^ | Low |  | 141 | Beer 2000^106^ | Moderate | Small sample size |
| 54 | Peterzan 2018^107^ | Low |  | 142 | Crilley 2000^108^ | Moderate | Small sample size |
| 55 | Rayner 2018^109^ | Low |  | 143 | Eidenschink 2000^110^ | Low |  |
| 56 | Valkovic 2017^111^ | High | Generalisability is unclear, Small sample size | 144 | Buchthal 2000^112^ | Moderate | Generalisability is unclear |
| 57 | Stoll 2016^113^ | Low |  | 145 | Pluim 1999^114^ | Moderate | Small sample size |
| 58 | Bowater 2016^115^ | Moderate | Small sample size | 146 | Lamb 1999^116^ | Low |  |
| 59 | Levelt 2016a^117^ | Low |  | 147 | Nanbu 1999^118^ | Moderate | Small sample size |
| 60 | Levelt 2016b^119^ | Low |  | 148 | Jung 1998^120^ | Low |  |
| 61 | Wijesurendra 2016^121^ | Low |  | 149 | Tagami 1998^122^ | Moderate | Small sample size |
| 62 | Salerno 2015^123^ | Moderate | Generalisability is unclear | 150 | Pluim 1998^124^ | Low |  |
| 63 | Dass 2015a^125^ | Low |  | 151 | Loffler 1998^126^ | High | Technical and feasibility focussed, Small sample size |
| 64 | Dass 2015b^127^ | Low |  | 152 | Conway 1998^128^ | Moderate | Small sample size |
| 65 | Ntusi 2015^129^ | Low |  | 153 | Neubauer 1997a^130^ | Low |  |
| 66 | Beadle 2015^131^ | Moderate | Generalisability is unclear | 154 | Neubauer 1997b^132^ | Moderate | Small sample size |
| 67 | Madathil 2015^133^ | Moderate | Generalisability is unclear | 155 | Kalil 1997^134^ | Moderate | Small sample size |
| 68 | Geier 2014^135^ | High | Technical methods focus, Small sample size | 156 | Sieverding 1997^136^ | Low |  |
| 69 | Dass 2014^137^ | Low |  | 157 | Hochachka 1996^138^ | Moderate | Small sample size |
| 70 | Esterhammer 2014^139^ | Low |  | 158 | Pluim 1996^140^ | Moderate | Small sample size |
| 71 | Cassidy 2014^141^ | Low |  | 159 | Lamb 1996^142^ | Moderate | Methodological repeatability focussed |
| 72 | Jakovljevic 2014^143^ | Low |  | 160 | Hetherington 1995^144^ | High | Technical and feasibility focussed, Small sample size |
| 73 | Rodgers 2014^145^ | Low |  | 161 | Nishiyama 1995^146^ | Low |  |
| 74 | Mahmod 2014^147^ | Low |  | 162 | Yabe 1994^148^ | Low |  |
| 75 | Bashir 2014^149^ | Moderate | Limited PCr/ATP context for target disease groups | 163 | Kuno 1994^150^ | High | Generalisability is unclear, Small sample size |
| 76 | Lewis 2014^151^ | Low |  | 164 | Doornbos 1994^152^ | High | Limited recruitment details, Small sample size |
| 77 | Hollingsworth 2013^153^ | Moderate | Small sample size | 165 | Van Dobbenburgh 1994^154^ | High | Technical and feasibility focussed, Small sample size |
| 78 | Bates 2013^155^ | Moderate | Small sample size | 166 | Sakuma 1993^156^ | Moderate | Small sample size |
| 79 | Leme 2013^157^ | Moderate | Small sample size | 167 | Neubauer 1992^158^ | Moderate | Small sample size |
| 80 | Banks 2013a^159^ | Moderate | Small sample size | 168 | De Roos 1992^160^ | Moderate | Small sample size |
| 81 | Banks 2013b^161^ | Low |  | 169 | Masuda 1992^162^ | High | Unclear generalisability, outcome reporting and sampling, Small sample size |
| 82 | Rider 2013^163^ | Low |  | 170 | Schaefer 1992^164^ | Moderate | Small sample size |
| 83 | Spoladore 2013^165^ | Moderate | Small sample size | 171 | Conway 1991^166^ | Moderate | Small sample size |
| 84 | Hirsch 2012^167^ | Low |  | 172 | Auffermann 1991^168^ | Moderate | Potential for coverage bias |
| 85 | Rider 2012^169^ | Low |  | 173 | Schaefer 1990^170^ | Moderate | Small sample size |
| 86 | Mazaev 2012^171^ | High | Possible selective reporting and unclear generalisability | 174 | Weiss 1990^172^ | Low |  |
| 87 | Holloway 2012^173^ | Low |  | 175 | Schaefer 1988^174^ | Moderate | Generalisability is unclear |
| 88 | Malatesta-Muncher 2012^175^ | Moderate | Small sample size | 176 | Rajagopalan 1987^176^ | High | Technical and feasibility focussed, use of outdated methodology, Small sample size |

**Supplementary Table 6** – Sensitivity analysis

| **Aspect interrogated** | **Outcome** | **PCr/ATP ratio**  **(95% CI)** |
| --- | --- | --- |
| Risk of bias | Healthy volunteers | 2.02 (1.96 to 2.08) |
|  | T1DM | 1.88 (1.79 to 1.98) |
|  | T2DM | 1.55 (1.43 to 1.67) |
|  | HFrEF | 1.59 (1.50 to 1.68) |
|  | HFpEF | 1.60 (1.57 to 1.63) |
|  | HCM | 1.78 (1.59 to 1.97) |
|  | Severe AS | 1.61 (1.50 to 1.73) |
|  | Moderate AS | 1.47 (1.20 to 1.73) |
|  | Mild AS | 1.53 (1.22 to 1.84) |
|  | Aortic regurgitation | - |
|  | Severe MR | - |
|  | Moderate MR | - |
|  | Mild MR | - |
| Fixed effects meta-analysis | Healthy volunteers | 1.94 (1.93 to 1.94) |
|  | T1DM | 1.76 (1.69 to 1.84) |
|  | T2DM | 1.55 (1.53 to 1.58) |
|  | HFrEF | 1.59 (1.58 to 1.61) |
|  | HFpEF | 1.61 (1.58 to 1.64) |
|  | HCM | 1.32 (1.31 to 1.34) |
|  | Severe AS | 1.68 (1.67 to 1.69) |
|  | Moderate AS | 1.57 (1.54 to 1.59) |
|  | Mild AS | 1.53 (1.22 to 1.84) |
|  | Aortic regurgitation | 1.73 (1.66 to 1.80) |
|  | Severe MR | 1.46 (1.34 to 1.57) |
|  | Moderate MR | 1.53 (1.42 to 1.63) |
|  | Mild MR | 1.73 (1.59 to 1.87) |
| Full text only | Healthy volunteers | 1.92 (1.86 to 1.98) |
|  | T1DM | 1.77 (1.54 to 2.00) |
|  | T2DM | 1.60 (1.51 to 1.68) |
|  | HFrEF | 1.53 (1.43 to 1.63) |
|  | HFpEF | 1.60 (1.57 to 1.64) |
|  | HCM | 1.59 (1.41 to 1.76) |
|  | Severe AS | 1.42 (1.23 to 1.61) |
|  | Moderate AS | 1.30 (1.04 to 1.57) |
|  | Mild AS | - |
|  | Aortic regurgitation | - |
|  | Severe MR | 1.29 (1.10 to 1.48) |
|  | Moderate MR | - |
|  | Mild MR | - |

**Supplementary Figure 1.** Forest plot of PCr/ATP ratio for healthy volunteers.


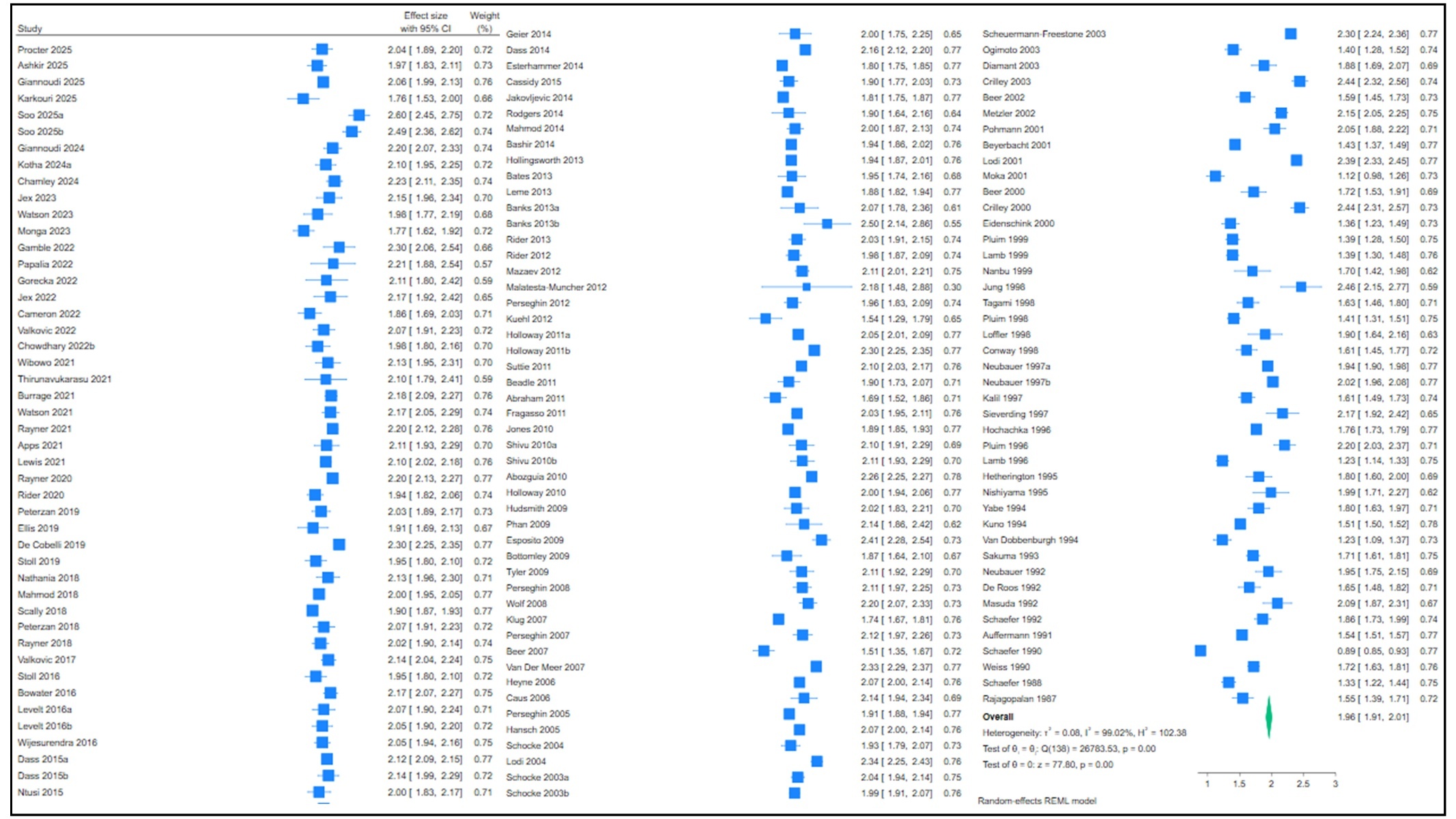


**Supplementary Figure 2** – Meta-analysis of healthy adults subgrouped by sequence.


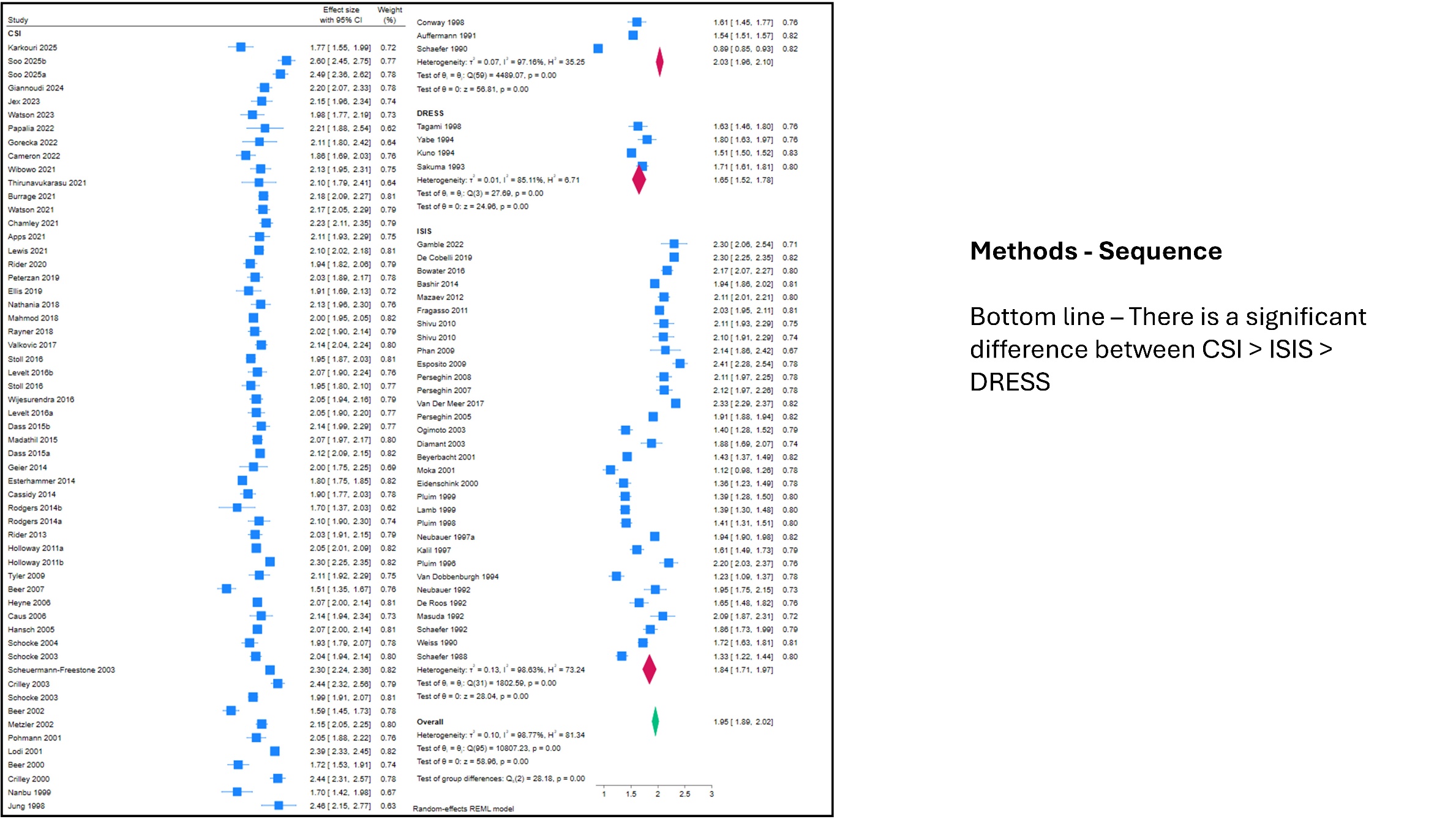


**Supplementary Figure 3** – A) Field strength, B) repetition time (TR) meta-regression


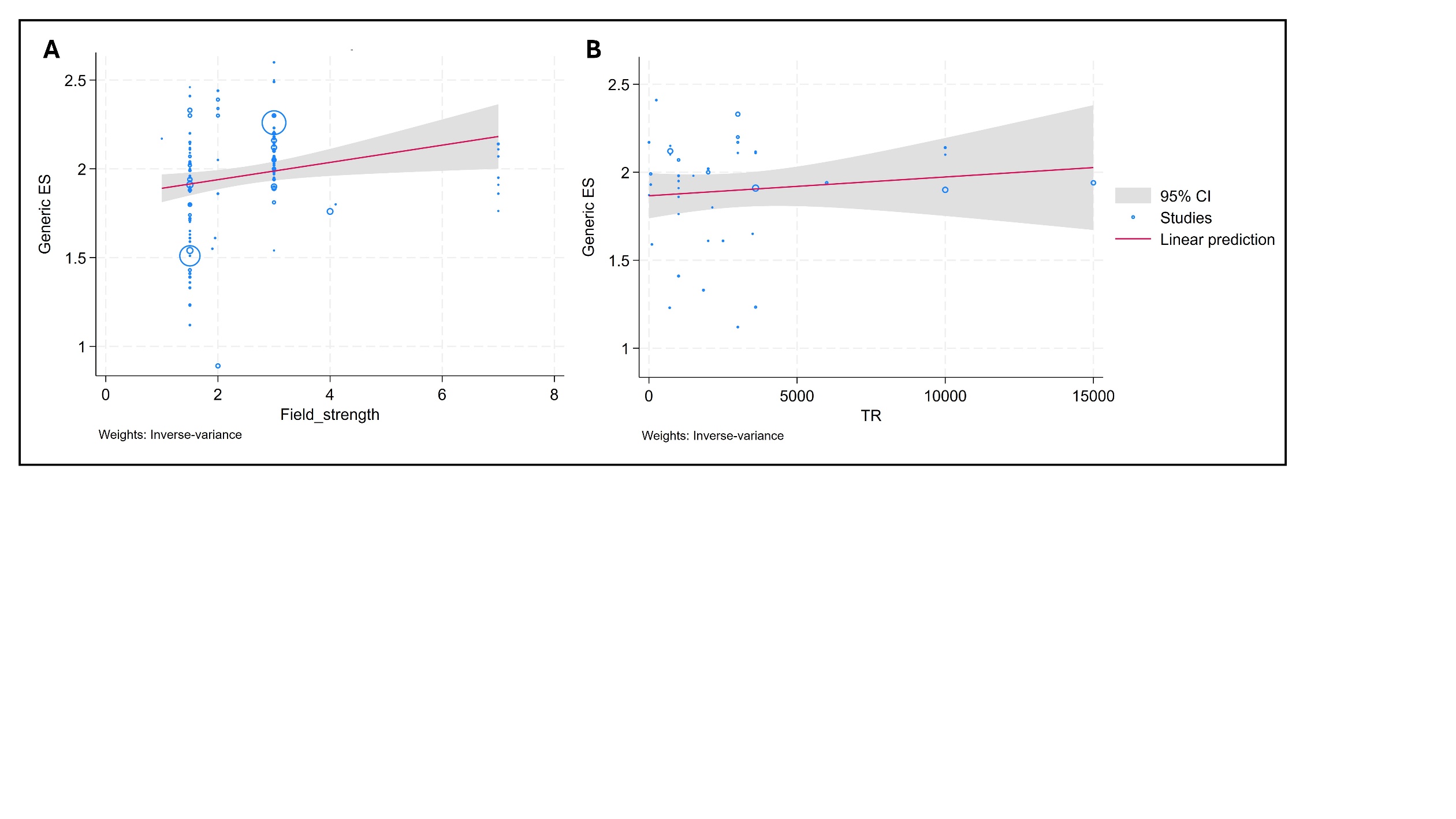


**Supplementary Figure 4** – Meta-analysis of healthy adults subgrouped by location imaged.


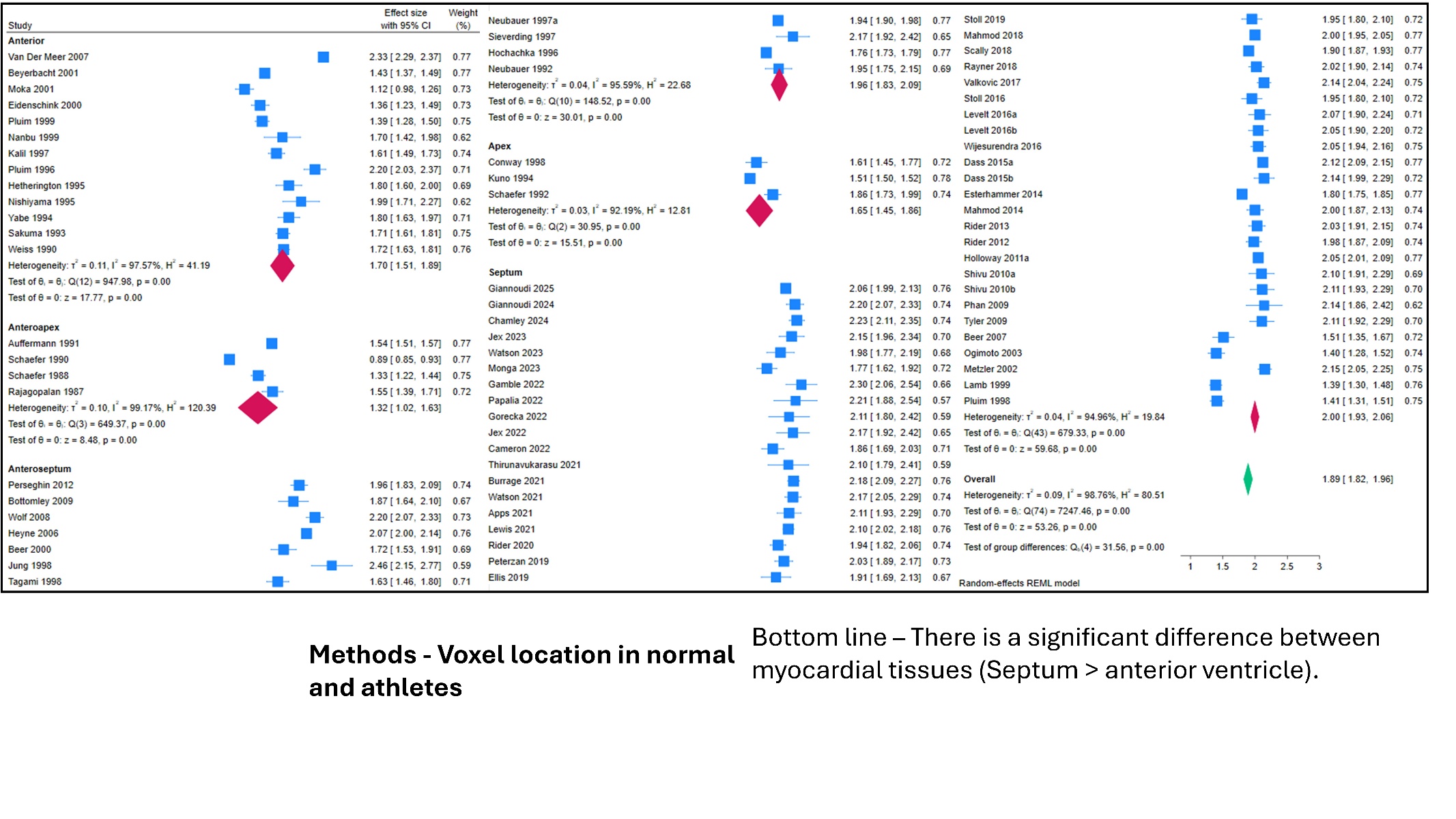


**Supplementary Figure 5** – Meta-analysis of healthy volunteers subgrouped by position.


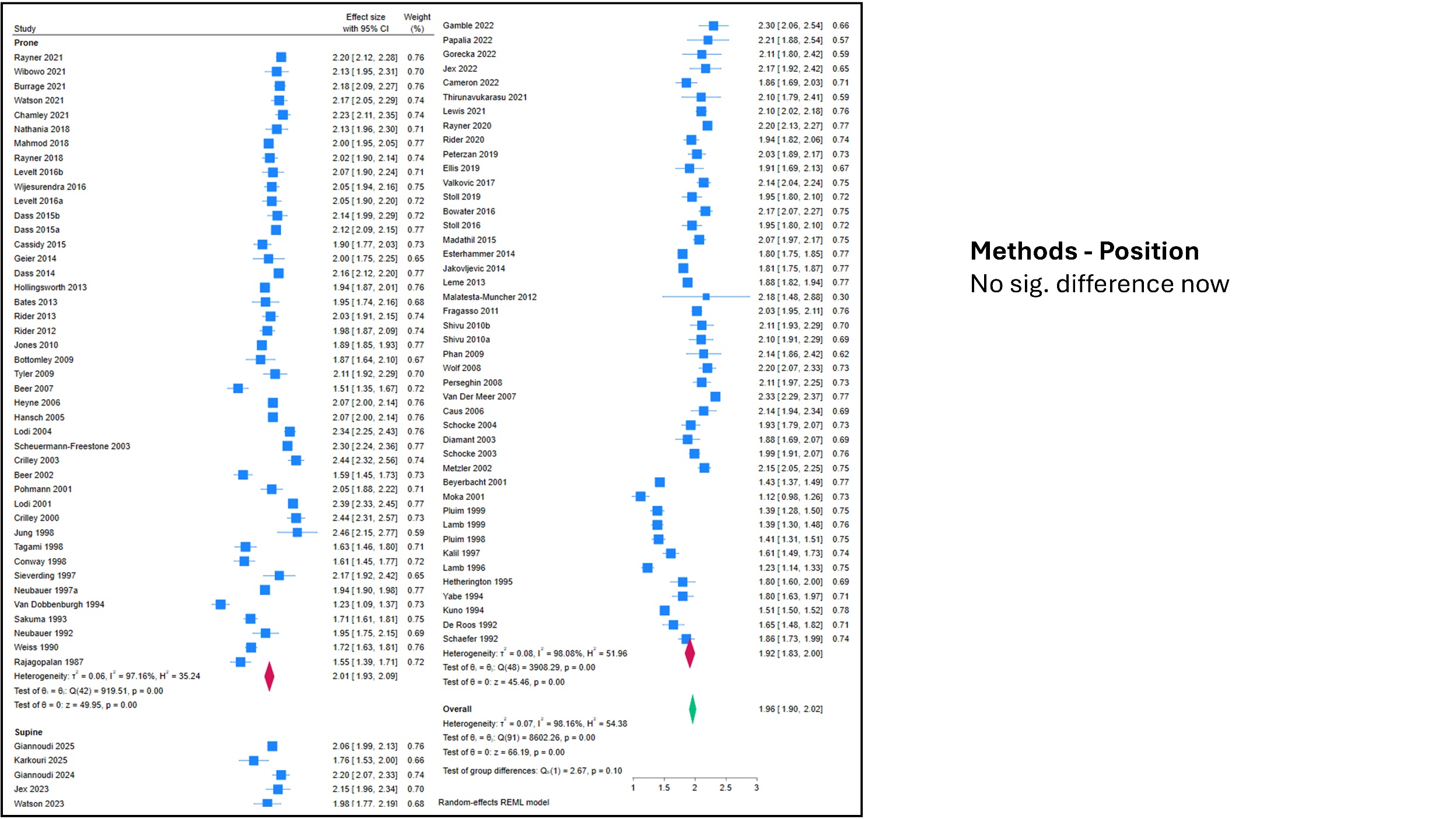


**Funnel plots**

**Supplementary Figure 6A** – Funnel plot of the meta-analysis for pooled average PCr/ATP ratio for healthy volunteers.


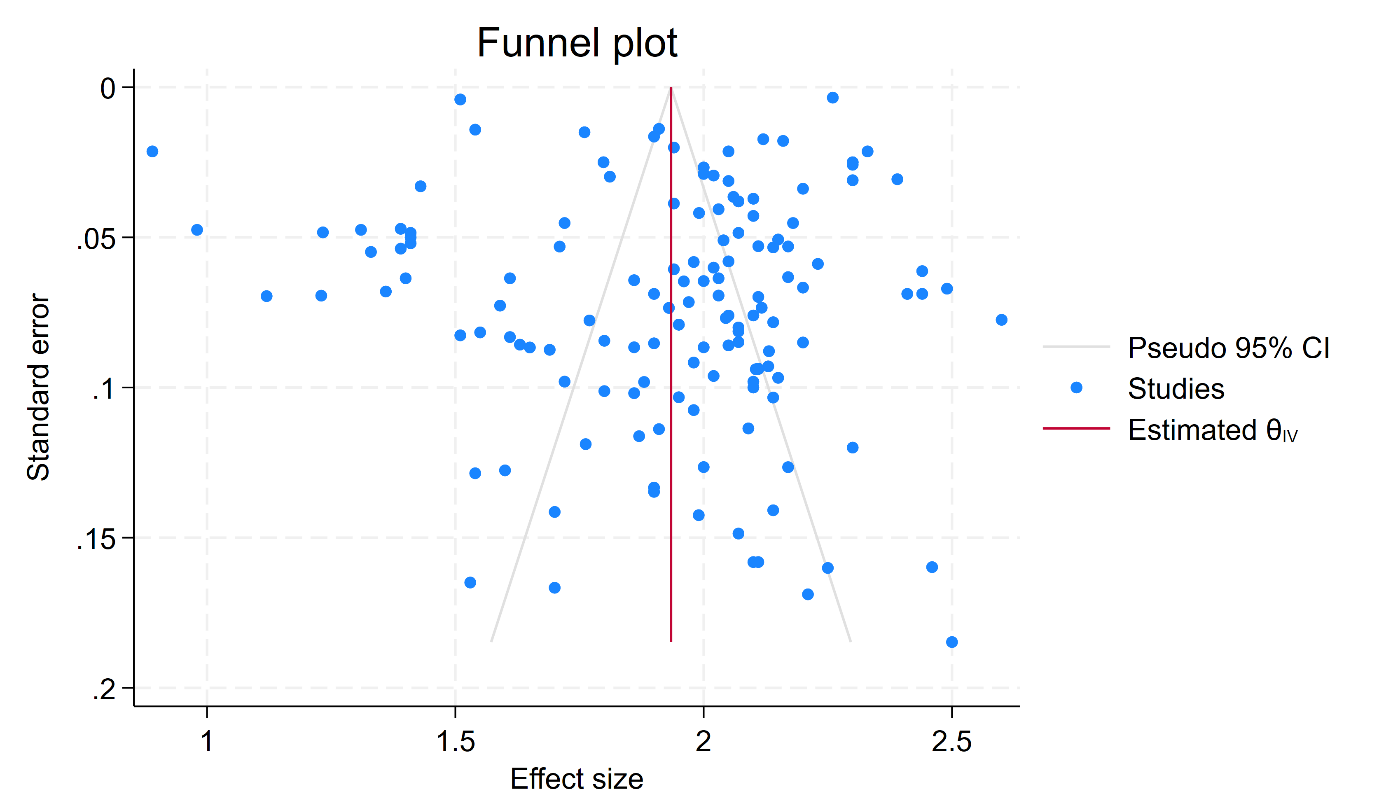


**Supplementary Figure 6B** – Funnel plot of the meta-analysis for pooled average PCr/ATP ratio for patients with heart failure with reduced ejection fraction (HFrEF).


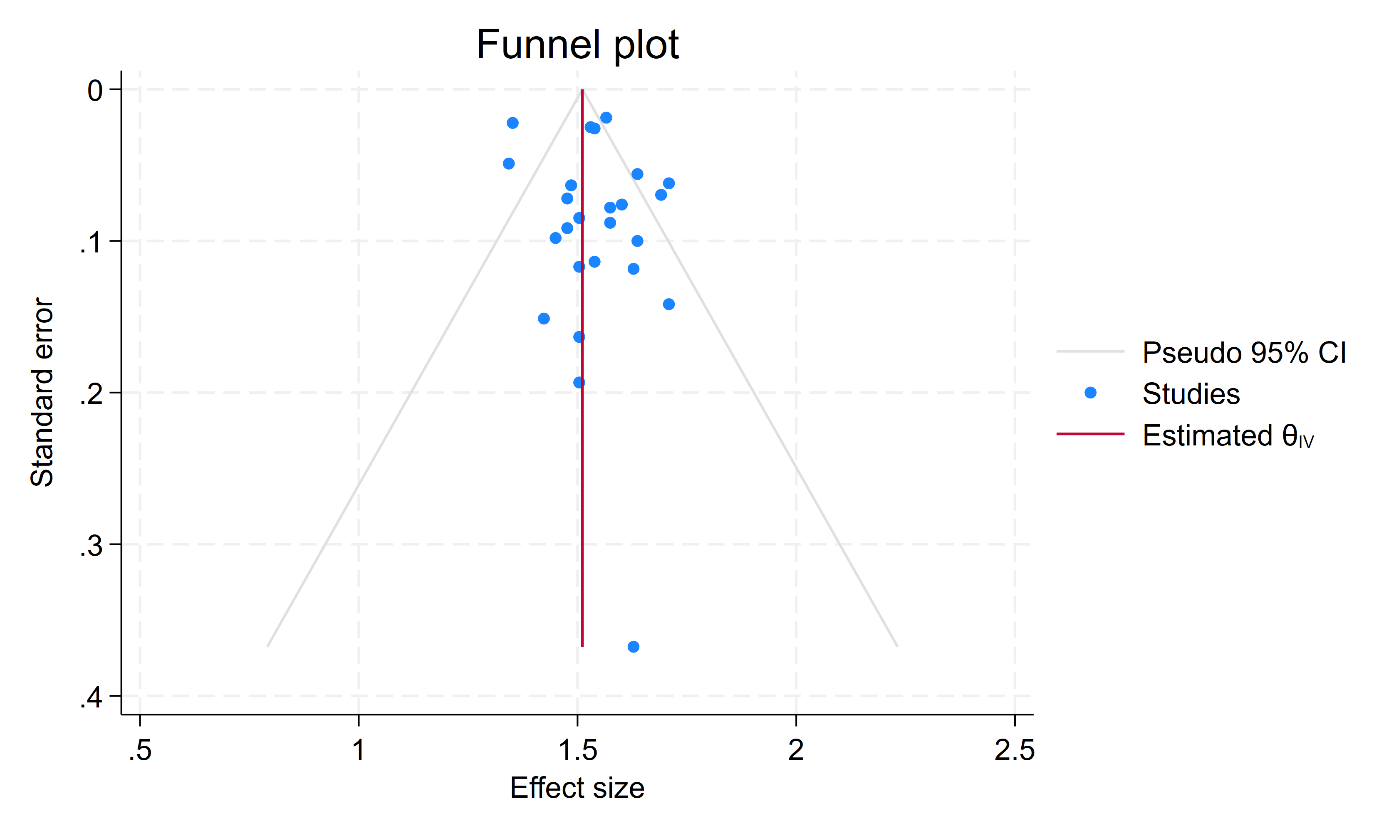


**Supplementary Figure 6C** – Funnel plot of the meta-analysis for pooled average PCr/ATP ratio for patients with heart failure with preserved ejection fraction (HFpEF).


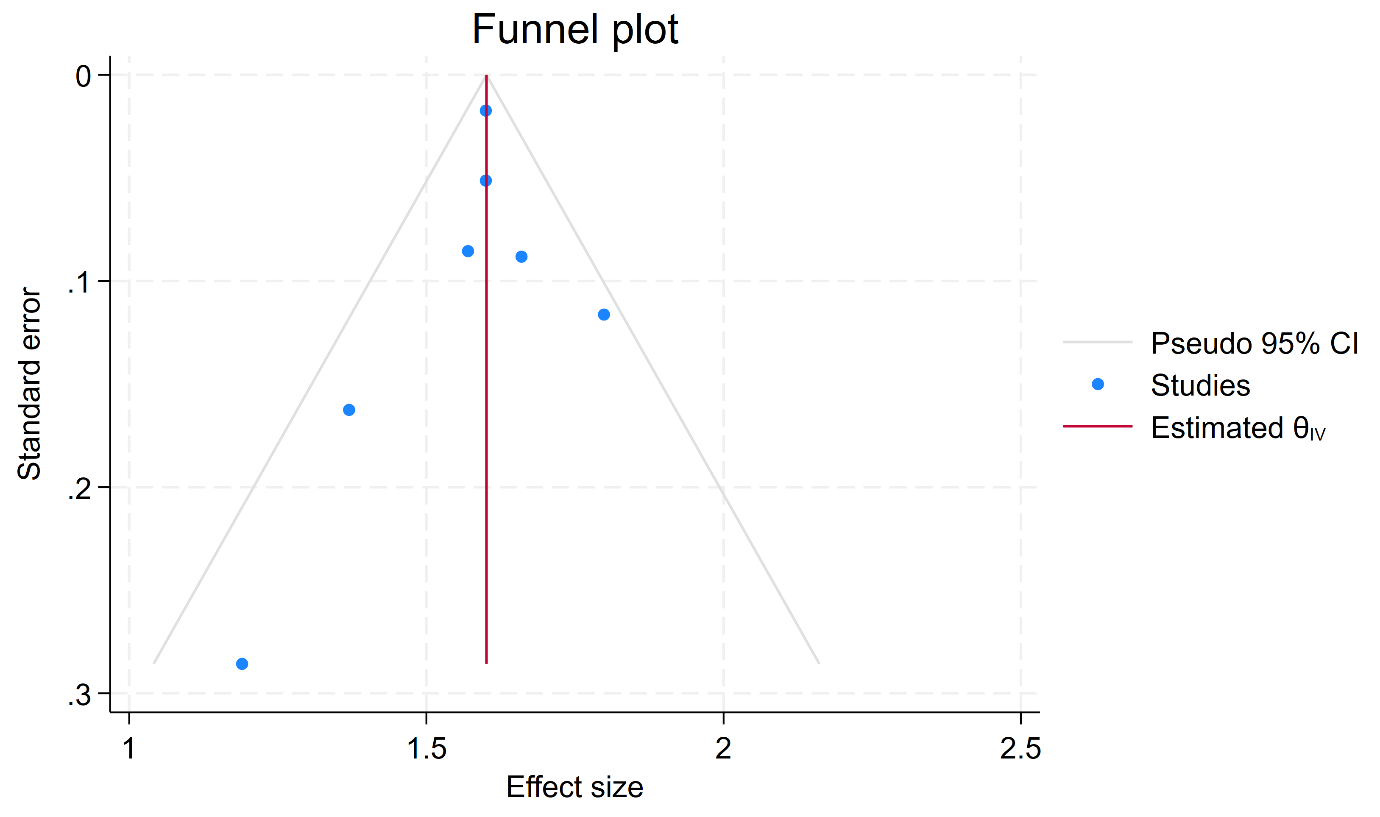


**Supplementary Figure 6D** – Funnel plot of the meta-analysis for pooled average PCr/ATP ratio for patients with moderate aortic stenosis (moderate AS).


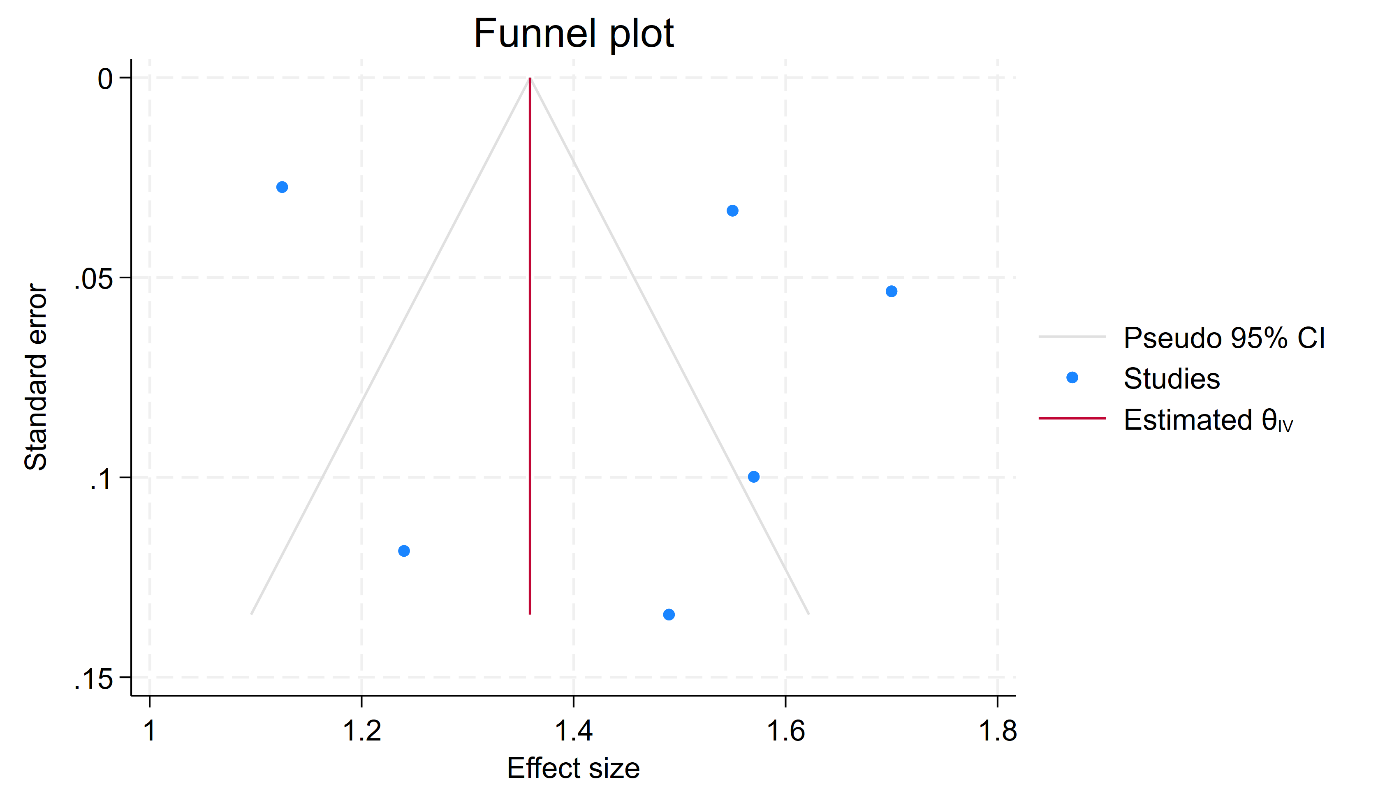


**Supplementary Figure 6E** – Funnel plot of the meta-analysis for pooled average PCr/ATP ratio for patients with severe aortic stenosis (moderate AS). Egger’s regression beta1 =-0.96 (SE 1.44, p=0.51).


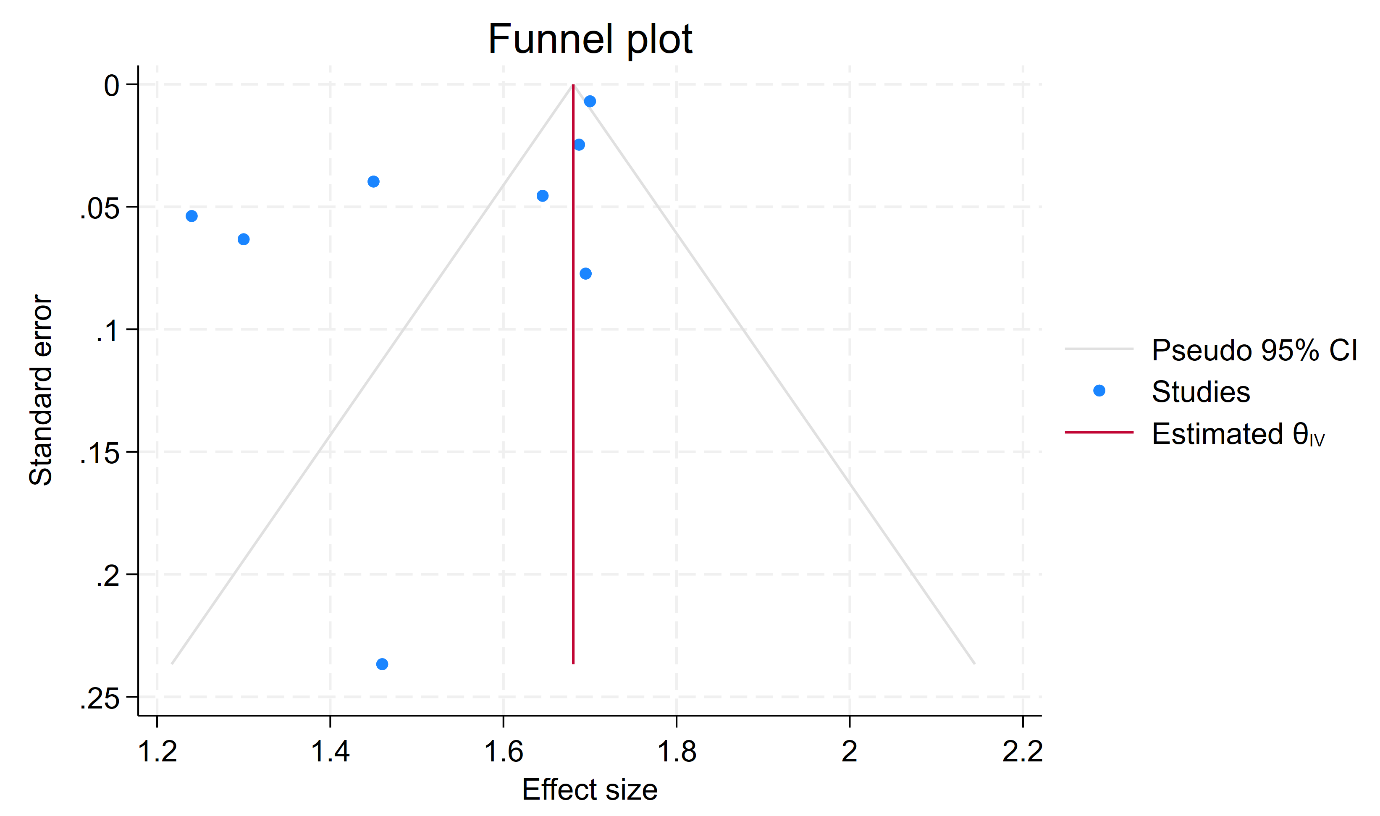


**Supplementary Figure 6F** – Funnel plot of the meta-analysis for pooled average PCr/ATP ratio for patients with type 2 diabetes mellitus (T2DM).


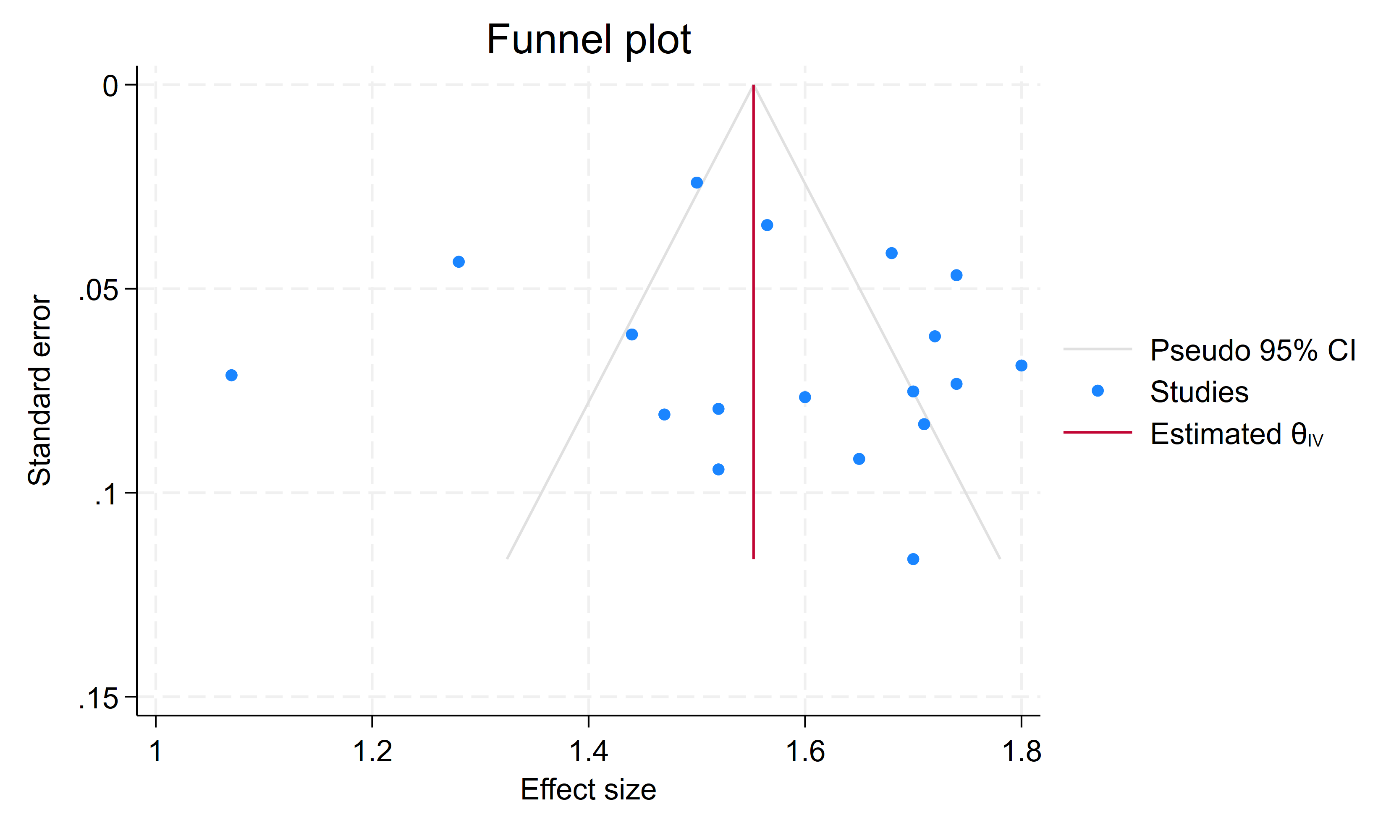


**Supplementary Figure 6G** – Funnel plot of the meta-analysis for pooled average PCr/ATP ratio for patients with type 1 diabetes mellitus (T1DM).


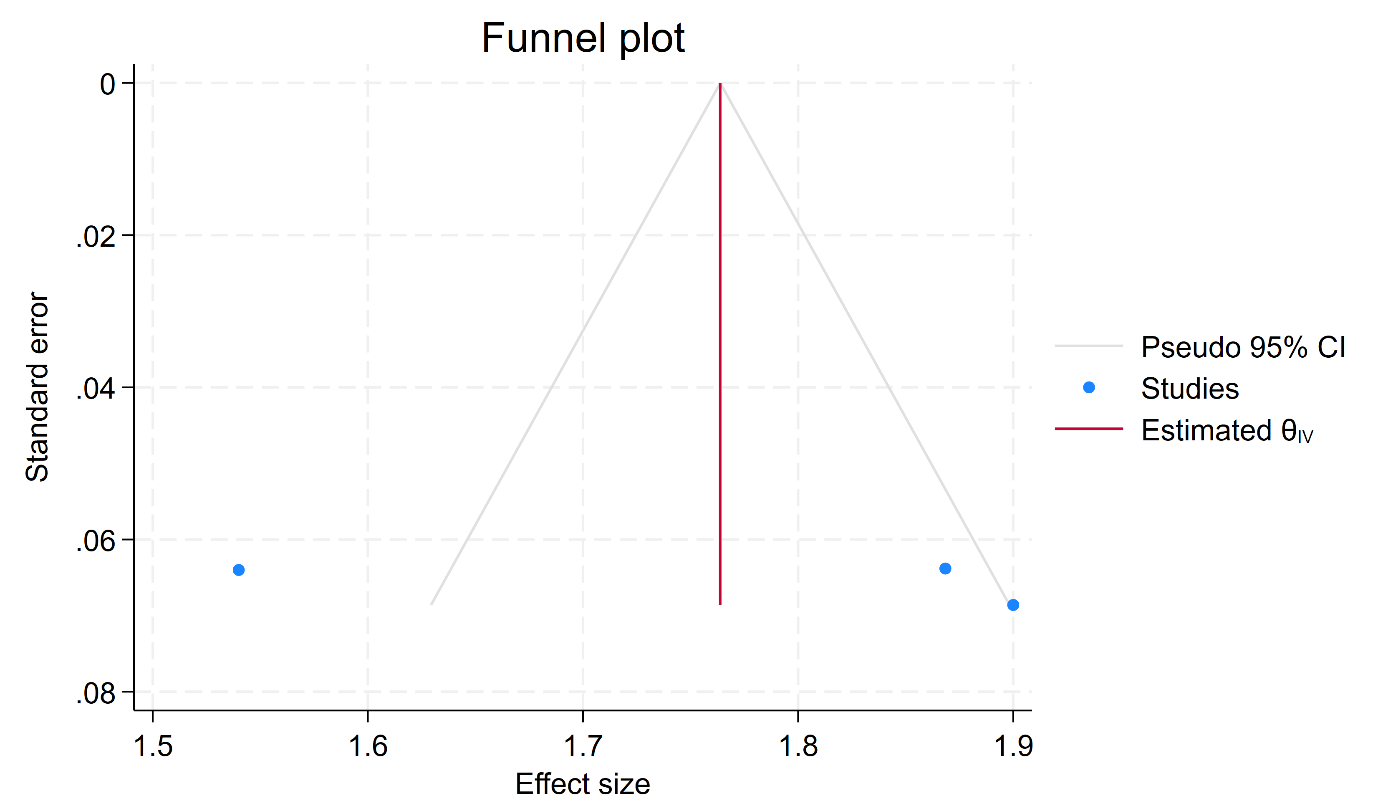


**Supplementary Figure 6H** – Funnel plot of the meta-analysis for pooled average PCr/ATP ratio for patients with type 1 diabetes mellitus (T1DM). Egger’s regression beta1 =3.54 (SE 1.61, p=0.03).


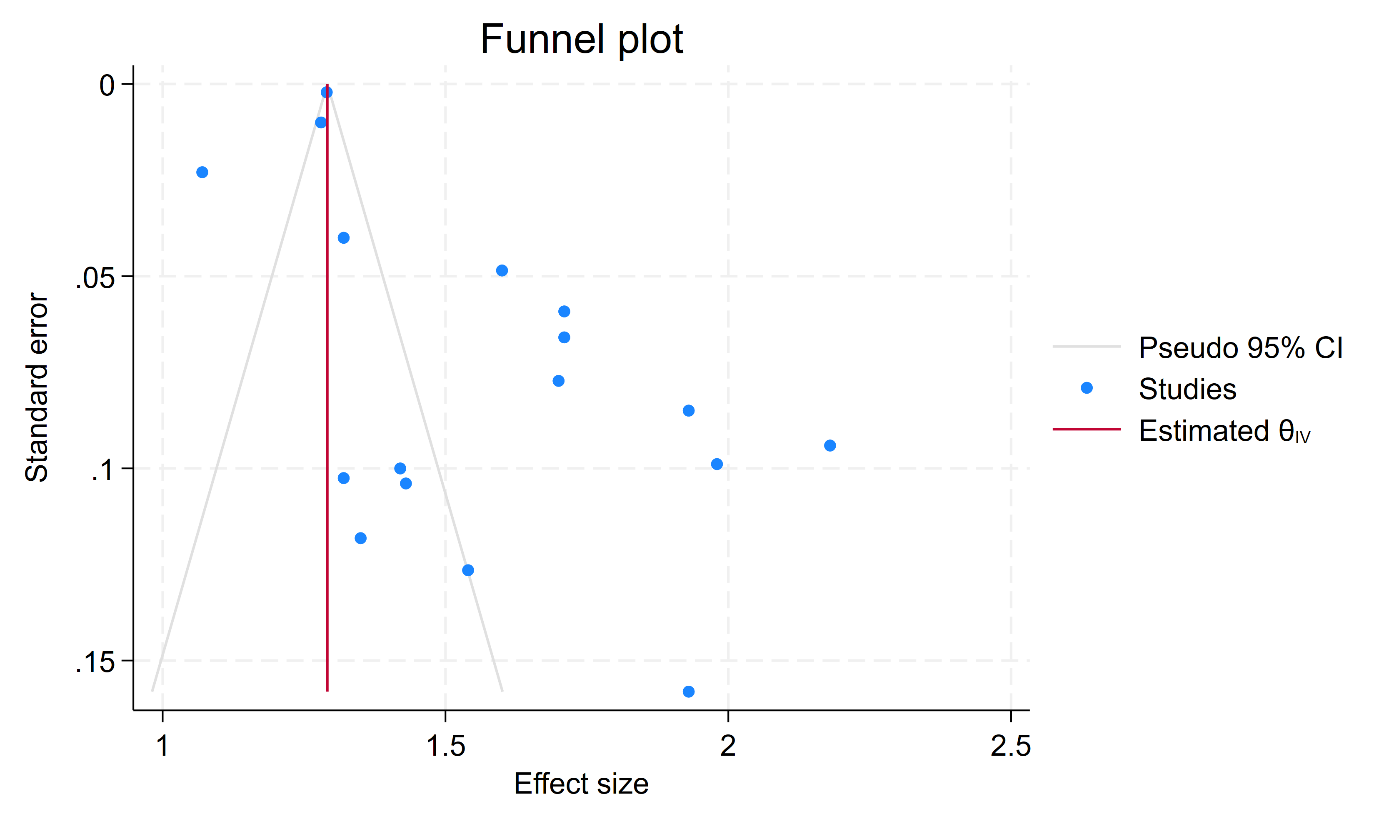


**References**

1. Procter H, Giannoudi M, Kotha S, Jex N, Beech D, Greenwood J, Dweck M, Kellman P, Levelt E. Sex differences in myocardial oxygenation and energetics in patients with severe aortic stenosis without obstructive coronary artery disease before and after aortic valve replacement [published correction appears in CMR 2025 Global CMR Conference. Omni Shoreham Hotel, Washington United States.]. *Journal of Cardiovascular Magnetic Resonance*. 2025;27:101629. doi: <https://dx.doi.org/10.1016/j.jocmr.2024.101629>

2. Perseghin G, Lattuada G, De Cobelli F, Esposito A, Canu T, Ragogna F, Maffi P, Scifo P, Secchi A, Del Maschio A, et al. Left ventricular function and energy homeostasis in patients with type 1 diabetes with and without microvascular complications. *International Journal of Cardiology*. 2012;154:111-115. doi: <https://dx.doi.org/10.1016/j.ijcard.2010.09.010>

3. Ashkir Z, Abd Samat A, Finnigan L, Ahktar MA, Beyhoff N, Sarwar R, Wicks E, Rider O, Valkovic L, Mahmod M, et al. Impaired myocardial energetics in both sarcomere positive and negative HCM are linked to arrhythmic risk. *European heart journal*. 2024;45. doi: 10.1093/eurheartj/ehae666.2054

4. Mannacio V, Di Tommaso L, Stassano P, De Amicis V, Vosa C. Myocardial metabolism and diastolic function after aortic valve replacement for aortic stenosis: influence of patient-prosthesis mismatch. *European journal of cardio-thoracic surgery : official journal of the European Association for Cardio-thoracic Surgery*. 2012;41:316-321. doi: <https://dx.doi.org/10.1016/j.ejcts.2011.05.039>

5. Birkhoelzer S, Portwood C, Pelado J, Mills R, Yavari A, Patel J, Miller J, Mozes F, Neubauer S, Tyler D, et al. EFFECTS OF NINERAFAXSTAT ON MYOCARDIAL ENERGETICS, EXERCISE CAPACITY, AND CARDIAC FUNCTION IN HEART FAILURE WITH PRESERVED EJECTION FRACTION, TYPE 2 DIABETES AND OBESITY- A PHASE 2A CLINICAL TRIAL [published correction appears in American College of Cardiology, (ACC) Meeting 2025. Chicago United States.]. *Journal of the American College of Cardiology*. 2025;85:1164. doi: <https://dx.doi.org/10.1016/S0735-1097%2825%2901648-1>

6. Kuehl M, Stevens MJ, Azam M, Beadle R. Patients with type 2 diabetes demonstrate reduced cardiac energetics and increased lv torsion compared to healthy age matched controls [published correction appears in 72nd Scientific Sessions of the American Diabetes Association. Philadelphia, PA United States. (var.pagings).]. *Diabetes*. 2012;61:A105. doi: <https://dx.doi.org/10.2337/db12-378-655>

7. de Wit-Verheggen VHW, Wefers J, Remie CME, Schrauwen P, Schrauwen-Hinderling VB, van de Weijer T. Cardiac energy metabolism is decreased in male volunteers with prediabetes and does not normalize during the day. *Physiological reports*. 2025;13:e70242. doi: <https://dx.doi.org/10.14814/phy2.70242>

8. Patel RK, Mark PB, Stevens KK, Steedman T, Dargie HJ, Jardine AG. Altered cardiac high energy phosphate metabolism in esrd patients being assessed for renal transplantation [published correction appears in 15th Congress of the European Society for Organ Transplantation, ESOT and 22nd Annual Conference of the British Society for Histocompatibility and Immunogenetics, BSHI. Glasgow United Kingdom. (var.pagings).]. *Transplant International*. 2011;24:138. doi: <https://dx.doi.org/10.1111/j.1432-2277.2011.01350.x>

9. Giannoudi M, Procter H, Kotha S, Thirunavukarasu S, Chowdhary A, Greenwood JP, Jex N, Rider O, Simela C, Plein S, et al. The impact of biological sex and age on the normal ranges of cardiac phosphocreatine to ATP ratio [published correction appears in CMR 2025 Global CMR Conference. Omni Shoreham Hotel, Washington United States.]. *Journal of Cardiovascular Magnetic Resonance*. 2025;27:101657. doi: <https://dx.doi.org/10.1016/j.jocmr.2024.101657>

10. Holloway CJ, Montgomery HE, Murray AJ, Cochlin LE, Codreanu I, Hopwood N, Johnson AW, Rider OJ, Levett DZH, Tyler DJ, et al. Cardiac response to hypobaric hypoxia: Persistent changes in cardiac mass, function, and energy metabolism after a trek to Mt. Everest Base Camp. *FASEB Journal*. 2011;25:792-796. doi: <https://dx.doi.org/10.1096/fj.10-172999>

11. Karkouri J, Watson W, Forner R, Weir-McCall JR, Horn T, Hill M, Hoole S, Klomp D, Rodgers CT. Regionally resolved cardiac metabolism using a dipole-loop array coil for 7 T 31P-MRSI. *Magnetic resonance in medicine*. 2025;94:480-496. doi: <https://dx.doi.org/10.1002/mrm.30492>

12. Holloway CJ, Cochlin LE, Emmanuel Y, Murray A, Codreanu I, Edwards LM, Szmigielski C, Tyler DJ, Knight NS, Saxby BK, et al. A high-fat diet impairs cardiac high-energy phosphate metabolism and cognitive function in healthy human subjects. *American journal of clinical nutrition*. 2011;93:748‐755. doi: 10.3945/ajcn.110.002758

13. Soo CY, Duquenne L, Thornton L, Anderton T, Tomoaia R, Lwin M, Asad M, Kamani C, Chowdhary A, Thirunavukarasu S, et al. Rheumatoid arthritis is associated with sub-clinical abnormalities in myocardial energetics, myocardial oedema, fibrosis, and quantitative perfusion [published correction appears in CMR 2025 Global CMR Conference. Omni Shoreham Hotel, Washington United States.]. *Journal of Cardiovascular Magnetic Resonance*. 2025;27:101632. doi: <https://dx.doi.org/10.1016/j.jocmr.2024.101632>

14. Suttie JJ, Dass S, Bull S, Holloway C, Banerjee R, Cox P, Pitcher A, Franco BR, Francis J, Schneider J, et al. Cardiac steatosis detected in hypertrophic cardiomyopathy, dilated cardiomyopathy and aortic stenosis [published correction appears in American Heart Association's Scientific Sessions 2011. Orlando, FL United States. (var.pagings).]. *Circulation*. 2011;124.

15. Soo CY, Thornton L, Di Donato S, Kakkar V, Bixio R, Tomoaia R, Anderton T, Lwin M, Asad M, Kamani C, et al. Sub-clinical abnormalities in myocardial energetics, perfusion and increased fibrosis in individuals with systemic sclerosis [published correction appears in CMR 2025 Global CMR Conference. Omni Shoreham Hotel, Washington United States.]. *Journal of Cardiovascular Magnetic Resonance*. 2025;27:101631. doi: <https://dx.doi.org/10.1016/j.jocmr.2024.101631>

16. Beadle RM, Williams L, Kuehl M, Bowater S, Abozguia K, Leyva-Leon F, Frenneaux M. Impaired cardiac energetics in dilated cardiomyopathy-magnetic resonance spectroscopy at 3T [published correction appears in European Society of Cardiology, ESC Congress 2011. Paris France. (var.pagings).]. *European heart journal*. 2011;32:1097. doi: <https://dx.doi.org/10.1093/eurheartj/ehr325>

17. Burchert H, Suriano K, Raman B, McCourt A, Cutler H, Leeson P, Fewtrell M, Valkovic L, Lewandowski AJ. Impaired myocardial energetics and lower cardiac reserve in response to exercise stress in adults born preterm [published correction appears in European Society of Cardiology Congress, ESC 2024. London United Kingdom.]. *European heart journal*. 2024;45. doi: <https://dx.doi.org/10.1093/eurheartj/ehae666.2771>

18. Abraham MRR, Bottomley P, Dimaano VL, Pinheiro A, Traill T, Abraham TP, Weiss R. Multimodality imaging reveals decreased creatine-kinase flux and systolic strain in patients with hypertrophic cardiomyopathy and preserved lvef [published correction appears in American Heart Association's Scientific Sessions 2011. Orlando, FL United States. (var.pagings).]. *Circulation*. 2011;124.

19. Thirunavukarasu S, Ansari F, Kotha S, Giannoudi M, Procter H, Cash L, Chowdhary A, Jex N, Shiwani H, Forbes K, et al. Cardiac structural, functional, and energetic assessments during and after pregnancy in women with gestational diabetes mellitus, preeclampsia, and healthy pregnancy. *American journal of obstetrics and gynecology*. 2025;232:565.e561-565.e516. doi: <https://dx.doi.org/10.1016/j.ajog.2024.11.018>

20. Fragasso G, De Cobelli F, Spoladore R, Esposito A, Salerno A, Calori G, Montanaro C, Maranta F, Lattuada G, Margonato A, et al. Resting cardiac energy metabolism is inversely associated with heart rate in healthy young adult men. *American Heart Journal*. 2011;162:136-141. doi: <https://dx.doi.org/10.1016/j.ahj.2011.04.012>

21. Birkhoelzer S, Arvidsson P, Jafarpour M, Henry J, McGing JJ, Tyler D, Rider O, Valkovic L, Rayner J. Cardiac and Skeletal Muscle Energetics in Chronic Heart Failure and Iron Deficiency [published correction appears in CMR 2024 Global CMR Conference. QEII Centre, London United Kingdom.]. *Journal of Cardiovascular Magnetic Resonance*. 2024;26:100836. doi: <https://dx.doi.org/10.1016/j.jocmr.2024.100836>

22. Jones DEJ, Hollingsworth K, Fattakhova G, MacGowan G, Taylor R, Blamire A, Newton JL. Impaired cardiovascular function in primary biliary cirrhosis. *American Journal of Physiology - Gastrointestinal and Liver Physiology*. 2010;298:G764-G773. doi: <https://dx.doi.org/10.1152/ajpgi.00501.2009>

23. Giannoudi M, Procter H, Kotha S, Jex N, Chowdhary A, Thirunavukarasu S, Swoboda P, Plein S, Cubbon RM, Xue H, et al. Cardiac remodeling in moderate aortic stenosis. *medRxiv*. 2024. doi: <https://dx.doi.org/10.1101/2024.02.19.24303060>

24. Shivu GN, Phan TT, Abozguia K, Ahmed I, Wagenmakers A, Henning A, Narendran P, Stevens M, Frenneaux M. Relationship between coronary microvascular dysfunction and cardiac energetics impairment in type 1 diabetes mellitus. *Circulation*. 2010;121:1209-1215. doi: <https://dx.doi.org/10.1161/CIRCULATIONAHA.109.873273>

25. Kotha S, Procter H, Giannoudi M, Plein S, Xue H, Valkovic L, Kellman P, Greenwood J, Clarke K, Levelt E. EFFECTS OF EXOGENOUS KETONE SUPPLEMENTATION ON CARDIAC ENERGETICS, STEATOSIS, FUNCTION, AND PERFUSION IN TYPE 2 DIABETES, HEART FAILURE, AND HEALTHY PARTICIPANTS- A SINGLE CENTER, OPEN LABELLED CLINICAL STUDY [published correction appears in British Cardiovascular Society Annual Conference, BCS 2024. Manchester United Kingdom.]. *Heart*. 2024;110:A145

EP - A147. doi: <https://dx.doi.org/10.1136/heartjnl-2024-BCS.136>

26. Shivu GN, Abozguia K, Phan TT, Ahmed I, Henning A, Frenneaux M. 31P magnetic resonance spectroscopy to measure in vivo cardiac energetics in normal myocardium and hypertrophic cardiomyopathy: Experiences at 3 T. *European Journal of Radiology*. 2010;73:255-259. doi: <https://dx.doi.org/10.1016/j.ejrad.2008.10.018>

27. Kotha S, Giannoudi M, Procter H, Jex N, Thirunavukarasu S, Chowdhary A, Swoboda P, Plein S, Greenwood J, Valkovi L, et al. THE IMPACT OF TYPE 2 DIABETES ON SHORT TERM MYOCARDIAL MORPHOLOGICAL, FUNCTIONAL, AND ENERGETIC RECOVERY POST CORONARY ARTERY BYPASS SURGERY IN PATIENTS WITH ISCHEMIC HEART DISEASE [published correction appears in British Cardiovascular Society Annual Conference, BCS 2024. Manchester United Kingdom.]. *Heart*. 2024;110:A218-A220. doi: <https://dx.doi.org/10.1136/heartjnl-2024-BCS.204>

28. Abozguia K, Elliott P, McKenna W, Phan TT, Nallur-Shivu G, Ahmed I, Maher AR, Kaur K, Taylor J, Henning A, et al. Metabolic modulator perhexiline corrects energy deficiency and improves exercise capacity in symptomatic hypertrophic cardiomyopathy. *Circulation*. 2010;122:1562-1569. doi: <https://dx.doi.org/10.1161/CIRCULATIONAHA.109.934059>

29. Kotha S, Kang SL, Thirunavukarasu S, Procter H, Giannoudi M, Jex N, Chowdhary A, Joseph T, Swoboda P, Plein S, et al. Feasibility of Cardiac 31phosphorus -magnetic Resonance Spectroscopy in Patients with Repaired Tetralogy of Fallot [published correction appears in CMR 2024 Global CMR Conference. QEII Centre, London United Kingdom.]. *Journal of Cardiovascular Magnetic Resonance*. 2024;26:100183. doi: <https://dx.doi.org/10.1016/j.jocmr.2024.100183>

30. Holloway CJ, Cochlin LE, Codreanu I, Bloch E, Fatemianl M, Szmigielski C, Johnson A, Francis JM, Robbins P, Neubauer S, et al. Normobaric hypoxia elevates free fatty acids and impairs cardiac energetics and diastolic function in normal human volunteers [published correction appears in 13th Annual SCMR Scientific Sessions - 2010. Phoenix, AZ United States. (var.pagings).]. *Journal of Cardiovascular Magnetic Resonance*. 2010;12:22.

31. Ng SM, Xavier R, Pan J, Miller J, Mozes F, Valkovic L, Rayner J, Rigolli M, Fronheiser M, Neubauer S, et al. Dynamic Cardiac Magnetic Resonance Measures of Left Atrial Mechanics Across Spectrum of Heart Failure with Preserved Ejection FBaction [published correction appears in CMR 2024 Global CMR Conference. QEII Centre, London United Kingdom.]. *Journal of Cardiovascular Magnetic Resonance*. 2024;26:100446. doi: <https://dx.doi.org/10.1016/j.jocmr.2024.100446>

32. Hollingsworth KG, Jones DEJ, Taylor R, Blamire AM, Newton JL. Impaired cardiovascular response to standing in chronic fatigue syndrome. *European Journal of Clinical Investigation*. 2010;40:608-615. doi: <https://dx.doi.org/10.1111/j.1365-2362.2010.02310.x>

33. Chowdhary A, Thirunavukarasu S, Joseph T, Jex N, Kotha S, Giannoudi M, Procter H, Cash L, Akkaya S, Broadbent D, et al. Liraglutide Improves Myocardial Perfusion and Energetics and Exercise Tolerance in Patients With Type 2 Diabetes. *Journal of the American College of Cardiology*. 2024;84:540‐557. doi: 10.1016/j.jacc.2024.04.064

34. Hudsmith LE, Tyler DJ, Emmanuel Y, Petersen SE, Francis JM, Watkins H, Clarke K, Robson MD, Neubauer S. 31P cardiac magnetic resonance spectroscopy during leg exercise at 3 Tesla. *International Journal of Cardiovascular Imaging*. 2009;25:819-826. doi: <https://dx.doi.org/10.1007/s10554-009-9492-8>

35. Hundertmark MJ, Siu AG, Matthews V, Lewis AJ, Grist J, Patel J, Chamberlin P, Sarwar R, Yavari A, Dehbi HM, et al. Modulating Cardiac Energetics in Cardio-Metabolic Syndromes: A mechanistic, hyperpolarized MR Trial of Ninerafaxstat Treatment. *bioRxiv*. 2024. doi: <https://dx.doi.org/10.1101/2024.04.24.591019>

36. Burkhard T, Herzog C, Linzbach S, Spyridopoulos I, Huebner F, Vogl TJ. Cardiac 31P-MRS compared to echocardiographic findings in patients with hypertensive heart disease without overt systolic dysfunction-Preliminary results. *European Journal of Radiology*. 2009;71:69-74. doi: <https://dx.doi.org/10.1016/j.ejrad.2008.03.022>

37. Chamley RR, Holland JL, Collins J, Pierce K, Watson WD, Green PG, O'Brien D, O'Sullivan O, Barker-Davies R, Ladlow P, et al. Exercise capacity following SARS-CoV-2 infection is related to changes in cardiovascular and lung function in military personnel. *International Journal of Cardiology*. 2024;395:131594. doi: <https://dx.doi.org/10.1016/j.ijcard.2023.131594>

38. Phan TT, Abozguia K, Nallur Shivu G, Mahadevan G, Ahmed I, Williams L, Dwivedi G, Patel K, Steendijk P, Ashrafian H, et al. Heart failure with preserved ejection fraction is characterized by dynamic impairment of active relaxation and contraction of the left ventricle on exercise and associated with myocardial energy deficiency. *Journal of the American College of Cardiology*. 2009;54:402-409. doi: 10.1016/j.jacc.2009.05.012

39. Hundertmark MJ, Adler A, Antoniades C, Coleman R, Griffin JL, Holman RR, Lamlum H, Lee J, Massey D, Miller JJJJ, et al. Assessment of Cardiac Energy Metabolism, Function, and Physiology in Patients With Heart Failure Taking Empagliflozin: The Randomized, Controlled EMPA-VISION Trial. *Circulation*. 2023;147:1654-1669. doi: doi:10.1161/CIRCULATIONAHA.122.062021

40. Esposito A, De Cobelli F, Perseghin G, Pieroni M, Belloni E, Mellone R, Canu T, Gentinetta F, Scifo P, Chimenti C, et al. Impaired left ventricular energy metabolism in patients with hypertrophic cardiomyopathy is related to the extension of fibrosis at delayed gadolinium-enhanced magnetic resonance imaging. *Heart*. 2009;95:228-233. doi: <https://dx.doi.org/10.1136/hrt.2008.142562>

41. Jex N, Greenwood JP, Cubbon RM, Rider OJ, Chowdhary A, Thirunavukarasu S, Kotha S, Giannoudi M, McGrane A, MacCannell A, et al. Association Between Type 2 Diabetes and Changes in Myocardial Structure, Contractile Function, Energetics, and Blood Flow Before and After Aortic Valve Replacement in Patients With Severe Aortic Stenosis. *Circulation*. 2023;148:1138-1153. doi: <https://dx.doi.org/10.1161/CIRCULATIONAHA.122.063444>

42. Bottomley PA, Wu KC, Gerstenblith G, Schulman SP, Steinberg A, Weiss RG. Reduced myocardial creatine kinase flux in human myocardial infarction an in vivo phosphorus magnetic resonance spectroscopy study. *Circulation*. 2009;119:1918-1924. doi: <https://dx.doi.org/10.1161/CIRCULATIONAHA.108.823187>

43. Goldenberg JR, Hays AG, Gabr RE, Schӓr M, Samuel TJ, Yanek LR, Gerstenblith G, Bottomley PA, Weiss RG. Energetic Basis of Recovered Ejection Fraction in Human Heart Failure. *Circulation*. 2023;148:1976-1978. doi: 10.1161/circulationaha.123.065217

44. Tyler DJ, Emmanuel Y, Cochlin LE, Hudsmith LE, Holloway CJ, Neubauer S, Clarke K, Robson MD. Reproducibility of <SUP>31</SUP>P cardiac magnetic resonance spectroscopy at 3 T. *NMR IN BIOMEDICINE*. 2009;22:405-413. doi: 10.1002/nbm.1350

45. Watson WD, Green PG, Lewis AJM, Arvidsson P, De Maria GL, Arheden H, Heiberg E, Clarke WT, Rodgers CT, Valkovic L, et al. Retained Metabolic Flexibility of the Failing Human Heart. *Circulation*. 2023;148:109-123. doi: <https://dx.doi.org/10.1161/CIRCULATIONAHA.122.062166>

46. Beer M, Wagner D, Myers J, Sandstede J, Kostler H, Hahn D, Neubauer S, Dubach P. Effects of Exercise Training on Myocardial Energy Metabolism and Ventricular Function Assessed by Quantitative Phosphorus-31 Magnetic Resonance Spectroscopy and Magnetic Resonance Imaging in Dilated Cardiomyopathy. *Journal of the American College of Cardiology*. 2008;51:1883-1891. doi: <https://dx.doi.org/10.1016/j.jacc.2007.09.075>

47. Monga S, Valkovič L, Myerson SG, Neubauer S, Mahmod M, Rider OJ. Role of Cardiac Energetics in Aortic Stenosis Disease Progression: Identifying the High-risk Metabolic Phenotype. *Circulation Cardiovascular imaging*. 2023;16:e014863. doi: 10.1161/circimaging.122.014863

48. Perseghin G, Lattuada G, De Cobelli F, Esposito A, Belloni E, Ntali G, Ragogna F, Canu T, Scifo P, Del Maschio A, et al. Increased mediastinal fat and impaired left ventricular energy metabolism in young men with newly found fatty liver. *Hepatology*. 2008;47:51-58. doi: <https://dx.doi.org/10.1002/hep.21983>

49. van de Bovenkamp AA, Geurkink KTJ, Oosterveer FTP, de Man FS, Kok WEM, Bronzwaer PNA, Allaart CP, Nederveen AJ, van Rossum AC, Bakermans AJ, et al. Trimetazidine in heart failure with preserved ejection fraction: a randomized controlled cross-over trial. *ESC heart failure*. 2023;10:2998‐3010. doi: 10.1002/ehf2.14418

50. Wolf C, Boesch S, Metzler B, Weirich-Schwaiger H, Trieb T, Schocke MFH. Phosphorus-31 two-dimensional chemical shift imaging in the myocardium of patients with late onset of Friedreich ataxia. *Molecular Imaging and Biology*. 2008;10:24-29. doi: <https://dx.doi.org/10.1007/s11307-007-0119-y>

51. Gamble DT, Ross J, Khan H, Unger A, Cheyne L, Rudd A, Saunders F, Srivanasan J, Kamya S, Horgan G, et al. Impaired Cardiac and Skeletal Muscle Energetics Following Anthracycline Therapy for Breast Cancer. *Circulation Cardiovascular imaging*. 2023;16:e015782. doi: 10.1161/circimaging.123.015782

52. Klug G, Zwick RH, Frick M, Wolf C, Schocke MFH, Conci E, Jaschke W, Pachinger O, Metzler B. Impact of exercise capacity on myocardial high-energy phosphate metabolism. *International journal of sports medicine*. 2007;28:667-672. doi: <https://dx.doi.org/10.1055/s-2007-964862>

53. Papalia F, Jouhra F, Amin-Youssef G, Shah AM, Charles-Edwards G, Okonko DO. Cardiac energetics in patients with chronic heart failure and iron deficiency: an <i>in-vivo</i> <SUP>31</SUP>P magnetic resonance spectroscopy study. *EUROPEAN JOURNAL OF HEART FAILURE*. 2022;24:716-723. doi: 10.1002/ejhf.2454

54. Perseghin G, Ntali G, De Cobelli F, Lattuada G, Esposito A, Belloni E, Canu T, Costantino F, Ragogna F, Scifo P, et al. Abnormal left ventricular energy metabolism in obese men with preserved systolic and diastolic functions is associated with insulin resistance. *Diabetes Care*. 2007;30:1520-1526. doi: <https://dx.doi.org/10.2337/dc06-2429>

55. Gorecka M, Jex N, Thirunavukarasu S, Chowdhary A, Corrado J, Davison J, Tarrant R, Poenar A-M, Sharrack N, Parkin A, et al. Cardiovascular magnetic resonance imaging and spectroscopy in clinical long-COVID-19 syndrome: a prospective case-control study. *Journal of cardiovascular magnetic resonance : official journal of the Society for Cardiovascular Magnetic Resonance*. 2022;24:50. doi: <https://dx.doi.org/10.1186/s12968-022-00887-9>

56. Beer M, Machann W, Sandstede J, Buchner S, Lipke C, Kostler H, Lorenz R, Harre K, Spindler M, Hahn D. Energetic differences between viable and non-viable myocardium in patients with recent myocardial infarction are not an effect of differences in wall thinning - A multivoxel 31P-MR-spectroscopy and MRI study. *European Radiology*. 2007;17:1275-1283. doi: <https://dx.doi.org/10.1007/s00330-006-0492-y>

57. Jex N, Chowdhary A, Thirunavukarasu S, Procter H, Sengupta A, Natarajan P, Kotha S, Poenar A-M, Swoboda P, Xue H, et al. Coexistent Diabetes Is Associated With the Presence of Adverse Phenotypic Features in Patients With Hypertrophic Cardiomyopathy. *Diabetes care*. 2022;45:1852-1862. doi: <https://dx.doi.org/10.2337/dc22-0083>

58. Van Der Meer RW, Hammer S, Smit JWA, Frolich M, Bax JJ, Diamant M, Rijzewijk LJ, De Roos A, Romijn JA, Lamb HJ. Short-term caloric restriction induces accumulation of myocardial triglycerides and decreases left ventricular diastolic function in healthy subjects. *Diabetes*. 2007;56:2849-2853. doi: <https://dx.doi.org/10.2337/db07-0768>

59. Reid A, Miller C, Farrant JP, Polturi R, Clark D, Ray S, Cooper G, Schmitt M. Copper chelation in patients with hypertrophic cardiomyopathy. *Open Heart*. 2022;9:e001803. doi: <https://dx.doi.org/10.1136/openhrt-2021-001803>

60. Heyne JP, Rzanny R, Hansch A, Leder U, Reichenbach JR, Kaiser WA. 31P-MR spectroscopic imaging in hypertensive heart disease. *European Radiology*. 2006;16:1796-1802. doi: <https://dx.doi.org/10.1007/s00330-006-0170-0>

61. Cameron D, Soto-Mota A, Willis DR, Ellis J, Procter NEK, Greenwood R, Saunders N, Schulte RF, Vassiliou VS, Tyler DJ, et al. Evaluation of Acute Supplementation With the Ketone Ester (<i>R</i>)-3-Hydroxybutyl-(R)-3-Hydroxybutyrate (deltaG) in Healthy Volunteers by Cardiac and Skeletal Muscle <SUP>31</SUP>P Magnetic Resonance Spectroscopy. *FRONTIERS IN PHYSIOLOGY*. 2022;13. doi: 10.3389/fphys.2022.793987

62. Caus T, Kober F, Marin P, Mouly-Bandini A, Quilici J, Métras D, Cozzone PJ, Bernard M. Non-invasive diagnostic of cardiac allograft vasculopathy by <SUP>31</SUP>P magnetic resonance chemical shift imaging. *EUROPEAN JOURNAL OF CARDIO-THORACIC SURGERY*. 2006;29:45-49. doi: 10.1016/j.ejcts.2005.10.038

63. Valkovič L, Apps A, Ellis J, Neubauer S, Tyler DJ, Schmid AI, Rider OJ, Rodgers CT. Increased cardiac Pi/PCr in the diabetic heart observed using phosphorus magnetic resonance spectroscopy at 7T. *PLOS ONE*. 2022;17:e0269957. doi: 10.1371/journal.pone.0269957

64. Perseghin G, Fiorina P, De Cobelli F, Scifo P, Esposito A, Canu T, Danna M, Gremizzi C, Secchi A, Luzi L, et al. Cross-sectional assessment of the effect of kidney and kidney-pancreas transplantation on resting left ventricular energy metabolism in type 1 diabetic-uremic patients - A phosphorous-31 magnetic resonance spectroscopy study. *Journal of the American College of Cardiology*. 2005;46:1085-1092. doi: 10.1016/j.jacc.2005.05.075

65. Chowdhary A, Cubbon R, Thirunavukarasu S, Jex N, Kotha S, Xue H, Kellman P, Greenwood J, Plein S, Levelt E. Body mass index associated differences in cardiac stress energetics in type 2 diabetes [published correction appears in European Respiratory Society International Congress, ERS 2022. Barcelona Spain.]. *European Respiratory Journal*. 2022;60:280. doi: <https://dx.doi.org/10.1093/eurheartj/ehac544.280>

66. Najjar SS, Bottomley PA, Schulman SP, Waldron MM, Steffen RP, Gerstenblith G, Weiss RG. Effects of a pharmacologically-induced shift of hemoglobin-oxygen dissociation on myocardial energetics during ischemia in patients with coronary artery disease. *Journal of Cardiovascular Magnetic Resonance*. 2005;7:657-666. doi: <https://dx.doi.org/10.1081/JCMR-65610>

67. Chowdhary A, Javed W, Thirunavukarasu S, Jex N, Kotha S, Kellman P, Swoboda P, Greenwood JP, Plein S, Levelt E. Cardiac Adaptations to Acute Hemodynamic Stress in Function, Perfusion, and Energetics in Type 2 Diabetes With Overweight and Obesity. *Diabetes Care*. 2022;45:e176-e178. doi: 10.2337/dc22-0887

68. Chida K, Otani H, Saito H, Nagasaka T, Kagaya Y, Kohzuki M, Zuguchi M, Shirato K. Feasibility of rapid-sequence <SUP>31</SUP>P magnetic resonance spectroscopy in cardiac patients. *ACTA RADIOLOGICA*. 2005;46:386-390. doi: 10.1080/02841850510021283

69. Henry JA, Levelt E, Rayner JJ, Hundertmark MJ, Peterzan MA, Green PG, Watson W, Burrage MK, Arvidsson P, Lewis AJM, et al. Investigating myocardial energetic deficit across the spectrum of cardiac disease. *European heart journal*. 2022;43. doi: 10.1093/eurheartj/ehac544.244

70. Hansch A, Rzanny R, Heyne JP, Leder U, Reichenbach JR, Kaiser WA. Noninvasive measurements of cardiac high-energy phosphate metabolites in dilated cardiomyopathy by using <SUP>31</SUP>P spectroscopic chemical shift imaging. *EUROPEAN RADIOLOGY*. 2005;15:319-323. doi: 10.1007/s00330-004-2504-0

71. Wibowo PG, Charman SJ, Okwose NC, Velicki L, Popovic D, Hollingsworth KG, Macgowan GA, Jakovljevic DG. Association between cardiac high-energy phosphate metabolism and whole body metabolism in healthy female adults. *Physiological research*. 2021;70:393-399.

72. Schocke MFH, Zoller H, Vogel W, Wolf C, Kremser C, Steinboeck P, Poelzl G, Pachinger O, Jaschke WR, Metzler B. Cardiac phosphorus-31 two-dimensional chemical shift imaging in patients with hereditary hemochromatosis. *Magnetic Resonance Imaging*. 2004;22:515-521. doi: <https://dx.doi.org/10.1016/j.mri.2004.01.023>

73. Chowdhary A, Thirunavukarasu S, Jex N, Bowers C, Cubbon R, Xue H, Kellman P, Greenwood JP, Plein S, Levelt E. Coronary microvascular dysfunction is only detectable in type 2 diabetes in the presence of obesity [published correction appears in European Society of Cardiology Congress, ESC 2021. Virtual.]. *European heart journal*. 2021;42:237. doi: <https://dx.doi.org/10.1093/eurheartj/ehab724.237>

74. Beer M, Spindler M, Sandstede JJW, Remmert H, Beer S, Kostler H, Hahn D. Detection of myocardial infarctions by acquisition-weighted 31P-MR spectroscopy in humans. *Journal of magnetic resonance imaging : JMRI*. 2004;20:798-802. doi: <https://dx.doi.org/10.1002/jmri.20185>

75. Thirunavukarasu S, Jex N, Chowdhary A, Hassan IU, Straw S, Craven TP, Gorecka M, Broadbent D, Swoboda P, Witte KK, et al. Empagliflozin Treatment Is Associated With Improvements in Cardiac Energetics and Function and Reductions in Myocardial Cellular Volume in Patients With Type 2 Diabetes. *Diabetes*. 2021;70:2810-2822. doi: 10.2337/db21-0270

76. Lodi R, Rajagopalan B, Blamire AM, Crilley JG, Styles P, Chinnery PF. Abnormal cardiac energetics in patients carrying the A3243G mtDNA mutation measured in vivo using phosphorus MR spectroscopy. *Biochimica et Biophysica Acta - Bioenergetics*. 2004;1657:146-150. doi: <https://dx.doi.org/10.1016/j.bbabio.2004.05.003>

77. Burrage MK, Hundertmark M, Valkovič L, Watson WD, Rayner J, Sabharwal N, Ferreira VM, Neubauer S, Miller JJ, Rider OJ, et al. Energetic Basis for Exercise-Induced Pulmonary Congestion in Heart Failure With Preserved Ejection Fraction. *Circulation*. 2021;144:1664-1678. doi: 10.1161/circulationaha.121.054858

78. Schocke MF, Martinek M, Kremser C, Wolf C, Steinboeck P, Lechleitner M, Jaschke W, Pachinger O, Metzler B. 3-hydroxy-3-methylglutaryl coenzyme A reductase inhibitors improve myocardial high-energy phosphate metabolism in men. *Journal of cardiovascular magnetic resonance : official journal of the Society for Cardiovascular Magnetic Resonance*. 2003;5:595-602. doi: <https://dx.doi.org/10.1081/jcmr-120025234>

79. Gaborit B, Ancel P, Abdullah AE, Maurice F, Abdesselam I, Calen A, Soghomonian A, Houssays M, Varlet I, Eisinger M, et al. Effect of empagliflozin on ectopic fat stores and myocardial energetics in type 2 diabetes: the EMPACEF study. *Cardiovasc Diabetol*. 2021;20:57. doi: 10.1186/s12933-021-01237-2

80. Schocke MFH, Metzler B, Wolf C, Steinboeck P, Kremser C, Pachinger O, Jaschke W, Lukas P. Impact of aging on cardiac high-energy phosphate metabolism determined by phosphorus-31 2-dimensional chemical shift imaging (31P 2D CSI). *Magnetic Resonance Imaging*. 2003;21:553-559. doi: <https://dx.doi.org/10.1016/S0730-725X%2803%2900079-1>

81. Watson WD, Green PG, Valkovic L, Herring N, Neubauer S, Rider OJ. Myocardial Energy Response to Glyceryl Trinitrate: Physiology Revisited. *Frontiers in Physiology*. 2021;12:790525. doi: <https://dx.doi.org/10.3389/fphys.2021.790525>

82. Scheuermann-Freestone M, Madsen PL, Manners D, Blamire AM, Buckingham RE, Styles P, Radda GK, Neubauer S, Clarke K. Abnormal cardiac and skeletal muscle energy metabolism in patients with type 2 diabetes. *Circulation*. 2003;107:3040-3046. doi: <https://dx.doi.org/10.1161/01.CIR.0000072789.89096.10>

83. Rayner JJ, Peterzan MA, Clarke WT, Rodgers CT, Neubauer S, Rider OJ. Obesity modifies the energetic phenotype of dilated cardiomyopathy. *European heart journal*. 2021;43:868-877. doi: 10.1093/eurheartj/ehab663

84. Ogimoto G, Sakurada T, Imamura K, Kuboshima S, Maeba T, Kimura K, Owada S. Alteration of energy production by the heart in CRF patients undergoing peritoneal dialysis. *Molecular and Cellular Biochemistry*. 2003;244:135-138. doi: <https://dx.doi.org/10.1023/A:1022470815270>

85. Apps A, Valkovic L, Peterzan M, Lau JYC, Hundertmark M, Clarke W, Tunnicliffe EM, Ellis J, Tyler DJ, Neubauer S, et al. Quantifying the effect of dobutamine stress on myocardial Pi and pH in healthy volunteers: A 31P MRS study at 7T. *Magnetic Resonance in Medicine*. 2021;85:1147-1159. doi: <https://dx.doi.org/10.1002/mrm.28494>

86. Diamant M, Lamb HJ, Groeneveld Y, Endert EL, Smit JWA, Bax JJ, Romijn JA, De Roos A, Radder JK. Diastolic dysfunction is associated with altered myocardial metabolism in asymptomatic normotensive patients with well-controlled type 2 diabetes mellitus. *Journal of the American College of Cardiology*. 2003;42:328-335. doi: <https://dx.doi.org/10.1016/S0735-1097%2803%2900625-9>

87. Lewis AJM, Abdesselam I, Rayner JJ, Byrne J, Borlaug BA, Neubauer S, Rider OJ. Adverse right ventricular remodelling, function, and stress responses in obesity: insights from cardiovascular magnetic resonance *European Heart Journal - Cardiovascular Imaging*. 2021;23:1383-1390. doi: 10.1093/ehjci/jeab175

88. Crilley JG, Boehm EA, Blair E, Rajagopalan B, Blamire AM, Styles P, McKenna WJ, Ostman-Smith I, Clarke K, Watkins H. Hypertrophic cardiomyopathy due to sarcomeric gene mutations is characterized by impaired energy metabolism irrespective of the degree of hypertrophy. *Journal of the American College of Cardiology*. 2003;41:1776-1782. doi: <https://dx.doi.org/10.1016/S0735-1097%2802%2903009-7>

89. Rayner JJ, Peterzan MA, Watson WD, Clarke WT, Neubauer S, Rodgers CT, Rider OJ. Myocardial Energetics in Obesity. *Circulation*. 2020;141:1152-1163. doi: doi:10.1161/CIRCULATIONAHA.119.042770

90. Spindler M, Weilbach F, Beer M, Sandstede J, Kostler H, Strotmann J, Voelker W, Hahn D, Ertl G, Gold R. Non-Invasive Functional and Biochemical Assessment of Mitoxantrone Cardiotoxicity in Patients with Multiple Sclerosis. *Journal of Cardiovascular Pharmacology*. 2003;42:680-687. doi: <https://dx.doi.org/10.1097/00005344-200311000-00015>

91. Rider OJ, Apps A, Miller J, Lau JYC, Lewis AJM, Peterzan MA, Dodd MS, Lau AZ, Trumper C, Gallagher FA, et al. Noninvasive In Vivo Assessment of Cardiac Metabolism in the Healthy and Diabetic Human Heart Using Hyperpolarized (13)C MRI. *Circ Res*. 2020;126:725-736. doi: 10.1161/circresaha.119.316260

92. Beer M, Seyfarth T, Sandstede J, Landschütz W, Lipke C, Köstler H, von Kienlin M, Harre K, Hahn D, Neubauer S. Absolute concentrations of high-energy phosphate metabolites in normal, hypertrophied, and failing human myocardium measured noninvasively with <SUP>31</SUP>P-SLOOP magnetic resonance spectroscopy. *Journal of the American College of Cardiology*. 2002;40:1267-1274. doi: 10.1016/S0735-1097(02)02160-5

93. Peterzan MA, Clarke WT, Lygate CA, Lake HA, Lau JYC, Miller JJ, Johnson E, Rayner JJ, Hundertmark MJ, Sayeed R, et al. Cardiac Energetics in Patients With Aortic Stenosis and Preserved Versus Reduced Ejection Fraction. *Circulation*. 2020;141:1971-1985. doi: <https://dx.doi.org/10.1161/CIRCULATIONAHA.119.043450>

94. Beer M, Schneider C, Köstler H, Buchner S, Sandstede J, Toyka KV, Hahn D. Quantitative analysis of energy metabolism in human muscle using SLOOP <SUP>31</SUP>P-MR-spectroscopy. *ROFO-FORTSCHRITTE AUF DEM GEBIET DER RONTGENSTRAHLEN UND DER BILDGEBENDEN VERFAHREN*. 2002;174:573-578. doi: 10.1055/s-2002-28273

95. Ellis J, Valkovic L, Purvis LAB, Clarke WT, Rodgers CT. Reproducibility of human cardiac phosphorus MRS (<SUP>31</SUP>P-MRS) at 7 T. *NMR IN BIOMEDICINE*. 2019;32. doi: 10.1002/nbm.4095

96. Metzler B, Schocke MFH, Steinboeck P, Wolf C, Judmaier W, Lechleitner M, Lukas P, Pachinger O. Decreased high-energy phosphate ratios in the myocardium of men with diabetes mellitus type I. *Journal of Cardiovascular Magnetic Resonance*. 2002;4:493-502. doi: <https://dx.doi.org/10.1081/JCMR-120016387>

97. De Cobelli F, Rossini A, Esposito A, Canu T, Manzoni G, Del Maschio A, Rubinacci A, Sirtori M, Losa M, Lanzi R, et al. Short-term evaluation of cardiac morphology, function, metabolism and structure following diagnosis of adult-onset growth hormone deficiency. *Growth Hormone and IGF Research*. 2019;46:50-54. doi: <https://dx.doi.org/10.1016/j.ghir.2019.06.003>

98. Pohmann R, Von Kienlin M. Accurate phosphorus metabolite images of the human heart by 3D acquisition-weighted CSI. *Magnetic Resonance in Medicine*. 2001;45:817-826. doi: <https://dx.doi.org/10.1002/mrm.1110>

99. Stoll VM, Hess AT, Rodgers CT, Bissell MM, Dyverfeldt P, Ebbers T, Myerson SG, Carlhäll CJ, Neubauer S. Left Ventricular Flow Analysis. *Circulation Cardiovascular imaging*. 2019;12:e008130. doi: 10.1161/circimaging.118.008130

100. Beyerbacht HP, Lamb HJ, Van Der Laarse A, Vliegen HW, Leujes F, Hazekamp MC, De Roos A, Van Der Wall EE. Aortic valve replacement in patients with aortic valve stenosis improves myocardial metabolism and diastolic function. *Radiology*. 2001;219:637-643. doi: <http://dx.doi.org/10.1148/radiology.219.3.r01jn25637>

101. Nathania M, Hollingsworth KG, Bates M, Eggett C, Trenell MI, Velicki L, Seferovic PM, MacGowan GA, Turnbull DM, Jakovljevic DG. Impact of age on the association between cardiac high-energy phosphate metabolism and cardiac power in women. *Heart*. 2018;104:111-118. doi: <https://dx.doi.org/10.1136/heartjnl-2017-311275>

102. Lodi R, Rajagopalan B, Blamire AM, Cooper JM, Davies CH, Bradley JL, Styles P, Schapira AH. Cardiac energetics are abnormal in Friedreich ataxia patients in the absence of cardiac dysfunction and hypertrophy: an in vivo 31P magnetic resonance spectroscopy study [published correction appears in Comment in: Cardiovasc Res. 2002 Jun;54(3):694; author reply 695-6 PMID: 12031716 [<https://www.ncbi.nlm.nih.gov/pubmed/12031716>]]. *Cardiovascular research*. 2001;52:111-119. doi: <https://dx.doi.org/10.1016/s0008-6363(01)00357-1>

103. Mahmod M, Pal N, Rayner J, Holloway C, Raman B, Dass S, Levelt E, Ariga R, Ferreira V, Banerjee R, et al. The interplay between metabolic alterations, diastolic strain rate and exercise capacity in mild heart failure with preserved ejection fraction: a cardiovascular magnetic resonance study. *J Cardiovasc Magn Reson*. 2018;20:88. doi: 10.1186/s12968-018-0511-6

104. Moka D, Baer FM, Theissen P, Schneider CA, Dietlein M, Erdmann E, Schicha H. Non-Q-wave myocardial infarction: Impaired myocardial energy metabolism in regions with reduced 99mTc-MIBI accumulation. *European Journal of Nuclear Medicine*. 2001;28:602-607. doi: <https://dx.doi.org/10.1007/s002590100500>

105. Scally C, Rudd A, Mezincescu A, Wilson H, Srivanasan J, Horgan G, Broadhurst P, Newby DE, Henning A, Dawson DK. Persistent long-term structural, functional, and metabolic changes after stress-induced (takotsubo) cardiomyopathy. *Circulation*. 2018;137:1039-1048. doi: <https://dx.doi.org/10.1161/CIRCULATIONAHA.117.031841>

106. Beer M, Sandstede J, Landschütz W, Viehrig M, Harre K, Horn M, Meininger M, Pabst T, Kenn W, Haase A, et al. Altered energy metabolism after myocardial infarction assessed by <SUP>31</SUP>P-MR-spectroscopy in humans. *EUROPEAN RADIOLOGY*. 2000;10:1323-1328. doi: 10.1007/s003300000316

107. Peterzan MA, Lygate CA, Lake HA, Rayner JJ, Hundertmark MJ, Apps AP, Sayeed RA, Petrou M, Krasopoulos G, Neubauer S, et al. Reduced myocardial ATP delivery in severe primary mitral regurgitation: A novel marker to guide timing of surgery? [published correction appears in European Society of Cardiology Congress, ESC 2018. Munich Germany.]. *European heart journal*. 2018;39:1275. doi: <https://dx.doi.org/10.1093/eurheartj/ehy566.6161>

108. Crilley JG, Boehm EA, Rajagopalan B, Blamire AM, Styles P, Muntoni F, Hilton-Jones D, Clarke K. Magnetic resonance spectroscopy evidence of abnormal cardiac energetics in Xp21 muscular dystrophy. *Journal of the American College of Cardiology*. 2000;36:1953-1958. doi: <https://dx.doi.org/10.1016/S0735-1097%2800%2900960-8>

109. Rayner JJ, Banerjee R, Holloway CJ, Lewis AJM, Peterzan MA, Francis JM, Neubauer S, Rider OJ. The relative contribution of metabolic and structural abnormalities to diastolic dysfunction in obesity. *International Journal of Obesity*. 2018;42:441-447. doi: <https://dx.doi.org/10.1038/ijo.2017.239>

110. Eidenschink AB, Schroter G, Muller-Weihrich S, Stern H. Myocardial high-energy phosphate metabolism is altered after treatment with anthracycline in childhood. *Cardiology in the young*. 2000;10:610-617.

111. Valkovic L, Clarke WT, Purvis LAB, Schaller B, Robson MD, Rodgers CT. Adiabatic excitation for 31P MR spectroscopy in the human heart at 7 T: A feasibility study. *Magnetic Resonance in Medicine*. 2017;78:1667-1673. doi: <https://dx.doi.org/10.1002/mrm.26576>

112. Buchthal SD, Noureuil TO, Den Hollander JA, Bourge RC, Kirklin JK, Katholi CR, Caulfield JB, Pohost GM, Evanochko WT. 31P-magnetic resonance spectroscopy studies of cardiac transplant patients at rest. *Journal of Cardiovascular Magnetic Resonance*. 2000;2:51-56. doi: <http://dx.doi.org/10.3109/10976640009148673>

113. Stoll VM, Clarke WT, Levelt E, Liu A, Myerson SG, Robson MD, Neubauer S, Rodgers CT. Dilated cardiomyopathy: Phosphorus 31 MR spectroscopy at 7 T. *Radiology*. 2016;281:409-417. doi: <https://dx.doi.org/10.1148/radiol.2016152629>

114. Pluim BM, Swenne CA, Zwinderman AH, Maan AC, Van Der Laarse A, Doornbos J, Van Der Wall EE. Correlation of heart rate variability with cardiac functional and metabolic variables in cyclists with training induced left ventricular hypertrophy. *Heart*. 1999;81:612-617. doi: <http://dx.doi.org/10.1136/hrt.81.6.612>

115. Bowater SE, Weaver RA, Beadle RM, Frenneaux MP, Marshall JM, Clift PF. Assessment of the Physiological Adaptations to Chronic Hypoxemia in Eisenmenger Syndrome. *Congenital Heart Disease*. 2016;11:341-347. doi: <https://dx.doi.org/10.1111/chd.12373>

116. Lamb HJ, Beyerbacht HP, van der Laarse A, Stoel BC, Doornbos J, van der Wall EE, de Roos A. Diastolic dysfunction in hypertensive heart disease is associated with altered myocardial metabolism. *Circulation*. 1999;99:2261-2267. doi: 10.1161/01.cir.99.17.2261

117. Levelt E, Rodgers CT, Clarke WT, Mahmod M, Ariga R, Francis JM, Liu A, Wijesurendra RS, Dass S, Sabharwal N, et al. Cardiac energetics, oxygenation, and perfusion during increased workload in patients with type 2 diabetes mellitus. *European heart journal*. 2016;37:3461-3469. doi: <https://dx.doi.org/10.1093/eurheartj/ehv442>

118. Nanbu T, Nakakoshi T, Yonezawa K, Kitabatake A. Myocardial high-energy phosphate metabolism in patients with stable chronic dilated cardiomyopathy under a dobutamine-induced prolonged mild workload. *American Heart Journal*. 1999;138:641-645. doi: <https://dx.doi.org/10.1016/S0002-8703%2899%2970177-8>

119. Levelt E, Mahmod M, Piechnik SK, Ariga R, Francis JM, Rodgers CT, Clarke WT, Sabharwal N, Schneider JE, Karamitsos TD, et al. Relationship between left ventricular structural and metabolic remodeling in type 2 diabetes. *Diabetes*. 2016;65:44-52. doi: <https://dx.doi.org/10.2337/db15-0627>

120. Jung WI, Sieverding L, Breuer J, Hoess T, Widmaier S, Schmidt O, Bunse M, Van Erckelens F, Apitz J, Lutz O, et al. 31P NMR spectroscopy detects metabolic abnormalities in asymptomatic patients with hypertrophic cardiomyopathy. *Circulation*. 1998;97:2536-2542. doi: <http://dx.doi.org/10.1161/01.CIR.97.25.2536>

121. Wijesurendra R, Liu A, Eichhorn C, Levelt E, Ariga R, Clarke W, Rodgers C, Bashir Y, Ginks M, Rajappan K, et al. Atrial fibrillation is associated with left ventricular dysfunction and impaired myocardial energetics that persist despite catheter ablation: is atrial fibrillation the chicken or the EGG? [published correction appears in British Cardiovascular Society Annual Conference 2016. Manchester United Kingdom.]. *Heart*. 2016;102:A98-A100. doi: <https://dx.doi.org/10.1136/heartjnl-2016-309890.138>

122. Tagami T, Sakuma H, Matsumura K, Takeda K, Mori S, Takeuchi T, Nakano T. Evaluation of altered myocardial high energy phosphate metabolism in patients on maintenance dialysis using phosphorus-31 magnetic resonance spectroscopy. *Investigative radiology*. 1998;33:171-176. doi: <https://dx.doi.org/10.1097/00004424-199803000-00007>

123. Salerno A, Fragasso G, Esposito A, Canu T, Lattuada G, Manzoni G, Del Maschio A, Margonato A, De Cobelli F, Perseghin G. Effects of short-term manipulation of serum FFA concentrations on left ventricular energy metabolism and function in patients with heart failure: no association with circulating bio-markers of inflammation. *Acta Diabetologica*. 2015;52:753-761. doi: <https://dx.doi.org/10.1007/s00592-014-0695-7>

124. Pluim BM, Lamb HJ, Kayser HW, Leujes F, Beyerbacht HP, Zwinderman AH, van der Laarse A, Vliegen HW, de Roos A, van der Wall EE. Functional and metabolic evaluation of the athlete's heart by magnetic resonance imaging and dobutamine stress magnetic resonance spectroscopy. *Circulation*. 1998;97:666-672. doi: <https://dx.doi.org/10.1161/01.cir.97.7.666>

125. Dass S, Holloway CJ, Cochlin LE, Rider OJ, Mahmod M, Robson M, Sever E, Clarke K, Watkins H, Ashrafian H, et al. No Evidence of Myocardial Oxygen Deprivation in Nonischemic Heart Failure. *Circ Heart Fail*. 2015;8:1088-1093. doi: 10.1161/circheartfailure.114.002169

126. Loffler R, Sauter R, Kolem H, Haase A, von Kienlin M. Localized spectroscopy from anatomically matched compartments: improved sensitivity and localization for cardiac 31P MRS in humans. *Journal of magnetic resonance (San Diego, Calif : 1997)*. 1998;134:287-299.

127. Dass S, Cochlin LE, Suttie JJ, Holloway CJ, Rider OJ, Carden L, Tyler DJ, Karamitsos TD, Clarke K, Neubauer S, et al. Exacerbation of cardiac energetic impairment during exercise in hypertrophic cardiomyopathy: A potential mechanism for diastolic dysfunction. *European heart journal*. 2015;36:1547-1554. doi: <https://dx.doi.org/10.1093/eurheartj/ehv120>

128. Conway MA, Bottomley PA, Ouwerkerk R, Radda GK, Rajagopalan B. Mitral regurgitation - Impaired systolic function, eccentric hypertrophy, and increased severity are linked to lower phosphocreatine/ATP ratios in humans. *Circulation*. 1998;97:1716-1723. doi: 10.1161/01.CIR.97.17.1716

129. Ntusi NA, Holloway C, Francis JM, Davis A, Levelt E, Piechnik SK, Ferreira VM, Matthews PM, Wordsworth PB, Karamitsos TD, et al. Impaired energetics and normal myocardial lipids in rheumatoid arthritis and systemic lupus erythematosus: A phosphorous and proton magnetic resonance spectroscopy and cardiovascular magnetic resonance study [published correction appears in 18th Annual SCMR Scientific Sessions. Nice France. (var.pagings).]. *Journal of Cardiovascular Magnetic Resonance*. 2015;17.

130. Neubauer S, Horn M, Cramer M, Harre K, Newell JB, Peters W, Pabst T, Ertl G, Hahn D, Ingwall JS, et al. Myocardial Phosphocreatine-to-ATP Ratio Is a Predictor of Mortality in Patients With Dilated Cardiomyopathy. *Circulation*. 1997;96:2190-2196. doi: doi:10.1161/01.CIR.96.7.2190

131. Beadle RM, Williams LK, Kuehl M, Bowater S, Abozguia K, Leyva F, Yousef Z, Wagenmakers AJ, Thies F, Horowitz J, et al. Improvement in cardiac energetics by perhexiline in heart failure due to dilated cardiomyopathy. *JACC Heart Fail*. 2015;3:202-211. doi: 10.1016/j.jchf.2014.09.009

132. Neubauer S, Horn M, Pabst T, Harre K, Stromer H, Bertsch G, Sandstede J, Ertl G, Hahn D, Kochsiek K. Cardiac high-energy phosphate metabolism in patients with aortic valve disease assessed by 31P-magnetic resonance spectroscopy. *Journal of investigative medicine : the official publication of the American Federation for Clinical Research*. 1997;45:453-462.

133. Madathil A, Hollingsworth KG, Blamire AM, Razvi S, Newton JL, Taylor R, Weaver JU. Levothyroxine improves abnormal cardiac bioenergetics in subclinical hypothyroidism: A cardiac magnetic resonance spectroscopic study. *Journal of Clinical Endocrinology and Metabolism*. 2015;100:E607-E610. doi: <https://dx.doi.org/10.1210/jc.2014-2942>

134. Kalil R, deAlbuquerque CP, Weiss RG, Mocelim A, Bellotti G, Cerri G, Pileggi F. Normal high energy phosphate ratios in ''stunned'' human myocardium. *Journal of the American College of Cardiology*. 1997;30:1228-1232. doi: 10.1016/S0735-1097(97)00306-9

135. Geier O, Weng AM, Toepell A, Hahn D, Spindler M, Beer M, Köestler H. Acquisition-weighted chemical shift imaging improves SLOOP quantification of human cardiac phosphorus metabolites. *ZEITSCHRIFT FUR MEDIZINISCHE PHYSIK*. 2014;24:49-54. doi: 10.1016/j.zemedi.2013.01.002

136. Sieverding L, Jung WI, Breuer J, Widmaier S, Staubert A, Van Erckelens F, Schmidt O, Bunse M, Hoess T, Lutz O, et al. Proton-decoupled myocardial 31P NMR spectroscopy reveals decreased PCR/P(i) in patients with severe hypertrophic cardiomyopathy. *American Journal of Cardiology*. 1997;80:34A-40A. doi: <https://dx.doi.org/10.1016/S0002-9149%2897%2900456-6>

137. Dass S, Ariga R, Sever E, Cochlin LE, Suttie J, Holloway C, Mahmod M, Karamitsos TD, Neubauer S. Cardiac energy metabolism and oxygenation during exercise in the hypertensive heart [published correction appears in 17th Annual SCMR Scientific Sessions. New Orleans, LA United States. (var.pagings).]. *Journal of Cardiovascular Magnetic Resonance*. 2014;16.

138. Hochachka PW, Clark CM, Holden JE, Stanley C, Ugurbil K, Menon RS. 31P magnetic resonance spectroscopy of the Sherpa heart: a phosphocreatine/adenosine triphosphate signature of metabolic defense against hypobaric hypoxia. *Proceedings of the National Academy of Sciences of the United States of America*. 1996;93:1215-1220. doi: <https://dx.doi.org/10.1073/pnas.93.3.1215>

139. Esterhammer R, Klug G, Wolf C, Mayr A, Reinstadler S, Feistritzer H-J, Metzler B, Schocke MFH. Cardiac high-energy phosphate metabolism alters with age as studied in 196 healthy males with the help of 31-phosphorus 2-dimensional chemical shift imaging. *PloS one*. 2014;9:e97368. doi: <https://dx.doi.org/10.1371/journal.pone.0097368>

140. Pluim BM, Chin JC, De Roos A, Doornbos J, Siebelink HM, Van der Laarse A, Vliegen HW, Lamerichs RM, Bruschke AV, Van der Wall EE. Cardiac anatomy, function and metabolism in elite cyclists assessed by magnetic resonance imaging and spectroscopy [published correction appears in Comment in: Eur Heart J. 1996 Aug;17(8):1138-40 PMID: 8869851 [<https://www.ncbi.nlm.nih.gov/pubmed/8869851>]]. *European heart journal*. 1996;17:1271-1278. doi: <https://dx.doi.org/10.1093/oxfordjournals.eurheartj.a015046>

141. Cassidy SA, Hallsworth K, Thoma C, Hollingsworth K, Jakovljevic J, Trenell MI. Cardiac structure and function is altered in adults with metabolic disorders [published correction appears in Diabetes UK Professional Conference 2014. Liverpool United Kingdom. (var.pagings).]. *Diabetic Medicine*. 2014;31:36-37. doi: <https://dx.doi.org/10.1111/dme.12378_1>

142. Lamb HJ, Doornbos J, Den Hollander JA, Luyten PR, Beyerbacht HP, Van der Wall EE, De Roos A. Reproducibility of human cardiac 31P-NMR spectroscopy. *NMR in Biomedicine*. 1996;9:217-227. doi: <https://dx.doi.org/10.1002/%28SICI%291099-1492%28199608%299:5%3C217::AID-NBM419%3E3.0.CO;2-G>

143. Jakovljevic DG, Papakonstantinou L, Blamire AM, MacGowan GA, Taylor R, Hollingsworth KG, Trenell MI. Effect of physical activity on age-related changes in cardiac function and performance in women. *Circulation: Cardiovascular Imaging*. 2014;8:e002086. doi: <https://dx.doi.org/10.1161/CIRCIMAGING.114.002086>

144. Hetherington HP, Luney DJ, Vaughan JT, Pan JW, Ponder SL, Tschendel O, Twieg DB, Pohost GM. 3D 31P spectroscopic imaging of the human heart at 4.1 T. *Magnetic resonance in medicine*. 1995;33:427-431. doi: <https://dx.doi.org/10.1002/mrm.1910330318>

145. Rodgers CT, Clarke WT, Snyder C, Vaughan JT, Neubauer S, Robson MD. Human cardiac 31P magnetic resonance spectroscopy at 7 tesla. *Magnetic Resonance in Medicine*. 2014;72:304-315. doi: <https://dx.doi.org/10.1002/mrm.24922>

146. Nishiyama T. Correlation between cardiac function and cardiac high-energy phosphate metabolism assessed by 31P-MRS in patients with dilated cardiomyopathy. *Journal of Saitama Medical School*. 1995;22:99-107.

147. Mahmod M, Francis JM, Pal N, Lewis A, Dass S, De Silva R, Petrou M, Sayeed R, Westaby S, Robson MD, et al. Myocardial perfusion and oxygenation are impaired during stress in severe aortic stenosis and correlate with impaired energetics and subclinical left ventricular dysfunction. *Journal of Cardiovascular Magnetic Resonance*. 2014;16:29. doi: <https://dx.doi.org/10.1186/1532-429X-16-29>

148. Yabe T, Mitsunami K, Okada M, Morikawa S, Inubushi T, Kinoshita M. Detection of myocardial ischemia by 31P magnetic resonance spectroscopy during handgrip exercise. *Circulation*. 1994;89:1709-1716. doi: <http://dx.doi.org/10.1161/01.CIR.89.4.1709>

149. Bashir A, Gropler R. Reproducibility of creatine kinase reaction kinetics in human heart: A 31P time-dependent saturation transfer spectroscopy study. *NMR in Biomedicine*. 2014;27:663-671. doi: <https://dx.doi.org/10.1002/nbm.3103>

150. Kuno S, Ogawa T, Katsuta S, Itai Y. IN-VIVO HUMAN MYOCARDIAL-METABOLISM DURING AEROBIC EXERCISE BY P-31 NUCLEAR-MAGNETIC-RESONANCE SPECTROSCOPY. *EUROPEAN JOURNAL OF APPLIED PHYSIOLOGY AND OCCUPATIONAL PHYSIOLOGY*. 1994;69:488-491. doi: 10.1007/BF00239864

151. Lewis AJM, Finlayson S, Mahmod M, Karamitsos TD, Dass S, Ashrafian H, Francis JM, Watkins H, Beeson D, Palace J, et al. A novel cardiac phenotype in patients with GFPT1 or DPAGT1 mutations. *Experimental and Clinical Cardiology*. 2014;20:3139-3145.

152. Doornbos J, Luyten PR, Janssen M, Wasser M, de Roos A. P-31 MR spectroscopy of skeletal and cardiac muscle metabolism in patients with systemic sclerosis: a multiple case study. *Journal of magnetic resonance imaging : JMRI*. 1994;4:165-168.

153. Hollingsworth KG, Willis TA, Bates MGD, Dixon BJ, Lochmuller H, Bushby K, Bourke J, MacGowan GA, Straub V. Subepicardial dysfunction leads to global left ventricular systolic impairment in patients with limb girdle muscular dystrophy 2I. *European Journal of Heart Failure*. 2013;15:986-994. doi: <https://dx.doi.org/10.1093/eurjhf/hft057>

154. van Dobbenburgh JO, Lekkerkerk C, van Echteld CJ, de Beer R. Saturation correction in human cardiac 31P MR spectroscopy at 1.5 T. *NMR in biomedicine*. 1994;7:218-224.

155. Bates MGD, Newman JH, Jakovljevic DG, Hollingsworth KG, Alston CL, Zalewski P, Klawe JJ, Blamire AM, Macgowan GA, Keavney BD, et al. Defining cardiac adaptations and safety of endurance training in patients with m.3243A>G-related mitochondrial disease. *International Journal of Cardiology*. 2013;168:3599-3608. doi: <https://dx.doi.org/10.1016/j.ijcard.2013.05.062>

156. Sakuma H, Takeda K, Tagami T, Nakagawa T, Okamoto S, Konishi T, Nakano T. 31P MR spectroscopy in hypertrophic cardiomyopathy: Comparison with Tl-201 myocardial perfusion imaging. *American Heart Journal*. 1993;125:1323-1328. doi: <https://dx.doi.org/10.1016/0002-8703%2893%2991002-V>

157. Betim Paes Leme AM, Salemi VM, Weiss RG, Parga JR, Ianni BM, Mady C, Kalil-Filho R. Exercise-induced decrease in myocardial high-energy phosphate metabolites in patients with Chagas heart disease. *Journal of cardiac failure*. 2013;19:454-460.

158. Neubauer S, Krahe T, Schindler R, Horn M, Hillenbrand H, Entzeroth C, Mader H, Kromer EP, Riegger GA, Lackner K, et al. 31P magnetic resonance spectroscopy in dilated cardiomyopathy and coronary artery disease. Altered cardiac high-energy phosphate metabolism in heart failure. *Circulation*. 1992;86:1810-1818. doi: 10.1161/01.cir.86.6.1810

159. Banks L, Wells GD, McKillop A, Jean-St-Michel E, Redington AN, McCrindle BW. Exploring the effects of chronic remote ischemic preconditioning versus high-intensity exercise on improving peak exercise capacity and cardiac energy metabolism: A randomized, crossover clinical trial [published correction appears in 66th Annual Meeting of the Canadian Cardiovascular Society. Montreal, QC Canada. (var.pagings).]. *Canadian Journal of Cardiology*. 2013;29:S338. doi: <https://dx.doi.org/10.1016/j.cjca.2013.07.576>

160. de Roos A, Doornbos J, Luyten PR, Oosterwaal LJ, van der Wall EE, den Hollander JA. Cardiac metabolism in patients with dilated and hypertrophic cardiomyopathy: assessment with proton-decoupled P-31 MR spectroscopy. *Journal of magnetic resonance imaging : JMRI*. 1992;2:711-719.

161. Banks L, Wells GD, McCrindle BW. Cardiac energy metabolism is positively associated with skeletal muscle energy metabolism in physically active adolescents and young adults. *Applied physiology, nutrition, and metabolism = Physiologie appliquee, nutrition et metabolisme*. 2014;39:363-368. doi: <https://dx.doi.org/10.1139/apnm-2013-0312>

162. Masuda Y, Tateno Y, Ikehira H, Hashimoto T, Shishido F, Sekiya M, Imazeki Y, Imai H, Watanabe S, Inagaki Y. High-energy phosphate metabolism of the myocardium in normal subjects and patients with various cardiomyopathies--the study using ECG gated MR spectroscopy with a localization technique. *Japanese circulation journal*. 1992;56:620-626. doi: <https://dx.doi.org/10.1253/jcj.56.620>

163. Rider OJ, Francis JM, Tyler D, Byrne J, Clarke K, Neubauer S. Effects of weight loss on myocardial energetics and diastolic function in obesity. *International Journal of Cardiovascular Imaging*. 2013;29:1043-1050. doi: <https://dx.doi.org/10.1007/s10554-012-0174-6>

164. Schaefer S, Schwartz GG, Steinman SK, Meyerhoff DJ, Massie BM, Weiner MW. Metabolic response of the human heart to inotropic stimulation: in vivo phosphorus-31 studies of normal and cardiomyopathic myocardium. *Magnetic resonance in medicine : official journal of the Society of Magnetic Resonance in Medicine / Society of Magnetic Resonance in Medicine*. 1992;25:260-272.

165. Spoladore R, Fragasso G, Perseghin G, De Cobelli F, Esposito A, Maranta F, Calori G, Locatelli M, Lattuada G, Scifo P, et al. Beneficial effects of beta-blockers on left ventricular function and cellular energy reserve in patients with heart failure. *Fundamental & Clinical Pharmacology*. 2013;27:455-464. doi: <https://doi.org/10.1111/j.1472-8206.2012.01029.x>

166. Conway MA, Allis J, Ouwerkerk R, Niioka T, Rajagopalan B, Radda GK. Detection of low phosphocreatine to ATP ratio in failing hypertrophied human myocardium by 31P magnetic resonance spectroscopy. *Lancet*. 1991;338:973-976. doi: <https://dx.doi.org/10.1016/0140-6736%2891%2991838-L>

167. Hirsch GA, Bottomley PA, Gerstenblith G, Weiss RG. Allopurinol acutely increases adenosine triphospate energy delivery in failing human hearts. *Journal of the American College of Cardiology*. 2012;59:802-808. doi: <https://dx.doi.org/10.1016/j.jacc.2011.10.895>

168. Auffermann W, Chew WM, Wolfe CL, Tavares NJ, Parmley WW, Semelka RC, Donnelly T, Chatterjee K, Higgins CB. NORMAL AND DIFFUSELY ABNORMAL MYOCARDIUM IN HUMANS - FUNCTIONAL AND METABOLIC CHARACTERIZATION WITH P-31 MR SPECTROSCOPY AND CINE MR IMAGING. *RADIOLOGY*. 1991;179:253-259. doi: 10.1148/radiology.179.1.2006287

169. Rider OJ, Francis JM, Ali MK, Holloway C, Pegg T, Robson MD, Tyler D, Byrne J, Clarke K, Neubauer S. Effects of catecholamine stress on diastolic function and myocardial energetics in obesity. *Circulation*. 2012;125:1511-1519.

170. Schaefer S, Gober JR, Schwartz GG, Twieg DB, Weiner MW, Massie B. INVIVO P-31 SPECTROSCOPIC IMAGING IN PATIENTS WITH GLOBAL MYOCARDIAL-DISEASE. *AMERICAN JOURNAL OF CARDIOLOGY*. 1990;65:1154-1161. doi: 10.1016/0002-9149(90)90331-T

171. Mazaev VV, Stukalova OV, Gabrusenko SA, Ageev FT, Ternovoy SK, Chazova IE. High energy myocardial metabolism in patients with different causes of left ventricular hypertrophy by 31P magnetic resonance spectroscopy [published correction appears in ESC Congress 2012. Munchen Germany. (var.pagings).]. *European heart journal*. 2012;33:812. doi: <https://dx.doi.org/10.1093/eurheartj/ehs283>

172. Weiss RG, Bottomley PA, Hardy CJ, Gerstenblith G. Regional myocardial metabolism of high-energy phosphates during isometric exercise in patients with coronary artery disease. *New England Journal of Medicine*. 1990;323:1593-1600.

173. Holloway CJ, Dass S, Suttie JJ, Rider OJ, Cox P, Cochlin LE, Jackson H, Fast AM, Johnson AW, Karamitsos TD, et al. Exercise training in dilated cardiomyopathy improves rest and stress cardiac function without changes in cardiac high energy phosphate metabolism. *Heart*. 2012;98:1083-1090. doi: <https://dx.doi.org/10.1136/heartjnl-2012-302145>

174. Schaefer S, Gober J, Valenza M, Karczmar GS, Matson GB, Camacho SA, Botvinick EH, Massie B, Weiner MW. Nuclear magnetic resonance imaging-guided phosphorus-31 spectroscopy of the human heart. *Journal of the American College of Cardiology*. 1988;12:1449-1455. doi: <https://dx.doi.org/10.1016/s0735-1097(88)80008-1>

175. Malatesta-Muncher R, Wansapura J, Taylor M, Lindquist D, Hor K, Mitsnefes M. Early cardiac dysfunction in pediatric patients on maintenance dialysis and post kidney transplant. *Pediatric Nephrology*. 2012;27:1157-1164. doi: <https://dx.doi.org/10.1007/s00467-012-2124-x>

176. Rajagopalan B, Blackledge MJ, McKenna WJ, Bolas N, Radda GK. Measurement of phosphocreatine to ATP ratio in normal and diseased human heart by 31P magnetic resonance spectroscopy using the rotating frame-depth selection technique. *Annals of the New York Academy of Sciences*. 1987;508:321-332.
